# Electrophysiological Correlates of Cognitive Dysfunction in Obsessive–Compulsive Disorder (OCD): A Systematic and Mechanistic Review of EEG, ERP, and QEEG Evidence

**DOI:** 10.3390/jcm15155994

**Published:** 2026-08-01

**Authors:** James Chmiel, Aleksandra Kładna

**Affiliations:** 1 Faculty of Physical Culture and Health, Institute of Physical Culture Sciences, University of Szczecin, Al. Piastów 40B Blok 6, 71-065 Szczecin, Poland; 2 Department of the History of Medicine and Medical Ethics, Pomeranian Medical University, ul. Rybacka 1, 70-204 Szczecin, Poland

**Keywords:** obsessive–compulsive disorder, EEG, event-related potentials, cognitive dysfunction, executive function, inhibitory control, error monitoring, P300, ERN

## Abstract

**Background/Objectives:** Obsessive–compulsive disorder (OCD) is associated not only with obsessions and compulsions, but also with cognitive dysfunction involving inhibitory control, cognitive flexibility, working memory, attention, decision-making, feedback learning, and performance monitoring. Electroencephalography (EEG), event-related potentials (ERPs), quantitative EEG, and time–frequency analyses provide temporally precise methods for examining how these cognitive abnormalities unfold during information processing. This review aimed to synthesise electrophysiological evidence on the neural correlates of cognitive dysfunction in OCD. **Methods:** A systematic literature search was conducted in PubMed/MEDLINE, Scopus, Web of Science Core Collection, Embase, PsycINFO, and Google Scholar from database inception to 30 May 2026. Eligible studies included patients with OCD and used EEG, qEEG, spectral EEG, EEG connectivity, or ERP methods in relation to cognitive performance, cognitive task demands, or cognitive dysfunction. Studies were grouped according to electrophysiological modality and ERP component. Because of methodological heterogeneity, findings were synthesised narratively and mechanistically rather than by meta-analysis. Methodological quality was assessed using ROBINS-I. **Results:** The search identified 1321 records, of which 42 studies met the inclusion criteria. Most studies used task-based ERP paradigms, while fewer examined resting-state EEG, qEEG, or oscillatory activity. The most consistent findings concerned altered performance monitoring and cognitive control, especially ERN, N2/N200, Pe, Pc, and P3/P300 abnormalities. ERN findings suggested excessive early error monitoring, whereas N2/N200 and P3/P300 findings indicated task-dependent abnormalities in inhibition, conflict processing, attentional allocation, stimulus evaluation, and context updating. Earlier components, including P50, N1/N100, P2/P200, and N450, suggested abnormalities in sensory gating, early attentional selection, stimulus evaluation, and interference control, particularly under emotionally salient or OCD-relevant conditions. FRN findings indicated altered feedback processing and reinforcement learning, while limited P600 evidence suggested inefficient working-memory preparation. Plain EEG and time–frequency studies further implicated abnormal theta, alpha, beta, and delta activity in monitoring, inhibition, arousal, and network efficiency. **Conclusions:** EEG-based evidence suggests that cognitive dysfunction in OCD reflects a dysregulated control system rather than a general reduction in cognitive ability. The disorder appears to involve excessive performance monitoring, abnormal sensory and attentional gating, inefficient inhibition, altered feedback evaluation, and reduced cognitive flexibility. However, the current evidence is limited by heterogeneity in samples, paradigms, EEG methodology, medication status, and statistical approaches. Future studies should use larger, well-characterised samples, standardised EEG/ERP paradigms, direct brain–behaviour analyses, and longitudinal designs to clarify whether electrophysiological abnormalities represent trait markers, state-dependent effects, compensatory mechanisms, or clinically useful predictors of cognitive dysfunction in OCD.

## 1. Introduction

Obsessive–compulsive disorder (OCD) is a disabling psychiatric disorder characterised by intrusive, unwanted thoughts, images, impulses, or doubts, together with repetitive behaviours or mental rituals performed to reduce distress or prevent feared outcomes. Although OCD is traditionally defined by obsessions and compulsions, a growing body of neuropsychological, neuroimaging, and electrophysiological research indicates that the disorder also involves measurable disturbances in cognitive functioning. These cognitive abnormalities are not merely secondary features of anxiety or distress; rather, they may reflect core alterations in the neural systems responsible for monitoring behaviour, inhibiting inappropriate responses, updating action–outcome associations, allocating attention, and evaluating uncertainty. Across clinical and experimental studies, patients with OCD have shown impairments in several cognitive domains, including executive functioning, inhibitory control, cognitive flexibility, planning, working memory, visuospatial memory, decision-making, and performance monitoring [1,2,3,4].

Among these domains, executive dysfunction has received particular attention because it maps closely onto the clinical phenomenology of OCD. Compulsions can be conceptualised as failures to flexibly terminate or update behaviour, whereas obsessions may reflect difficulty suppressing internally generated threat-related thoughts. Meta-analytic evidence suggests that OCD is associated with broad but generally small-to-moderate impairments across executive tasks, with especially consistent abnormalities in response inhibition, set-shifting, planning, and cognitive flexibility [1,3,4]. Experimental studies using tasks such as the stop-signal task, intradimensional/extradimensional (ID/ED) set-shifting task, reversal-learning paradigms, and Tower of London planning tasks have further shown that patients with OCD often require greater cognitive effort to inhibit prepotent responses, adapt to changing contingencies, or organise goal-directed behaviour [5,6,7,8]. Importantly, similar impairments have also been observed in unaffected first-degree relatives of patients with OCD, suggesting that at least some cognitive abnormalities may represent trait-like vulnerability markers rather than only consequences of active symptoms [9,10].

Cognitive dysfunction in OCD is usually interpreted within the broader framework of cortico-striato-thalamo-cortical circuitry. Neurobiological models emphasise abnormal communication between the orbitofrontal cortex, anterior cingulate cortex, dorsolateral prefrontal cortex, basal ganglia, and thalamus, regions involved in action selection, error detection, threat evaluation, cognitive control, and habit formation [11,12,13]. From this perspective, OCD may involve a persistent imbalance between systems that generate warning signals and systems that flexibly resolve uncertainty or terminate defensive actions. Neuropsychological studies support this model by showing that OCD-related cognitive deficits are most evident in tasks requiring adaptive control under conflict, uncertainty, or feedback-dependent updating [7,14,15]. Thus, cognitive dysfunction in OCD is not limited to “poor performance” in a general sense. It appears to involve specific abnormalities in the timing, intensity, and regulation of neural processes that support monitoring, inhibition, learning, and behavioural adjustment.

Electroencephalography, or EEG, is a non-invasive neurophysiological method used to record the brain’s electrical activity from electrodes placed on the scalp [16]. The signal recorded by EEG mainly reflects synchronised postsynaptic activity generated by large populations of cortical pyramidal neurons [17]. In other words, EEG does not directly measure single-neuron firing or action potentials, but rather the summed electrical fields produced when many neurons become active in a coordinated manner [17]. Because these electrical changes occur very rapidly, EEG has excellent temporal resolution, usually at the millisecond level [18]. This makes it especially useful for studying the timing of cognitive processes such as attention, inhibition, conflict monitoring, error detection, memory updating, and stimulus evaluation.

EEG can be divided into several major types depending on the recording context and analytical approach. The first is resting-state EEG, in which brain activity is recorded while the participant is not performing a specific task, usually with eyes closed, eyes open, or both [19]. Resting-state EEG is used to assess spontaneous brain rhythms and general functional organisation of cortical activity. It is commonly analysed in frequency bands such as delta, theta, alpha, beta, and gamma. In psychiatric and cognitive research, resting-state EEG can provide information about cortical arousal, vigilance, inhibitory control networks, attentional readiness, and large-scale functional communication.

A second major type is task-based EEG, in which electrical brain activity is recorded while participants perform a cognitive, emotional, sensory, or motor task. This approach allows researchers to examine how the brain responds during specific mental operations [20].

A particularly important subtype of task-based EEG is the event-related potential, or ERP. ERPs are obtained by averaging EEG activity time-locked to repeated events, such as stimulus presentation, response execution, feedback, or error commission. Averaging reduces background EEG activity and reveals stereotyped voltage deflections associated with specific cognitive operations. ERP components are usually described by their polarity, latency, and scalp distribution.

Cognitive dysfunction in obsessive–compulsive disorder should be examined not only at the behavioural level, but also at the level of its neural and temporal mechanisms. Although neuropsychological studies have repeatedly shown that individuals with OCD may present impairments in executive functioning, cognitive flexibility, response inhibition, planning, working memory, memory confidence, attentional control, and goal-directed regulation, behavioural measures alone provide only a partial account of these abnormalities. Reaction times, accuracy scores, error rates, or neuropsychological test scores indicate whether performance is impaired, but they do not reveal precisely which stage of information processing is disrupted. Similar behavioural outcomes may result from different mechanisms, such as inefficient early sensory encoding, abnormal attentional allocation, excessive conflict monitoring, impaired inhibitory control, delayed stimulus evaluation, reduced working-memory updating, altered error detection, or abnormal post-error adjustment. This distinction is especially important in OCD, because patients may sometimes perform similarly to healthy controls while still showing abnormal neural activity during task performance. In such cases, behavioural performance may be preserved by compensatory recruitment, excessive monitoring, increased effort, or inefficient but successful control strategies. Therefore, the neural correlates of cognitive dysfunction in OCD need to be examined directly if we are to understand not only whether cognition is impaired, but how and when these impairments emerge during information processing.

Electroencephalography is particularly well suited for this purpose because it provides a temporally precise measure of brain activity. Unlike behavioural testing, which captures the final output of cognition, EEG allows researchers to track the unfolding of cognitive operations on a millisecond scale. This is crucial for OCD, where many clinically relevant processes—such as intrusive thought monitoring, doubt, checking, conflict detection, inhibition of compulsive urges, and evaluation of errors—occur rapidly and dynamically. Event-related potentials can separate early perceptual–attentional processing from later cognitive control and evaluative stages, while resting-state and quantitative EEG can provide information about broader alterations in cortical arousal, vigilance, oscillatory activity, and functional connectivity. These electrophysiological measures are therefore not merely descriptive biomarkers; they provide mechanistic information about the cognitive architecture of the disorder.

A review focused on EEG correlates of cognitive dysfunction in OCD is therefore justified because it can clarify which electrophysiological markers are most consistently associated with impaired cognition, which cognitive domains have received the strongest support, and where the evidence remains inconsistent. Such a review can determine whether EEG findings converge on a coherent model of OCD as a disorder of hyperactive performance monitoring, impaired inhibitory control, inefficient attentional allocation, abnormal memory updating, altered cognitive flexibility, or disrupted goal-directed regulation. It can also identify important methodological sources of heterogeneity, including differences in task paradigms, symptom dimensions, medication status, comorbidities, age groups, electrode montages, ERP component definitions, frequency-band analyses, and statistical approaches. This is important because inconsistencies in the literature may reflect not only true clinical heterogeneity, but also differences in how cognitive dysfunction and EEG activity have been operationalised across studies.

## 2. Materials and Methods

### 2.1. Review Design and Reporting Standards

This systematic review was designed to identify and synthesise studies examining electrophysiological correlates of cognitive dysfunction in patients with OCD. The review focused on electroencephalographic and event-related potential evidence, including resting-state EEG, quantitative EEG, spectral and connectivity measures, and ERP components recorded during cognitive or sensory-cognitive paradigms. The review was conducted in accordance with the Preferred Reporting Items for Systematic Reviews and Meta-Analyses guidelines. The protocol was registered in PROSPERO (CRD420261423762).

### 2.2. Search Strategy

A systematic literature search was conducted in PubMed/MEDLINE, Scopus, Web of Science Core Collection, Embase, PsycINFO, and Google Scholar from database inception to 30 May 2026. Additional records were identified through manual screening of reference lists from eligible articles. No restriction was applied to year of publication. The search strategy combined terms related to OCD, electrophysiology, and cognitive functioning. The following Boolean structure was adapted for each database: (“obsessive–compulsive disorder” OR OCD OR obsessive–compulsive) AND (EEG OR electroencephalography OR qEEG OR “quantitative EEG” OR ERP OR “event-related potential” OR “event related potential” OR P300 OR P3 OR N200 OR N2 OR ERN OR “error-related negativity” OR Pe OR FRN OR P50 OR N100 OR N1 OR P200 OR P2 OR CNV OR “contingent negative variation” OR LRP OR “lateralised readiness potential”) AND (cognition OR cognitive OR neuropsychological OR attention OR inhibition OR “response inhibition” OR “error monitoring” OR memory OR “working memory” OR decision-making OR “executive function” OR “cognitive control” OR performance).

### 2.3. Eligibility Criteria

Studies were eligible when they included participants with OCD diagnosed according to recognised DSM or ICD criteria, based on a structured or semi-structured diagnostic interview, or confirmed through a clinical assessment conducted by a qualified mental-health professional. The use of a specific structured interview was not mandatory. Studies relying exclusively on self-reported obsessive–compulsive symptoms without clinical diagnostic confirmation were not classified as clinical OCD studies, although dimensional studies of obsessive–compulsive traits were considered separately where they otherwise met the review criteria. This criterion was applied to reduce diagnostic misclassification between OCD and obsessive–compulsive symptoms occurring in nonclinical populations or other psychiatric disorders. However, we recognise that diagnostic practices and access to validated translated instruments differ across countries and clinical settings.

Studies were included regardless of whether the relationship between electrophysiological measures and cognitive performance was tested through formal statistical correlation, provided that the EEG/ERP paradigm was directly linked to a cognitive process such as attention, inhibition, working memory, error monitoring, feedback processing, sensory gating, conflict monitoring, decision-making, or response preparation.

Exclusion criteria were (1) non-human studies; (2) case reports, conference abstracts, editorials, letters, protocols, dissertations, and non-peer-reviewed material; (3) studies not including patients with OCD; (4) studies using neuroimaging methods without EEG or ERP outcomes; (5) treatment studies in which electrophysiological measures were reported only as treatment-response biomarkers without a cognitive task or cognitive-performance component; and (6) articles for which the full text was unavailable after reasonable retrieval attempts. Reviews and meta-analyses were not included in the synthesis but were screened for additional references.

### 2.4. Study Selection

After duplicate removal, titles and abstracts were screened by two reviewers according to the eligibility criteria. Potentially relevant articles were then assessed in full text. Uncertainties regarding eligibility were resolved by discussion.

### 2.5. Data Extraction

Data were extracted using a standardised extraction form. For each eligible study, the following information was collected: author and year of publication, country, study design, sample size, participant characteristics, diagnostic criteria, OCD symptom severity, medication status, comorbidities, control group characteristics, EEG/ERP paradigm, cognitive task or neuropsychological measure, EEG acquisition parameters, preprocessing procedures, electrophysiological outcomes, behavioural outcomes, statistical approach, and main findings relevant to cognitive dysfunction. When available, associations between electrophysiological measures and cognitive performance, symptom severity, or clinical subdimensions were also extracted.

Electrophysiological outcomes were organised according to modality and component. Resting-state and qEEG studies were categorised according to spectral power, frequency band abnormalities, topography, coherence, connectivity, asymmetry, or other quantitative indices. ERP studies were categorised according to the component examined, including early sensory components, attentional components, conflict-related components, error-monitoring components, feedback-related components, and motor-preparatory components.

### 2.6. Risk-of-Bias and Methodological Quality Assessment

Risk of bias was evaluated using an adapted version of the Risk Of Bias In Non-randomised Studies—of Interventions tool (ROBINS-I). ROBINS-I was selected because the evidence included in this review consisted predominantly of non-randomised comparative studies, and its domain-based structure permitted systematic assessment of the principal threats to validity relevant to clinical EEG and ERP research, including confounding, participant selection, classification of group status, deviations from the intended exposure or study protocol, missing data, outcome measurement, and selection of the reported result. However, because ROBINS-I was originally developed for studies estimating intervention effects, its terminology was adapted to the present review, in which OCD diagnosis, familial status, or level of obsessive–compulsive symptoms constituted the exposure or group-defining characteristic.

For each study, the result most directly relevant to the relationship between electrophysiological activity and cognitive functioning was assessed. Bias due to confounding was considered medication exposure, psychiatric comorbidity, depressive and anxiety symptoms, age, education, intelligence, illness duration, symptom severity, and other variables capable of influencing both EEG measures and cognitive performance. Participant-selection judgements considered recruitment setting, convenience sampling, restrictive eligibility criteria, and exclusions resulting from insufficient task performance, an inadequate number of error trials, or poor EEG quality. Missing-data judgements considered both the amount of missing information and whether EEG or behavioural exclusions could be related to clinical status or cognitive performance. Outcome-measurement judgements considered the objectivity and standardisation of the cognitive and electrophysiological measures, the reliability of ERP estimates, the number of accepted trials, and whether EEG processing or outcome assessment was conducted without knowledge of group status. Selection-of-reported-result judgements considered the number of components, electrodes, frequency bands, task conditions, subgroup analyses, and correlations examined, together with the use of multiplicity correction, preregistration, or a prespecified analytical protocol.

Each domain was assigned a single judgement of low, moderate, serious, or critical risk of bias, or no information where the available report was insufficient. The overall judgement followed the most severe domain-level judgement: a study was rated as low risk only when all domains were low risk, moderate when no domain exceeded moderate risk, serious when at least one domain was at serious risk but none was critical, and critical when at least one domain was rated critical. Risk-of-bias judgements were used to determine the weight given to each study in the narrative synthesis rather than as an automatic criterion for study exclusion.

### 2.7. Data Synthesis

Because the included studies were expected to differ substantially in participant characteristics, medication status, EEG/ERP paradigms, cognitive tasks, electrophysiological outcomes, and statistical approaches, a quantitative meta-analysis was not performed. Instead, findings were synthesised narratively and mechanistically. Studies were first grouped according to electrophysiological modality, including conventional EEG/qEEG and ERP evidence. ERP studies were then organised by component, such as P50, N1/N100, P2/P200, N2/N200, N450, P3/P300, P600, ERN, Pe, FRN, CNV, Pc, and LRP. Within each component or EEG domain, findings were interpreted in relation to the cognitive process most strongly represented by the paradigm, including sensory gating, early perceptual processing, attentional allocation, inhibitory control, conflict monitoring, working memory, feedback learning, error monitoring, and response preparation.

The synthesis prioritised studies that directly linked electrophysiological abnormalities to cognitive performance or task-related dysfunction. However, studies that examined electrophysiological activity during cognitive paradigms without formal brain–behaviour correlations were also considered when they provided mechanistic information about cognitive dysfunction in OCD. The final interpretation emphasised convergent findings across tasks, components, and study years, while also considering inconsistencies related to sample characteristics, medication exposure, comorbidity, task demands, and methodological variability.

## 3. Results

**Figure 1:**
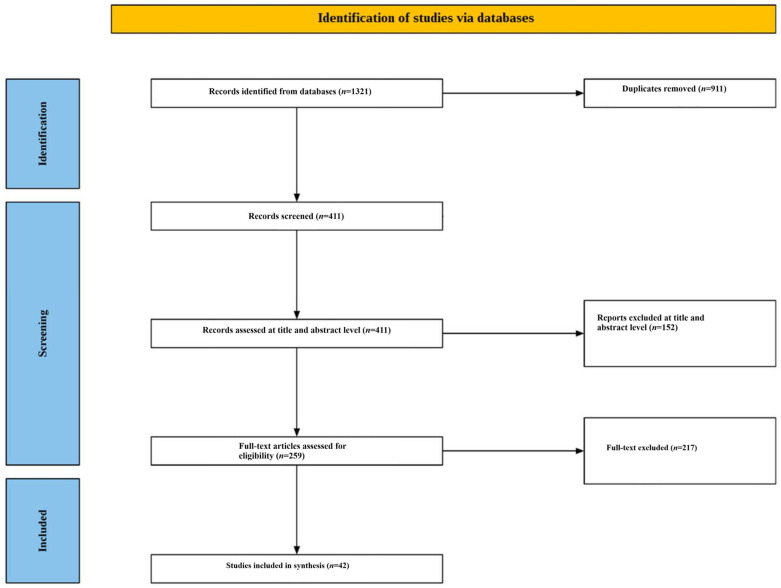
Flowchart depicting the study-selection process.

Figure 1 provides a summary of the screening process. The literature search identified 1321 records. Prisma checklist is presented in Supplementary File S1. After 910 duplicates had been removed, 411 records remained for title and abstract screening. A total of 411 records underwent title and abstract screening. At this stage, 152 studies were removed because they did not meet the inclusion criteria. The full texts of 259 reports were assessed for eligibility. At this stage, 217 studies were excluded because they did not measure the relationship between EEG findings and cognitive performance in individuals with OCD. The principal reasons were absence of an eligible relationship between electrophysiological measures and cognitive functioning (*n* = 172), absence of a clinical or dimensional OCD sample meeting the eligibility criteria (*n* = 17), insufficiently established or insufficiently reported OCD diagnosis (*n* = 5), treatment-focused electrophysiological outcomes without a relevant cognitive component (*n* = 10), ineligible publication type (*n* = 7), and unavailable full text or insufficient data for assessment (*n* = 5). These categories were assigned according to the principal reason for exclusion and sum to the total number of full-text exclusions. Ultimately, 42 studies met the inclusion criteria [21,22,23,24,25,26,27,28,29,30,31,32,33,34,35,36,37,38,39,40,41,42,43,44,45,46,47,48,49,50,51,52,53,54,55,56,57,58,59,60,61,62]. The included ERP studies are presented in Table 1, and the EEG studies are presented in Table 2.

### 3.1. Participant Characteristics

Across the included studies, participants were predominantly individuals with clinically diagnosed obsessive–compulsive disorder and demographically matched healthy control groups, although several studies also included psychiatric comparison groups, unaffected relatives, or subclinical obsessive–compulsive symptom groups. The clinical OCD samples varied considerably in size, ranging from small experimental samples of approximately 11–16 patients to large paediatric cohorts including more than 100 OCD participants. Most adult studies enrolled between 14 and 33 patients with OCD, whereas several paediatric or adolescent investigations used substantially larger samples, including 80, 99, and 105 youths with lifetime or current OCD diagnoses. Healthy control groups were usually matched to OCD groups for age and sex, and in many studies also for education, handedness, IQ, or verbal intelligence. This matching was important because the reviewed studies examined EEG or ERP markers in relation to cognitive performance, where demographic factors such as age, education, and general intellectual ability can substantially influence both electrophysiological responses and task performance.

Most adult OCD samples consisted of young or middle-aged adults, generally with mean ages in the twenties to thirties. For example, several studies included adult OCD groups with mean ages around 27–35 years, while others recruited somewhat older adult samples, including one study in which OCD patients and relatives were in their forties. Paediatric and adolescent studies covered a broader developmental range, typically including children and adolescents aged approximately 8–18 years. These developmental studies were particularly important because they allowed examination of whether electrophysiological markers of cognitive dysfunction, especially error monitoring and inhibitory control, are already present early in the course of OCD. Some paediatric samples included only youths with current OCD, whereas others included participants with lifetime OCD, including individuals with past OCD and minimal current symptoms. This distinction is relevant because electrophysiological abnormalities in such samples may reflect trait-like vulnerability markers rather than only state-dependent effects of current symptom severity.

Sex distribution was generally balanced or explicitly matched between OCD and control groups, although some samples showed modest male or female predominance. Several adult studies included approximately equal numbers of men and women, while some paediatric samples contained more girls than boys. In most studies, the control group closely paralleled the OCD group in sex distribution. Handedness was also controlled in many studies, with several investigations including only right-handed participants, whereas others matched OCD and control groups on handedness. This was particularly relevant for EEG and ERP studies because hemispheric asymmetry, motor-response lateralisation, and source-localisation analyses can be affected by handedness.

Diagnosis was usually established using structured or semi-structured clinical interviews based on DSM or ICD criteria, including instruments such as the Structured Clinical Interview for DSM-IV, K-SADS, MINI, or structured psychiatric assessments conducted by trained clinicians or psychiatrists. OCD severity was most commonly measured with the Yale–Brown Obsessive Compulsive Scale in adult samples and the Children’s Yale–Brown Obsessive Compulsive Scale in paediatric samples. Across studies, OCD severity was generally in the clinically meaningful range. Adult samples often showed mean Y-BOCS scores in the mild-to-moderate or moderate range, such as approximately 18–26, whereas paediatric samples reported current or lifetime CY-BOCS scores indicating clinically relevant obsessive–compulsive symptoms. Several studies also provided illness-duration or age-at-onset information, with adult samples often showing illness durations of several years and paediatric samples reporting early onset, frequently around middle childhood. This indicates that many participants had established OCD rather than very recent or transient symptoms.

Medication status varied substantially across the reviewed studies. Some investigations deliberately recruited medication-free or drug-naïve patients, or required medication washout periods before EEG recording. These designs reduced the likelihood that electrophysiological differences were driven by psychotropic medication effects. Other studies included medicated patients, most often individuals receiving selective serotonin reuptake inhibitors or other antidepressants, sometimes under stable-dose conditions. In several paediatric studies, stable SSRI treatment was permitted, but other psychotropic medications were excluded. A few adult studies included patients taking antidepressants, anxiolytics, antipsychotics, or combined treatments; in these studies, medication status was usually acknowledged as a potential confound, and some authors tested whether medication dose was associated with cognitive or electrophysiological outcomes. Overall, the literature therefore contains both relatively “clean” medication-free samples and more naturalistic samples reflecting treated clinical OCD.

Comorbidity was handled differently across studies. Some studies used strict exclusion criteria, excluding major depression, anxiety disorders, tic disorders, ADHD, psychotic disorders, bipolar disorder, substance abuse, neurological illness, head injury, intellectual disability, or major medical illness. Other studies allowed selected comorbidities, particularly depressive or anxiety symptoms, provided that OCD was the primary diagnosis. Several paediatric studies excluded ADHD, tic disorders, current major depression, psychosis, bipolar disorder, substance-related disorders, anorexia nervosa, intellectual disability, significant head injury, and chronic neurological disease, thereby attempting to isolate OCD-related electrophysiological effects. In contrast, some broader paediatric cohorts explicitly examined tic-related versus non-tic-related OCD or OCD with and without major depressive disorder. These designs are useful because they show whether EEG markers of cognitive dysfunction are specific to OCD or are influenced by clinically relevant subtypes and comorbid symptom dimensions.

Several studies extended beyond a simple OCD-versus-control design by including psychiatric or familial comparison groups. Some adult studies compared OCD patients with schizophrenia, panic disorder, obsessive–compulsive personality disorder, or high health anxiety groups. These comparison groups helped determine whether EEG abnormalities associated with cognitive performance were specific to OCD or reflected broader mechanisms related to anxiety, threat processing, compulsivity, psychosis, or decision-making dysfunction. Other studies included unaffected siblings or first-degree relatives of OCD patients. These familial-risk samples were particularly important for testing whether electrophysiological or cognitive abnormalities may function as candidate endophenotypes, because abnormalities shared by OCD patients and unaffected relatives may indicate inherited vulnerability rather than consequences of active symptoms.

A smaller subset of studies did not examine clinically diagnosed OCD patients but instead used dimensional or subclinical obsessive–compulsive symptom designs. These studies recruited university students or community participants with high versus low obsessive–compulsive symptom scores, often based on OCI-R or Padua Inventory cutoffs. Such samples should not be interpreted as clinical OCD cohorts. However, they are relevant to the broader review because they tested whether obsessive–compulsive traits are associated with electrophysiological markers of cognitive processes such as feedback learning, error monitoring, decision-making, and perceptual evidence accumulation. These studies generally involved young adults and often excluded neurological illness, head trauma, psychoactive medication use, or major psychiatric disorders, although at least one dimensional study did not screen participants for current or past psychiatric diagnoses. Therefore, findings from these studies should be presented as evidence concerning obsessive–compulsive symptom dimensions rather than categorical OCD.

### 3.2. EEG Paradigms

The included studies used a broad range of EEG and ERP paradigms to examine the relationship between electrophysiological activity and cognitive performance in obsessive–compulsive disorder. Most studies were task-based ERP investigations rather than resting-state EEG studies. These paradigms were selected to probe cognitive domains repeatedly implicated in OCD, including attentional allocation, stimulus evaluation, response inhibition, conflict monitoring, error detection, post-error adjustment, feedback learning, reversal learning, decision-making, working memory, episodic memory, sensory gating, perceptual evidence accumulation, and cognitive flexibility. Across studies, EEG was therefore not used as a single uniform method, but as a set of temporally precise paradigms designed to isolate different stages of cognitive processing.

A major group of studies used auditory oddball paradigms to examine P300-related attentional and stimulus-evaluation processes. In these tasks, participants were usually exposed to frequent standard tones and infrequent target tones and were required either to count or respond to the rare target stimuli. The oddball design was used to elicit P300 amplitude, latency, or duration, which were interpreted as indices of attentional resource allocation, stimulus evaluation, context updating, and target detection. Some studies combined auditory oddball ERP recording with independent neuropsychological tests, such as the Stroop Test, Trail-Making Test, verbal fluency, design fluency, or broader executive-function batteries. Other studies used behavioural performance within the oddball task itself, including reaction time, target-detection accuracy, or intra-individual response-time variability. In paediatric OCD, oddball paradigms were also used to test whether reduced P300 amplitude was associated with sustained-attention lapses or altered reaction-time variability. Thus, oddball paradigms provided one of the clearest methodological links between EEG markers of attentional processing and cognitive performance.

Another important group of paradigms focused on response inhibition. Several studies used Go/NoGo tasks, in which participants had to respond to frequent Go stimuli and withhold responses to NoGo stimuli. These paradigms were primarily used to examine N2 and P3 components, with NoGo-N2 interpreted as a marker of inhibitory control or conflict monitoring and NoGo-P3 as a later marker of inhibition implementation or stimulus evaluation under inhibitory demands. Some Go/NoGo studies used equal Go and NoGo probabilities to reduce confounding effects of stimulus rarity, whereas others embedded the Go/NoGo task within more complex affective or error-processing designs. In these studies, behavioural indices such as reaction time, omission errors, and commission errors were used as cognitive-performance measures. However, in several cases the electrophysiological abnormalities were more pronounced than the behavioural differences, suggesting that EEG may detect altered inhibitory processing even when overt performance appears relatively preserved.

Stop-signal tasks were also used to examine motor inhibition more directly. In these paradigms, participants prepared or initiated a speeded response on Go trials but had to cancel that response when a stop signal appeared. The key behavioural measure was usually the stop-signal reaction time, reflecting the estimated latency of the internal stopping process. EEG was time-locked to stop-signal presentation, and the main components were Stop-N2 and Stop-P3. These components allowed the studies to distinguish earlier conflict or stopping-related processing from later monitoring of successful or failed inhibition. Compared with simple Go/NoGo tasks, stop-signal paradigms were particularly suitable for testing whether OCD involves impaired cancellation of an already prepared response rather than only difficulty withholding a response before it is initiated.

Several studies used conflict-monitoring and error-monitoring paradigms, especially flanker tasks, anti-saccade tasks, and response-conflict tasks. These paradigms were designed to elicit response-locked components such as the error-related negativity, correct-response negativity, Pe, and sometimes Pc. In flanker-type tasks, participants responded to a central stimulus while ignoring distracting flankers, creating conflict between correct and incorrect response tendencies. In anti-saccade tasks, participants had to suppress a reflexive eye movement toward a stimulus and instead generate a voluntary saccade in the opposite direction. These designs allowed researchers to examine both error detection and post-error behavioural adjustment. The ERN was typically interpreted as an index of early performance monitoring or error detection, whereas the Pe/Pc complex reflected later conscious error evaluation, error awareness, or internal feedback about whether an action was correct. Some studies extended this logic by examining whether abnormal error-related activity predicted post-error slowing, self-correction, affective evaluation of errors, or broader clinical and cognitive outcomes.

Task-switching paradigms were used to examine cognitive flexibility and the ability to shift between mental sets. One important design was a backward-inhibition task-switching paradigm, in which participants alternated between different task rules and sometimes had to return to a recently abandoned rule. This paradigm was especially relevant to OCD because it tested whether repetitive or recently used task sets are unusually easy or difficult to reactivate. EEG was time-locked to cues and targets, and analyses focused on components such as P1, N1, N2, and P3. These studies therefore moved beyond simple inhibition and examined the dynamic interaction between task-set suppression, reactivation, and cognitive flexibility. The combination of behavioural switching costs and ERP measures made it possible to test whether altered flexibility in OCD reflects early sensory-attentional gating, conflict monitoring, or later response-selection processes.

Emotional Stroop paradigms were used to investigate interference control in the presence of emotionally salient or symptom-relevant stimuli. In these tasks, participants were required to name or respond to the colour of words while ignoring their emotional meaning. Some studies compared negative, positive, neutral, or threat-related words, whereas others distinguished autogenous and reactive OCD subtypes using personally or clinically relevant stimulus categories. These paradigms were designed to assess whether OCD-related cognitive dysfunction involves difficulty suppressing irrelevant emotional meaning. EEG analyses focused on components such as N450, P3, and sometimes earlier sensory-attentional components. N450 was particularly relevant as an index of conflict detection and interference processing during Stroop-like tasks, whereas P3 reflected later attentional allocation and elaborative processing of emotional or threat-related information.

Working-memory paradigms were less common but conceptually important. One study used a digit-span working-memory task in which warning tones indicated whether participants would later recall digits in forward or reverse order. EEG was recorded during the preparatory interval between the warning cue and the digit sequence, and the P600 component was analysed as a marker of late anticipatory information processing. This design differed from many other paradigms because the ERP was not measured during the memory response itself, but during preparation for upcoming memory encoding and recall. The paradigm therefore tested whether OCD is associated with abnormal preparatory neural activity during working-memory engagement, rather than only impaired performance after the memory load is presented.

Memory-retrieval paradigms were also used to examine episodic and source-memory processes. In these studies, participants encoded verbal stimuli and later performed recognition or source-memory judgments. For example, participants might hear words spoken by male or female voices and later determine whether visually presented words were old or new, or whether they had originally been spoken by a male or female speaker. EEG was time-locked to recognition-test stimuli, and ERP old/new effects were analysed as markers of successful memory retrieval. These paradigms were useful because they separated simpler item recognition from more demanding source-memory retrieval, a distinction relevant to OCD because patients may show preserved recognition but impaired contextual memory or reduced confidence in memory decisions.

Sensory-gating paradigms formed another distinct methodological category. The most common was the paired-click P50 paradigm, in which a conditioning auditory click was followed by a second test click. The degree to which the P50 response to the second click was suppressed relative to the first click was used as an index of early auditory sensory gating. Some studies also used prepulse inhibition paradigms, in which a weak prepulse preceded a target or startle stimulus at different intervals. These paradigms were relevant because OCD is often conceptualised as involving impaired inhibition, and sensory-gating measures test whether such inhibitory abnormalities are already present at very early stages of sensory processing. In some designs, sensory gating was combined with active auditory discrimination tasks, allowing comparison between early automatic gating indices such as P50 or N1 suppression and later cognitive indices such as P3 differentiation between targets and non-targets.

A large subset of studies examined feedback processing, reinforcement learning, and decision-making. These paradigms included deterministic reversal-learning tasks, probabilistic reinforcement-learning tasks, observational- versus active-learning tasks, and modified Iowa Gambling Tasks. In reversal-learning paradigms, participants learned which stimulus was correct, then had to adapt when reward contingencies changed without warning. In probabilistic-learning tasks, participants learned through trial and error which stimuli were more likely to produce rewards or punishments. In modified Iowa Gambling Tasks, participants repeatedly chose between advantageous and disadvantageous options and had to learn from feedback which option produced better long-term outcomes. EEG was typically time-locked to feedback presentation, and the main components were feedback-related negativity and feedback-related P300. These paradigms were especially useful for examining whether OCD-related cognitive dysfunction involves abnormal use of external feedback, impaired reward learning, exaggerated punishment monitoring, or reduced flexibility after negative outcomes. Some studies further strengthened this approach by using computational reinforcement-learning models to relate trial-level prediction errors or subjective value estimates to ERP amplitudes.

Decision-making paradigms were not limited to reward learning. Some studies used perceptual decision-making tasks, especially random-dot motion tasks, to examine how participants accumulate sensory evidence over time. In these paradigms, participants judged the direction of coherent dot motion under different difficulty levels. Behavioural performance was modelled using drift-diffusion parameters, especially drift rate, which reflects the efficiency of evidence accumulation. EEG analyses focused on oscillatory activity, particularly beta- and gamma-band dynamics, rather than only traditional ERP components. This approach was important because it linked obsessive–compulsive symptoms to a computationally defined cognitive mechanism: inefficient integration of perceptual evidence during decision-making.

Resting-state EEG and quantitative EEG studies formed a smaller but important part of the literature. These studies recorded EEG during eyes-closed or alternating eyes-open and eyes-closed resting conditions and analysed spectral power across standard frequency bands such as delta, theta, alpha, beta, and sometimes gamma. Some studies examined absolute and relative power, whereas others focused on hemispheric asymmetry, frontal alpha activity, or source-localised oscillatory activity. Resting EEG paradigms were usually paired with neuropsychological tests administered separately, such as visuospatial memory, verbal memory, or the Rey–Osterrieth Complex Figure Test. These designs allowed researchers to test whether baseline cortical activity or hemispheric imbalance was associated with cognitive dysfunction outside a specific task context. In contrast to ERP paradigms, which isolated rapid stimulus- or response-related processes, resting-state EEG paradigms examined more tonic electrophysiological characteristics that might reflect trait-like neural organisation in OCD.

Several studies also used time–frequency approaches during cognitive tasks. Instead of analysing only averaged ERP waveforms, these studies examined oscillatory power and phase consistency in frequency bands such as theta, beta, and gamma. Mid-frontal theta activity was particularly relevant in response-monitoring tasks, because theta power and phase coherence are closely linked to cognitive control, error monitoring, and adaptive performance regulation. In paediatric OCD, response-locked theta power was examined during cognitive-control tasks to determine whether enhanced error-related activity reflected stronger oscillatory power, greater phase locking, or both. This distinction was methodologically important because an ERP abnormality can arise from increased oscillatory magnitude, increased phase alignment across trials, or a combination of both. Time–frequency paradigms therefore provided a more mechanistic account of the neural processes underlying conventional ERP abnormalities.

Across studies, EEG acquisition methods varied widely. Low-density recordings used approximately 15–25 electrodes, whereas more recent studies often used 32-, 60-, 61-, 64-, or even higher-density montages. Most studies followed the international 10–20 or extended 10–20 system, used mastoid or average references, monitored ocular artefacts with electrooculography, and rejected or corrected artefact-contaminated trials. ERP studies typically segmented data around stimulus onset, feedback onset, or response execution, depending on the cognitive process of interest. Some studies supplemented scalp-level EEG with source-localisation techniques such as sLORETA or equivalent current dipole modelling, allowing approximate localisation of generators in regions such as the anterior cingulate cortex, inferior frontal gyrus, frontal cortex, parietal cortex, or temporo-parietal regions. Other studies combined EEG with fMRI or DTI, linking electrophysiological markers of error processing with broader neural-network measures.

**Figure 2:**
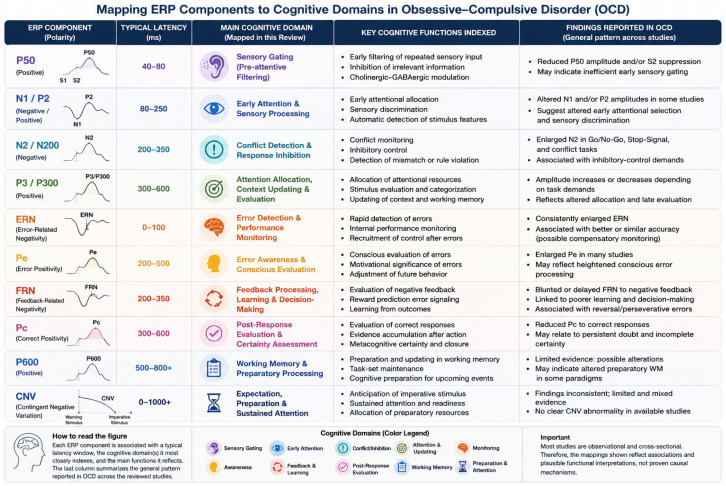
This figure summarises the principal event-related potential (ERP) components reviewed in this manuscript and illustrates the cognitive domains with which they are most commonly associated. Early components such as P50, N1/N100, and P2/P200 are mainly linked to sensory gating and early attentional processing; N2/N200 and N450 to conflict monitoring and inhibitory control; P3/P300 to attentional allocation, stimulus evaluation, and context updating; ERN, Pe, and Pc to performance monitoring and post-response evaluation; FRN to feedback processing and reinforcement learning; P600 to working-memory-related preparatory processing; and CNV and LRP to anticipatory attention and motor preparation, respectively. The figure is intended as a conceptual summary of the reviewed literature and reflects interpretive mappings derived from the included studies rather than definitive one-to-one functional assignments.

To facilitate interpretation of the reviewed ERP findings, Figure 2 provides a schematic overview of the principal ERP components discussed in this review and maps them onto the cognitive domains with which they are most commonly associated, including sensory gating, attentional allocation, inhibitory control, conflict monitoring, error processing, feedback learning, and working-memory-related preparatory processing.

### 3.3. P3/P300

In auditory oddball paradigms, several studies pointed to abnormalities in P300-related target evaluation, although the direction and cognitive meaning of these effects varied. One study found that OCD patients had significantly shorter P300 duration than healthy controls, while P300 latency and amplitude were not significantly altered [1]. This finding argues against a generalised reduction in P300 amplitude or a broad delay in stimulus evaluation, but suggests that the temporal window of P300-related processing may be shortened in OCD. Importantly, this electrophysiological difference was not accompanied by clear group-level impairment on executive neuropsychological tasks such as the Stroop Test, Trail-Making Test, Design Fluency Test, or Controlled Word Association Test [21]. Nevertheless, within the OCD group, lower posterior P300 amplitudes were associated with longer Stroop interference duration, indicating that weaker P300 responses were related to poorer efficiency when automatic responses had to be inhibited and selective attention had to be maintained [21]. Thus, even when behavioural performance is not globally impaired, P300 measures may reveal individual differences in interference control. Other auditory oddball studies provided stronger evidence for reduced P300 amplitude. In adults with OCD, auditory P300 amplitude was significantly reduced across scalp sites, whereas latency was preserved [27]. The cognitive relevance of this reduction was supported by exploratory correlations showing that smaller frontal P300 amplitudes were associated with slower Trail-Making Test Part B performance, particularly at frontal electrodes [27]. However, the correlations did not survive Bonferroni correction, and OCD patients were not significantly impaired on TMT-B relative to controls, so this association should be treated as suggestive rather than definitive [27]. A paediatric drug-naïve OCD study also found reduced P300 amplitude, especially at frontal and central electrodes, with no significant latency differences [29]. However, P300 measures were not correlated with ex-Gaussian reaction-time parameters, including indices of attentional lapses or response variability [29]. Therefore, reduced P300 amplitude may occur in paediatric OCD, but it does not necessarily explain trial-by-trial attentional variability or symptom severity. The strongest evidence for a cognitive and vulnerability-related interpretation of P300 came from a study including OCD patients, their unaffected siblings, and healthy controls [31]. In this study, P300 amplitude was lowest in OCD patients, intermediate in siblings, and highest in controls, suggesting a possible familial or endophenotypic pattern. Both patients and siblings also showed shorter P300 latencies than controls [31]. Importantly, lower P300 amplitude was associated with slower TMT-B performance in both OCD patients and unaffected siblings, linking reduced P300 activity to poorer executive-attentional efficiency, cognitive flexibility, and processing speed [31]. The same study also reported broader neuropsychological deficits in patients, and milder but partly overlapping impairments in siblings, especially in executive and visuospatial domains [31]. P3/P300 findings from inhibition tasks were more mixed. In a visual Go/NoGo task, OCD patients did not differ from controls in reaction time, omission errors, or commission errors, and NoGo-P3 amplitude was not significantly reduced [22]. However, OCD patients showed longer P3 latency in the NoGo condition than in the Go condition, a pattern not observed in controls [22]. In a stop-signal task, OCD patients showed impaired motor inhibition, reflected by longer stop-signal reaction times, but Stop-P3 amplitude and latency did not differ from controls [23]. By contrast, another stop-signal study comparing autogenous and reactive OCD found reduced Stop-P3 amplitude during successful inhibition in both OCD subtypes, together with longer stop-signal reaction times [54]. However, Stop-P3 latency was not significantly correlated with SSRT, so the ERP–behaviour association was present at the group level but not clearly demonstrated at the individual level [54]. P3/P300 also appeared relevant to cognitive flexibility and feedback-guided learning. In a reversal learning task, OCD patients made more reversal errors and exploration errors, indicating difficulty abandoning previously rewarded responses and using negative feedback to guide new choices [30]. Feedback-related P300 modulation differed between patients and controls. However, this group effect disappeared after behavioural errors were included as a covariate, and P300 modulation was significantly related to error frequency [30]. Thus, feedback-related P300 in this task appeared to reflect performance-dependent outcome evaluation rather than a primary OCD-specific abnormality. In adolescents performing a task-switching paradigm, OCD participants did not show the expected impairment in cognitive flexibility; instead, they showed a smaller backward-inhibition cost than controls [24]. The main ERP effect concerned earlier P1 modulation, but P3 also showed a group-by-condition interaction: controls had smaller P3 amplitudes in backward-inhibition trials than baseline trials, whereas adolescents with OCD did not show this difference [24].

Several findings indicate that P3/P300 abnormalities may become especially relevant when task performance depends on distinguishing targets from non-targets, suppressing threat meaning, or managing personally salient interference. In an active auditory discrimination task combined with prepulse inhibition, OCD patients made more omission errors, responded more slowly, and adopted a more conservative response style [47]. Early sensory gating measures were not significantly related to task performance, but poorer P3 differentiation between target and non-target stimuli, especially in the 500 ms condition, was associated with more omission errors, lower perceptual sensitivity, and altered response criterion [47]. This suggests that later P3-related target evaluation may be more closely tied to cognitive performance than early sensory gating. In emotional Stroop paradigms, P3/P300 findings also pointed to altered late processing of salient material. Autogenous OCD patients showed slower interference-control performance and larger P3b amplitudes than reactive OCD patients and controls across emotional and neutral stimuli [53]. In another emotional Stroop study, individuals who showed behavioural interference had larger P3 amplitudes to threat words than neutral words, whereas those without interference showed the opposite pattern [55]. Because part of the OCD group belonged to the interference subgroup, this suggests that in some patients, personally threatening material may consume late evaluative resources and disrupt task-focused performance [55].

Finally, repetition-priming findings suggest that some P3 abnormalities in OCD may reflect broader anxiety-related processing rather than OCD-specific dysfunction. In a Go/NoGo repetition-priming study, both OCD and panic-disorder patients showed an altered P3 topography, with a reduced parietal-greater-than-frontal distribution, and both clinical groups had longer P3 latencies to Go stimuli than healthy controls [34]. This indicates delayed or atypical late stimulus evaluation, but because the same pattern appeared in panic disorder, it cannot be considered specific to OCD. Instead, it may reflect a transdiagnostic alteration in late attentional updating or evaluative processing under conditions of repeated stimulation [54].

### 3.4. P2/P200

In study [31], P200 was examined during an auditory oddball paradigm in patients with OCD, their unaffected siblings, and healthy controls. The broader cognitive profile showed that OCD patients were the most impaired across several domains, including executive function, selective attention, interference control, processing speed, cognitive flexibility, verbal fluency, working memory, and visuospatial integration. Healthy siblings showed milder but partly similar impairments, suggesting that some cognitive abnormalities may extend beyond the clinical state of OCD. In the ERP results, P200 amplitude did not show a straightforward significant group difference in the main ANOVA. However, the numerical pattern was notable, with the highest P200 amplitude in siblings, intermediate values in OCD patients, and the lowest values in controls. This pattern became more important in the regression analyses, where higher P200 amplitude helped distinguish the combined patient/sibling group from controls. Higher P200 amplitude also contributed to models differentiating siblings from controls, alongside reduced P300 amplitude and lower Block Design Test performance. Thus, although P200 was not directly correlated with a specific neuropsychological measure, elevated P200 amplitude appeared in models that combined electrophysiological and cognitive markers of OCD-related familial vulnerability. A different pattern emerged in the repetition-priming study [54], where P2 was measured during repeated visual Go stimuli in OCD, panic disorder, and healthy control groups. Behaviourally, all groups showed faster reaction times with stimulus repetition, but the facilitation effect was exaggerated in the clinical groups and was especially evident in the OCD non-washer/checker subgroup. Electrophysiologically, the OCD group showed reduced P2 amplitude to Go targets compared with healthy controls. Subgroup analyses indicated that this reduction was driven mainly by non-washing/checking OCD patients, who also showed the clearest behavioural evidence of exaggerated repetition priming.

Importantly, the available evidence does not show a strong direct relationship between P2/P200 and cognitive performance. In study [31], the clearest ERP–cognition correlation involved P300 amplitude and Trail-Making Test B performance, not P200. P200 contributed to regression models predicting patient/sibling status, but this should be interpreted as an indirect association with the broader cognitive-endophenotype profile rather than proof that P200 amplitude explains individual differences in executive performance. Similarly, in study [54], reduced P2 amplitude in OCD non-washers/checkers paralleled exaggerated reaction-time facilitation, but the study did not report a direct P2–behaviour correlation establishing that smaller P2 amplitudes predicted the magnitude of priming.

### 3.5. N1/N100

In the adolescent task-switching study [24], N1 was examined in the context of cognitive flexibility using an adapted backward-inhibition paradigm. This task tested the ability to switch between mental sets and to return to a recently abandoned task. Behaviourally, adolescents with OCD showed a smaller backward-inhibition cost than healthy controls, suggesting that they were less impaired when reactivating a previously used, repetitive task set. This result was interpreted as potentially relevant to the repetitive and perseverative cognitive style characteristic of OCD. However, this behavioural difference was not explained by N1 activity. Cue-locked P1, N1, and P3 did not show the critical group-by-condition interaction, indicating that the relevant group difference was not already present during initial cue processing. Similarly, target-related N1 amplitudes at posterior electrodes and N1 activity at CPz did not show effects corresponding to the behavioural findings. Instead, the main ERP abnormality appeared in the target-related P1 range, where OCD patients showed stronger backward-inhibition-related modulation. This was interpreted as enhanced early inhibitory gating, possibly involving right inferior frontal inhibitory control. Thus, although the study provided evidence for altered cognitive flexibility in OCD, N1 did not appear to be the electrophysiological mechanism underlying this alteration [24].

A somewhat stronger but still indirect role of N100 was observed in the family/endophenotype study [31]. This study used an auditory oddball paradigm and examined N100 together with P200, N200, and P300, while also assessing a broad range of neuropsychological functions. N100 latency differed between groups: healthy siblings of OCD patients showed the shortest N100 latencies, OCD patients showed intermediate latencies, and healthy controls showed the longest latencies. The direct ERP–cognition association emphasised in the study involved P300 rather than N100: lower P300 amplitude correlated with slower Trail-Making Test B performance in both OCD patients and siblings. Regression models also highlighted reduced P300 amplitude, increased P200 amplitude, and poorer visuospatial/executive performance as possible endophenotypic markers, rather than N100. Therefore, although N100 latency was abnormal, the study did not establish N100 as a direct marker of cognitive dysfunction. Its relevance was mainly as an indicator of altered early auditory processing within OCD families, whereas the stronger link with cognitive performance was found for later components [31].

The prepulse-inhibition ERP study [47] also suggested that N1 is sensitive to early sensory modulation, but not specifically to OCD-related cognitive dysfunction. In this active auditory discrimination task, participants heard a brief prepulse followed either 100 ms or 500 ms later by a target or non-target tone, and they had to respond to the target tone. N1 showed a clear prepulse-inhibition effect across all groups: N1 amplitudes were smaller when the tone followed the prepulse after 100 ms than after 500 ms. This indicates that the prepulse reduced early cortical processing of the auditory stimulus. However, this N1 gating effect was not specific to OCD, because it was present in healthy controls, OCD patients, and schizophrenia patients. This contrasted with other sensory or startle-related measures, such as P50 and EMG, which showed more pronounced abnormalities in patient groups. Importantly, N1-PPI was not significantly related to discrimination performance. Although physiological and behavioural PPI measures explained some variance in omission errors, perceptual sensitivity, and response criterion, the N1-PPI difference itself did not predict these outcomes. Instead, behavioural reaction-time PPI and later target-related P3 differentiation were more clearly associated with task performance. Thus, N1 was sensitive to sensory gating manipulations, but it did not function as a disorder-specific or performance-linked marker of cognitive dysfunction in OCD [47].

Visual emotional-processing studies provide somewhat stronger evidence that N1/N100 abnormalities may emerge when stimuli are affectively or personally salient. In the emotional Stroop study comparing autogenous and reactive OCD subtypes [53], N1 effects were limited but suggested that emotional information can modulate very early processing. Across participants, negative words elicited shorter N1 latency than neutral words, indicating faster early discrimination of negatively valenced material. However, this was not a broad OCD-specific N1 abnormality, and the main subtype-related ERP differences appeared later, particularly in P2, P3b, and N450. Nevertheless, the N1 finding is relevant because it suggests that emotional material may influence perceptual–attentional processing before later stages of conflict monitoring and interference control are engaged. In autogenous OCD, this early sensitivity occurred alongside slowed colour naming and later ERP abnormalities during emotional Stroop performance.

The repetition-priming Go/NoGo study [54] provided clearer evidence of abnormal N1/N100 modulation in OCD. At baseline, both OCD and panic-disorder patients showed larger N1 amplitudes than healthy controls, suggesting enhanced early discrimination or heightened attentional processing of visual stimuli. During repeated Go stimuli, all groups showed repetition-related N1 amplitude reduction, consistent with neural adaptation or reduced sensory-processing demands for repeated stimuli. However, OCD patients differed from controls in the latency pattern. Healthy controls showed the expected shortening of N1 latency with repetition, suggesting more efficient processing of repeated stimuli over time. In contrast, OCD patients showed a tendency toward increasing N1 latency across repeated stimuli, and the OCD-versus-control contrast was significant.

The emotional Stroop study using personally threatening words [55] further supports the idea that N1/N100 abnormalities in OCD are most evident when stimuli are individually meaningful. In this study, OCD patients showed longer N1 latency to personally threatening words than to neutral words, relative to panic-disorder patients and healthy controls. This N1 effect followed an earlier P1 enhancement to threat words in OCD, suggesting a two-stage pattern of abnormal early processing. First, personally threatening material captured enhanced early visual attention, as reflected by P1. Then, at the N1 stage, the same material was processed more slowly or effortfully. Importantly, these ERP effects disappeared for low-threat words, indicating that the abnormality was specific to personally meaningful threat rather than to general negative emotionality [55].

### 3.6. N2/N200

The available findings suggest that the N2/N200 component is a potentially sensitive, but highly task-dependent, marker of altered cognitive control in OCD. Across the reviewed studies, N2/N200 abnormalities were most consistently observed in paradigms requiring response inhibition, conflict monitoring, or adaptive control, but their direction and functional meaning varied depending on the specific cognitive operation elicited by the task. In a Go/NoGo paradigm, patients with OCD did not differ from healthy controls in reaction times, omission errors, or commission errors, indicating preserved overt task performance. However, despite this absence of behavioural impairment, the ERP data revealed clear abnormalities at the earlier inhibitory-processing stage. Both groups showed the expected enhancement of N2 amplitude during NoGo relative to Go trials, consistent with the involvement of N2 in inhibition or conflict monitoring, but OCD patients showed significantly reduced N2 amplitudes across both Go and NoGo conditions, particularly over frontocentral electrodes including Fz, F3, F4, and Cz. The topography of the N2 response also differed between groups: whereas healthy controls showed a typical frontal/frontocentral distribution, with prominent activity around F4, patients with OCD showed a more posterior maximum, particularly around C3. Importantly, P3 amplitude did not differ between groups, suggesting that the main abnormality in this paradigm was concentrated in the earlier N2/N200 stage rather than in later stimulus evaluation or response-monitoring processes [22].

A related but partly opposite pattern was observed in the stop-signal study. Here, OCD patients showed a clear behavioural deficit in inhibitory control, expressed as significantly longer stop-signal reaction times than controls. In contrast, go reaction time, go accuracy, and the percentage of successful inhibitions did not differ significantly between groups. At the electrophysiological level, Stop-N2 was maximal frontally, especially at Fz, and was larger during failed than successful inhibition trials. However, crucially, OCD patients showed larger Stop-N2 amplitudes than controls, while Stop-N2 latency did not differ between groups. As in the Go/NoGo study, later P3 indices were relatively preserved, because Stop-P3 amplitude and latency did not significantly differ between groups. Thus, both studies point to N2/N200 as the primary ERP abnormality during inhibitory control in OCD, but they differ in direction: Go/NoGo performance was associated with reduced N2 amplitude despite intact behaviour, whereas stop-signal performance was associated with enhanced N2 amplitude alongside impaired stopping speed [23].

Findings from more complex cognitive-control paradigms further support this context-dependent interpretation. In adolescents with OCD performing an adapted backward-inhibition task-switching paradigm, the key demand was to return to a recently abandoned task set. Behaviourally, the OCD group showed a smaller backward-inhibition cost than healthy controls, indicating that they were not generally impaired in cognitive flexibility. Instead, they appeared to return more easily to a recently used or repetitive task set. However, this behavioural difference was not explained by N2/N200. Although N2 is often interpreted as a marker of conflict monitoring or cognitive control, the target-locked N2 measured at Cz did not show significant effects corresponding to the group difference in backward inhibition [24]. Therefore, the authors suggested that the relevant mechanism was not later conflict monitoring indexed by N2/N200, but earlier inhibitory gating reflected by P1. In this task, irrelevant task-set information may have been suppressed early enough that strong N2-related conflict processing was not required.

In contrast, another study provided more direct evidence that reduced N2/N200 may reflect impaired cognitive control in OCD, particularly under socially salient conditions. Adults with OCD and healthy controls completed a visual discrimination task embedded in a simulated social hierarchy. The N2 was measured as a stimulus-locked frontal component approximately 250–350 ms after stimulus onset over Fz, F3, F4, and Cz, and was interpreted as an index of cognitive control or conflict processing. OCD patients showed reduced N2 negativity compared with controls, which was interpreted as weaker cognitive control or impaired conflict adaptation. Importantly, this abnormality was not fixed across all contexts but depended on the social condition. When patients performed in the presence of a superior player, placing them in a socially inferior or evaluative position, the N2 abnormality was more pronounced. When they performed in the presence of an inferior player, placing them in a socially superior position, their N2 activity shifted closer to the control pattern [43].

Other paradigms provided weaker or less specific evidence for N2/N200 involvement. In an auditory prepulse-inhibition study, N2 was measured after the second auditory stimulus within a 221–320 ms window during an active discrimination task requiring responses to target tones and inhibition of responses to non-target tones. Although OCD patients showed behavioural impairment, including slower responses and more omission errors than controls, the N2 component did not show a reliable prepulse-inhibition effect. N2 amplitude was not meaningfully modulated by the short versus long S1–S2 interval, and there were no clear group differences indicating abnormal N2 processing in OCD. Thus, despite evidence of altered behavioural performance and reduced physiological gating on other measures, N2 did not appear to capture the cognitive consequences of prepulse interference. In this study, other ERP components, particularly P50, N1, and P3, were more informative, while cognitive-performance associations were mainly related to reaction-time slowing and later target-related P3 differentiation rather than to N2 activity [47].

Similarly, in a visual Go/NoGo repetition-priming study, N2 was measured at Fz between 180 and 400 ms during repeated Go stimuli. Both OCD patients and panic-disorder patients showed an attenuated frontal-greater-than-parietal N2 topography compared with healthy controls. This abnormality was present for baseline stimuli and during Go repetition processing, suggesting altered frontally distributed processing that may involve early control, monitoring, or stimulus-processing mechanisms. However, the N2 effect was not specific to OCD, because a similar alteration was observed in the panic-disorder group. In addition, although N2 latency decreased with repeated Go stimuli across the full sample, suggesting faster processing of repeated targets, this repetition-related latency effect was not uniquely abnormal in OCD. The more disorder-relevant effects in this study concerned N1 latency and P2 amplitude, especially in the non-washing/checking OCD subgroup, rather than N2. Therefore, although this study supports the presence of altered N2 topography in OCD, it does not establish N2/N200 as a specific marker of OCD-related cognitive dysfunction [54].

### 3.7. ERN (Error-Related Negativity)

Across the reviewed studies, the ERN emerged as one of the most consistently examined electrophysiological indices of performance monitoring in OCD, but its relationship with cognitive dysfunction was not uniform.

Several studies supported the classical view that OCD is characterised by exaggerated neural sensitivity to errors. In paediatric samples, youths with OCD repeatedly showed enlarged ERN amplitudes relative to healthy controls during modified Eriksen flanker tasks [33,34,37,38,39,40,45,50]. This effect was generally specific to error processing, because the correct-response negativity was often unchanged, suggesting that the abnormality did not simply reflect a general increase in response-related activity [34,37,38,40,42]. In some studies, this enlarged ERN appeared even when OCD patients and controls did not differ in reaction times, number of errors, or post-error slowing [33,34,40,42], indicating that heightened error-monitoring activity can be present without overt cognitive-performance impairment. This dissociation was also observed in familial-risk work, where both OCD patients and their unaffected first-degree relatives showed enlarged ERN amplitudes compared with healthy controls, despite no corresponding impairment in reaction time or post-error behavioural adjustment [42].

Nevertheless, other studies showed that ERN amplitude could be behaviourally meaningful, particularly in relation to accuracy. In paediatric OCD, larger ERN amplitudes were sometimes associated with better task accuracy or fewer errors [38,39,40,50]. For example, one large paediatric study found that OCD participants showed both reduced accuracy and enlarged ERN, while ERN independently predicted accuracy together with age, diagnosis, and later error-processing indices [50]. Similarly, other paediatric work reported that larger ERN activity was linked to better accuracy within the OCD group, whereas smaller ERN responses were associated with more errors [37,38].

The relationship between ERN and performance was also strongly dependent on task context. In standard flanker tasks, obsessive–compulsive symptoms were often associated with enlarged ERN or dERN amplitudes, consistent with exaggerated monitoring of clear motor-response errors [35,48]. By contrast, in probabilistic learning tasks, where errors are less explicit and depend on reinforcement history, OCD patients or individuals with high obsessive–compulsive symptoms did not consistently show enhanced ERN. Some studies found no significant response-locked ERN difference between OCD patients and controls during probabilistic learning, despite preserved or impaired learning performance [41,49]. Others found that higher obsessive–compulsive symptoms were associated with smaller ERN or dERN amplitudes following suboptimal choices [35,48].

Several studies therefore indicate that ERN abnormalities in OCD should not be interpreted as a single marker of either better or worse performance. Instead, they appear to reflect abnormal calibration of the performance-monitoring system across changing cognitive demands. In a recognition-memory paradigm, OCD patients showed exaggerated ERN-like monitoring after errorless learning, especially for risky “yes” responses, but reduced error-likelihood effects after errorful learning, when conflict between incorrect guesses and correct targets was stronger [36]. Behaviourally, OCD patients were not globally impaired in recognition memory and benefited from errorless learning similarly to controls.

Adult OCD studies showed more mixed results than paediatric studies. Some adult flanker studies reported enhanced ERN in OCD or related anxiety conditions [43,51], but others found no significant group difference in ERN or CRN despite poorer performance or more severe clinical symptoms [49,52]. In one study, OCD patients and healthy controls did not differ in ERN amplitude during a Go/NoGo task combined with affective word categorisation, even though OCD patients showed reduced affective priming after errors [25]. Similarly, in an anti-saccade EEG–fMRI study, OCD patients made slightly more errors, but ERN amplitude and latency did not differ from controls and were not significantly related to error rate or post-error adjustment [32]. Instead, brain–behaviour associations were stronger for fMRI measures, including reduced dorsal ACC activation with higher error rates and abnormal post-error modulation of default-network regions [32].

Comorbidity and clinical heterogeneity also influenced ERN findings. Paediatric studies suggested that enlarged ERN may be especially pronounced in non-tic-related OCD, whereas youths with tic-related OCD sometimes resembled healthy controls [37]. Other work found enhanced ERN in both tic-related and non-tic-related paediatric OCD, and in both current and past OCD, suggesting that the effect may be relatively robust but not identical across phenotypes [50]. Comorbid depression also appeared relevant: adolescents with OCD plus major depressive disorder showed the strongest ERN enlargement, whereas OCD without depression showed a weaker or trend-level effect [39]. At the same time, major depressive disorder without OCD did not show comparable ERN enhancement [39]. Transdiagnostic findings further complicate the interpretation, because enhanced ERN was also observed in health anxiety and non-OCD anxiety disorders [34,51]. Therefore, enlarged ERN is not specific to OCD, but may reflect a broader dimension of heightened error sensitivity, threat monitoring, uncertainty processing, or checking-related cognitive style.

Several studies also linked ERN abnormalities to neural mechanisms beyond the time-domain ERP amplitude. Time–frequency analyses showed that adolescents with OCD had increased error-related mid-frontal theta power, and stronger theta was associated with larger ERN amplitudes [45]. In a larger paediatric sample, OCD was associated with increased theta total power across response types, whereas theta phase consistency did not differ from controls [46]. This suggests that OCD-related response-monitoring abnormalities may be driven partly by increased mid-frontal theta power rather than by more precise phase-locked oscillatory activity. However, the cognitive meaning of this theta–ERN enhancement remained complex. In one study, larger ERN and stronger theta were associated with faster responses, suggesting rapid or overactive control engagement rather than impaired behavioural adjustment [45]. In another, youths with OCD showed poorer Tower of London planning performance, but the reported associations between EEG indices and broader cognitive variables were small or exploratory [46].

Source-localisation and imaging findings pointed particularly to altered anterior cingulate cortex involvement. In high obsessive–compulsive symptom groups, flanker ERN was associated not only with dorsal ACC activity, but also with rostral ACC activity [48]. During probabilistic learning, high-OC participants showed disrupted reciprocal dorsal–rostral ACC patterns, indicating that similar behavioural performance may be supported by different neural mechanisms in individuals with greater obsessive–compulsive symptoms [48]. The anti-saccade EEG–fMRI study similarly implicated dorsal ACC and default-network abnormalities in behavioural error processing, even though ERN itself did not differ significantly between OCD and controls [32].

### 3.8. FRN (Feedback-Related Negativity)

Across the four studies, the feedback-related negativity (FRN) was examined mainly in paradigms requiring participants to use external feedback to guide later behaviour, including reversal learning, probabilistic reinforcement learning, and modified Iowa Gambling Task (IGT) procedures. In study [30], OCD patients showed clear behavioural impairment in feedback-guided reversal learning. They made more total errors than controls, particularly more reversal errors, reflecting continued selection of a previously rewarded stimulus after it became incorrect, and more exploration errors, reflecting repeated selection of an already-disconfirmed alternative during the search for a new correct response. Reaction times did not differ between groups, indicating that the deficit was not simply due to slower responding but to poorer use of negative feedback for behavioural updating. At the ERP level, the critical finding was a reduced FRN in OCD patients specifically during exploration negative feedback. In contrast, FRN responses to reversal negative feedback did not differ between patients and controls. Importantly, this FRN abnormality remained significant even after perseveration and exploration errors were included as covariates. Study [49] extended this issue by examining the FRN/RewP complex during active and observational probabilistic learning. Behaviourally, OCD patients performed worse than healthy controls in learning stimulus–reward contingencies, especially for the easier and more reliable reward pairs. They also showed a more indecisive choice pattern, with greater shifting and especially more shifting after wins, suggesting reduced confidence in positive feedback. The FRN/RewP results did not indicate exaggerated punishment processing in OCD. Instead, OCD patients showed generally more positive or attenuated FRN amplitudes, mainly because win feedback elicited abnormally positive FRN/RewP responses during active learning. Loss responses did not significantly differ from controls. In computational analyses, FRN amplitudes were modulated by prediction-error estimates during active learning, indicating that this component tracked reward expectancy and feedback unexpectedness when participants learned from their own actions. Therefore, in this study, poorer feedback learning in OCD was linked to abnormal reward-feedback processing rather than to a simple enhancement of negative-feedback monitoring. A different pattern emerged in the modified IGT studies. In study [56], clinical OCD patients showed impaired ambiguous decision-making compared with healthy controls. They had lower IGT net scores and did not show the same adaptive improvement across task blocks, suggesting reduced ability to learn from feedback and shift toward the advantageous option. ERP results showed that both OCD and OCPD groups had larger loss-minus-win FRN difference-wave amplitudes than controls, with the OCD-control difference reaching significance. The dFRN was strongest at FCz and was larger after disadvantageous choices than advantageous choices, suggesting heightened neural differentiation of unfavourable versus favourable outcomes in obsessive–compulsive groups. Crucially, FRN was directly related to cognitive performance: IGT net scores were negatively correlated with average FRN difference-wave amplitude, and this association was especially evident after disadvantageous choices. Because the difference wave represented loss-minus-win negativity, larger/more negative FRN differentiation was associated with worse decision-making performance. Thus, in this task, stronger neural sensitivity to unfavourable feedback did not translate into better learning; instead, it was linked to poorer adaptive decision-making. Study [57] showed a similar pattern in individuals with subclinical obsessive–compulsive symptoms. Participants with high obsessive–compulsive traits showed poorer IGT performance than low-trait controls: they failed to improve across blocks and continued choosing the disadvantageous option more often. At the ERP level, they showed larger FRN responses to loss than win feedback, and the loss-minus-win FRN difference was markedly larger than in controls. Source localisation suggested greater anterior prefrontal/orbitofrontal activity during loss feedback, while the usual relationship between anterior prefrontal and dorsal anterior cingulate activity appeared disrupted. Importantly, within the subclinical obsessive–compulsive group, larger FRN amplitudes were associated with lower IGT net scores.

### 3.9. CNV (Contingent Negative Variation)

The evidence concerning the CNV component in OCD was very limited, because only one study assessed this measure, and CNV was not the main electrophysiological outcome of the experiment. In study [47], CNV was examined in the context of prepulse inhibition during an active auditory discrimination task comparing patients with OCD, patients with schizophrenia, and healthy controls. Participants heard a brief prepulse click followed either 100 ms or 500 ms later by a target or non-target tone. The task required active discrimination of the second stimulus, with participants responding to the high tone and ignoring the low tone. Thus, the paradigm was relevant to cognitive functioning because it combined early sensory gating with attentional discrimination, response preparation, and speeded decision-making. In this study, CNV was measured at S2 onset in the 500 ms condition, where the longer interval between the prepulse and the second stimulus allowed the development of anticipatory activity before the discriminative tone. However, the available results do not indicate a clear OCD-specific abnormality in CNV amplitude. CNV was included among the physiological variables examined in relation to behavioural performance, but the study did not identify CNV as the principal electrophysiological marker distinguishing OCD patients from healthy controls. Instead, the strongest ERP findings concerned other components, particularly P50, N1, and P3. Healthy controls showed P50 suppression in the short 100 ms condition relative to the 500 ms condition, whereas this effect was less clearly present in the clinical groups. N1 showed a robust prepulse effect across all groups, while P2 and N2 were not meaningfully modulated. Later cognitive processing, especially target-related P3 differentiation, appeared more closely linked to discrimination performance than early sensory-gating measures. Behaviourally, the OCD group showed some evidence of altered task performance. Patients with OCD made more omission errors than controls, responded more slowly, and showed a more conservative response style. However, their perceptual sensitivity was less impaired than that of the schizophrenia group. The short 100 ms prepulse interval increased omission errors and tended to slow responses, indicating that close temporal interference from the prepulse affected target detection and response execution. The relationship between electrophysiological measures and cognitive performance further limits the interpretation of CNV as a marker of cognitive dysfunction in OCD. Regression analyses indicated that physiological and behavioural prepulse-related measures explained a substantial proportion of variance in omission errors, perceptual sensitivity, and response criterion. Nevertheless, the clearest cognitive association was observed for reaction-time prepulse inhibition: greater slowing in the short-interval condition was associated with poorer perceptual sensitivity and a more conservative response criterion. In contrast, early physiological gating indices such as P50-PPI and EMG-PPI were not significantly related to discrimination performance, and the summary does not report a specific CNV-performance association. Moreover, later target-processing indices, particularly P3 differentiation between target and non-target stimuli, were more strongly related to omission errors and perceptual sensitivity.

### 3.10. LRP (Lateralised Readiness Potential)

In the only study examining the lateralised readiness potential in relation to cognitive dysfunction in OCD, the response-locked LRP-r was used as an electrophysiological marker of motor preparation, motor control, and response inhibition during a socially framed visual discrimination task [43]. Participants were required to decide which of two dot arrays contained more red dots, but the task was embedded in a simulated social hierarchy in which they performed either in the presence of a superior player, placing them in a socially inferior position, or in the presence of an inferior player, placing them in a socially superior position. This design was intended to test whether social-evaluative context modulates electrophysiological abnormalities in OCD, including abnormalities in motor-control processes. The LRP-r results indicated altered motor preparation and response-control processing in OCD. Across conditions, patients with OCD showed larger LRP-r amplitudes than healthy controls, suggesting abnormal recruitment of motor-control mechanisms during task performance. Because the LRP-r reflects lateralised motor preparation around the time of response execution, this finding was interpreted as evidence that OCD is associated not only with altered error monitoring and cognitive control, but also with abnormalities in the motor implementation or inhibition of responses. Importantly, this motor-control abnormality was not fixed across contexts. The study reported a significant Group × Hierarchy interaction, showing that social status strongly modulated LRP-r activity in OCD patients but not in controls.

Specifically, OCD patients showed larger LRP-r amplitudes when performing in the presence of the superior player, that is, when they were placed in a socially inferior position. In contrast, when they performed in the presence of the inferior player and occupied a socially superior position, their LRP-r amplitudes shifted toward the control range.

### 3.11. N450

The N450 component was examined in one study that investigated whether inhibition-related abnormalities in OCD differ between autogenous and reactive obsession subtypes [53]. The study used an emotional Stroop task to assess interference control, defined as the ability to suppress irrelevant stimulus information while performing a task-relevant colour-naming response. This distinction was clinically important because autogenous obsessions are typically internally generated, intrusive, ego-dystonic thoughts or images, whereas reactive obsessions are more often triggered by identifiable external stimuli. The authors therefore expected that autogenous OCD might be especially associated with impaired interference control. In the emotional Stroop task, participants responded to the colour of negative, positive, and neutral words while ignoring their emotional meaning. Behaviourally, the clearest abnormality appeared in the autogenous OCD group, which showed slower reaction times overall than both reactive OCD patients and healthy controls. Autogenous patients were particularly slow when naming negative words, although formal interference scores did not significantly differ between groups. The N450 was measured over frontal–central and central electrode sites and was interpreted as an electrophysiological index of conflict detection or conflict resolution during Stroop-like processing. The main N450 finding was that patients with autogenous OCD showed a reduced, or less negative, N450 response compared with healthy controls, especially at Cz and C4. This reduction was not restricted to negative words but was observed across negative, positive, and neutral word conditions. In numerical terms, the autogenous OCD group showed more positive N450 values than controls across all word types, indicating weaker N450 negativity. Importantly, the N450 abnormality appeared alongside other ERP changes in the autogenous group, including increased P2 and P3b amplitudes. Together, these findings indicate that autogenous OCD was associated with altered processing across multiple stages of the emotional Stroop task: early-to-middle stimulus classification or salience processing, later semantic/conflict-related evaluation, and N450-indexed conflict resolution. The evidence linking N450 directly to cognitive dysfunction is therefore suggestive but limited. The reduced N450 in autogenous OCD was consistent with poorer interference-control efficiency and occurred in the same group that showed generalised behavioural slowing during the Stroop task. However, the study did not report direct correlations between N450 amplitude and Stroop reaction time, accuracy, or interference scores.

### 3.12. P50

Across the two studies, the P50 component provided evidence for abnormal early sensory gating in OCD, but only weak evidence that this abnormality directly explains cognitive dysfunction. In study [28], P50 was examined using a standard auditory paired-click paradigm in 26 patients with OCD and 26 matched healthy controls. The central finding was that patients with OCD showed significantly poorer P50 suppression than controls, reflected in a higher P50 test/conditioning ratio. This ratio was 68.4% in the OCD group compared with 42.6% in controls, indicating that the response to the second auditory click was insufficiently inhibited. Importantly, this abnormality was not caused by a reduced initial response to the first stimulus, because conditioning P50 amplitude did not differ between groups. Instead, the OCD group showed a larger response to the second, test stimulus, suggesting a specific failure to suppress repeated sensory input. This effect remained significant after removal of statistical outliers, strengthening the interpretation that impaired P50 gating may represent a robust early inhibitory abnormality in OCD [28]. The same study also showed that patients with OCD had poorer performance on several neuropsychological measures, especially those involving processing speed, attention, psychomotor speed, executive attention, and cognitive flexibility. They performed worse than controls on the Symbol–Digit Modalities Test, Trail-Making Test A, Trail-Making Test B, and TMT B–A. By contrast, they did not differ from controls on verbal or visual paired-association learning or on digit span forward and backward. However, the key point for interpreting P50 is that the P50 test/conditioning ratio did not correlate significantly with any cognitive test score. It was also unrelated to OCD symptom severity, obsession scores, compulsion scores, illness duration, age at onset, or medication dose. Thus, although OCD patients showed both impaired P50 sensory gating and poorer executive-attentional performance, these two abnormalities were not statistically linked within the OCD group [28]. Study [47] examined P50 in a different context: not a passive paired-click paradigm, but an active auditory discrimination task involving prepulse inhibition. Participants included healthy controls, patients with OCD, and patients with schizophrenia. A brief prepulse was presented before a target or non-target tone, and the authors examined how the short prepulse interval influenced behavioural performance, EMG activity, and several ERP components, including P50. In healthy controls, P50 amplitude was reduced when the second stimulus followed the prepulse after 100 ms rather than 500 ms, consistent with normal prepulse inhibition of early auditory processing. This P50-PPI effect was not clearly demonstrated in OCD or schizophrenia. However, the P50 group effect in OCD was not as robust as in schizophrenia and did not consistently survive stricter post hoc testing, so the finding should be interpreted cautiously. Behaviourally, OCD patients showed some impairment relative to controls, including slower responses and a more conservative response style, although their perceptual sensitivity was less impaired than that of schizophrenia patients [47]. Crucially, study [47] also suggested that early P50 gating was not the electrophysiological measure most closely related to cognitive performance. Regression and correlational analyses showed that physiological PPI measures could explain part of the variance in omission errors, perceptual sensitivity, and response criterion, but P50-PPI itself was not significantly correlated with discrimination performance. Similarly, EMG-PPI and N1-PPI were not significant predictors of task performance. Instead, later indices of target processing, especially target/non-target P3 differentiation, were more clearly associated with omission errors, perceptual sensitivity, and response strategy. Therefore, even when early sensory gating appeared attenuated in OCD, its functional relationship with cognitive performance was limited.

### 3.13. P600

Only one included study examined the relationship between the P600 component and cognitive dysfunction in OCD [26]. In this study, P600 was recorded during the preparatory phase of a digit-span working-memory task, specifically in the 1 s interval following an auditory warning cue and before presentation of the digit sequence. The task required participants either to recall digits in the same order or in reverse order, depending on the frequency of the warning tone. Thus, the P600 was interpreted not as an index of the recall response itself, but as a marker of late-stage anticipatory information processing during working-memory preparation [26]. Behaviourally, patients with OCD showed significantly poorer memory performance than healthy controls. Across the task, OCD patients recalled fewer digits than controls, indicating a measurable impairment in short-term/working-memory performance. However, performance did not differ substantially between same-order and reverse-order recall conditions, suggesting that both task variants imposed a comparable level of difficulty [26]. At the electrophysiological level, OCD was associated with increased P600 amplitudes. These amplitude increases were observed mainly over frontal, central, and right temporo-parietal regions, including F3, C3, F4, C4, Fz, and particularly C4-T6/2. The strongest group-discriminating effect was found at the right temporo-parietal C4-T6/2 site, where P600 amplitude was markedly higher in OCD patients than in controls. Multivariate and logistic-regression analyses indicated that this right temporo-parietal abnormality accounted for most of the group separation and classified approximately 80% of participants correctly as OCD or control [26]. This pattern suggests that OCD may involve exaggerated recruitment of late-stage preparatory or evaluative processes during working-memory task preparation, especially within right-lateralised temporo-parietal networks. P600 latency abnormalities were more restricted. OCD patients showed significantly prolonged P600 latency only at the right parietal P4 electrode, whereas other electrodes showed no significant latency differences. This finding suggests a focal delay in late-stage right parietal processing during working-memory preparation, rather than a generalised slowing of P600 activity across the scalp [26]. Importantly, the link between P600 abnormalities and cognitive dysfunction was not direct. Although OCD patients performed worse on the digit-span working-memory task, neither the increased P600 amplitude at C4-T6/2 nor the prolonged P600 latency at P4 was significantly correlated with memory performance. Therefore, this study provides evidence that P600 activity is abnormal in OCD and that patients show impaired working-memory performance, but it does not demonstrate that the P600 abnormality explains the observed cognitive deficit [26]. Instead, the P600 alterations appeared to be more closely related to clinical OCD characteristics, especially obsessional complaints and obsessive traits, than to actual task performance.

### 3.14. Pc (Correct-Response Positivity)

Across the two studies, evidence concerning the Pc component was limited but conceptually important. The Pc was directly examined only in study [52], where it was analysed together with the Pe as part of the later response-locked positivity occurring approximately 170–270 ms after a motor response. In this study, the Pe/Pc complex was interpreted as a later internal feedback signal related to awareness of whether one’s own action had been correct or incorrect. This interpretation is particularly relevant for OCD, because many compulsive behaviours may reflect not only excessive concern about errors, but also a reduced sense that an action has been completed correctly.

In study [52], OCD patients and healthy controls performed a visual flanker task requiring fast responses to the direction of a central arrow while ignoring congruent or incongruent flankers. Behaviourally, the task produced the expected interference effect: both groups were slower and made more errors on incongruent trials than on congruent trials. OCD patients were not significantly slower than controls on correct trials, but they committed substantially more errors, especially in the incongruent condition. Thus, at the behavioural level, the study indicated impaired response control or conflict-related performance in OCD. However, these behavioural indices did not correlate significantly with OCD severity, insight, anxiety, or treatment resistance, suggesting that overt task performance alone did not capture the clinically relevant dimension of dysfunction in this sample.

The main Pc-related finding was that OCD patients showed a marked disruption of the later positive response-monitoring complex. In healthy controls, the expected Pe/Pc differentiation was present: incorrect responses elicited a larger Pe than the Pc elicited by correct responses, especially over the medial scalp. This pattern suggests that controls generated a clear internal signal distinguishing erroneous from correct actions. In OCD patients, by contrast, the Pe/Pc complex was described as virtually absent in the median region where this positivity is usually most prominent. Patients did not show a meaningful differentiation between Pe and Pc, indicating that the neural signal normally supporting awareness of response correctness was strongly reduced.

Study [47] did not examine Pc as a response-locked correct-response positivity. However, it provides indirect support for the broader idea that later positive ERP components may be more closely related to cognitive performance than early sensory-gating indices. In that study, early PPI-related measures such as P50-PPI, EMG-PPI, and N1-PPI were not significantly associated with discrimination performance. In contrast, later target-related P3 differentiation was related to omission errors, perceptual sensitivity, and response criterion. Although this result concerns P3 rather than Pc, it is consistent with the interpretation that later positive ERP activity, reflecting higher-order stimulus or response evaluation, may be more relevant to cognitive performance than early automatic sensory gating.

### 3.15. Pe/Error Positivity

Across the reviewed studies, the Pe component provided mixed but informative evidence for altered later-stage error processing in OCD and obsessive–compulsive traits. Unlike the ERN, which more consistently indexed exaggerated early error monitoring, Pe findings suggested that later error evaluation, conscious appraisal of mistakes, and the differentiation between erroneous and correct actions may be altered in a more context-dependent way.

In study [43], Pe was examined together with ERN during a socially framed visual discrimination task in adults with OCD. Patients showed larger Pe amplitudes than healthy controls, consistent with enhanced later error-related processing. However, this abnormality depended strongly on social hierarchy. Pe amplitudes were especially increased when OCD patients performed in the presence of a superior player, that is, when they occupied a socially inferior position. In contrast, when they performed in a superiority position, Pe amplitudes shifted toward the control range.

In study [44], conducted in a nonclinical sample of 10-year-old children, obsessive–compulsive behaviours were also associated with enhanced Pe activity. Children with more parent-rated obsessive–compulsive behaviours showed larger Pe amplitudes at Cz, and the group with at least some OC behaviours had significantly larger Pe amplitudes than children with no OC behaviours. Importantly, Pe was related to behavioural accuracy. Children who made fewer errors tended to show larger Pe amplitudes, whereas those who made more errors showed smaller Pe responses. Pe was not meaningfully related to error-trial reaction time or post-error slowing, indicating that its behavioural relevance was more closely tied to accuracy and cautious performance than to speed adjustment after errors.

In study [45], which examined adolescents with clinically diagnosed OCD, the Pe showed a different pattern. Although the OCD group displayed an enlarged ERN, Pe amplitudes did not differ significantly between patients and controls.

The largest paediatric study [50] provided stronger evidence that later error-related positivity may be relevant to cognitive performance in OCD when Pe is examined relative to correct-trial positivity. In this study, raw Pe amplitude was not significantly reduced in OCD compared with controls. However, the ΔPe, defined as the difference between Pe and Pc, was significantly smaller in children and adolescents with OCD. This means that the later positivity did not distinguish errors from correct responses as clearly in OCD as it did in healthy controls. Functionally, this reduced ΔPe suggests weaker differentiation between “I made an error” and “I responded correctly.” This finding was observed in both tic-related and non-tic-related OCD. Moreover, ΔPe appeared more closely linked to current OCD than to past OCD, suggesting that later error/correct differentiation may be more state-sensitive than the ERN.

Study [50] also directly linked Pe-related activity to cognitive performance. Across participants, higher flanker-task accuracy was associated with larger Pe amplitudes and, more strongly, with larger ΔPe. In regression analyses, ΔPe remained an independent predictor of accuracy even when age, lifetime OCD diagnosis, and ERN were included in the model. Thus, better cognitive performance was associated not only with stronger early error monitoring but also with a stronger later distinction between erroneous and correct responses.

In study [52], adults with OCD showed a more severe disruption of the later positive error-monitoring complex. Whereas healthy controls displayed the expected pattern in which Pe after errors was larger than Pc after correct responses, OCD patients showed a markedly reduced or virtually absent Pe/Pc differentiation over the median scalp region. The authors interpreted this as evidence for impaired internal feedback about whether one’s own action was correct. This finding is conceptually important for cognitive dysfunction in OCD because it suggests that some patients may not lack the ability to perform actions, but may have a weakened internal signal confirming that an action was completed correctly. Such a deficit could help explain repetitive checking behaviour: the individual may continue checking not because the action was not performed, but because the internal electrophysiological marker of action correctness is insufficiently distinct. However, this study did not report direct correlations between Pe/Pc amplitudes and behavioural performance measures such as error rate or reaction time, so its relevance to cognitive dysfunction is mainly mechanistic rather than directly performance-correlational.

### 3.16. Plain EEG, Quantitative EEG, Source-Localised EEG, or Time–Frequency Oscillatory Activity

Across the studies that examined plain EEG, quantitative EEG, source-localised EEG, or time–frequency oscillatory activity, the relationship between electrophysiological abnormalities and cognitive performance in OCD was heterogeneous but consistently pointed toward altered oscillatory regulation of cognitive control, performance monitoring, executive speed, visuospatial memory, and attentional flexibility. These studies differed from ERP-focused investigations because they did not primarily interpret cognitive dysfunction through discrete time-locked components such as the ERN, P300, or old/new effect. Instead, they examined broader spectral or oscillatory features of EEG activity, including resting-state delta, theta, alpha, and beta power, hemispheric asymmetry, source-localised current density, and task-related theta, alpha, and beta dynamics.

Two paediatric studies examined time–frequency EEG during response monitoring and cognitive control. In study [45], adolescents with OCD and matched healthy controls performed an arrow flanker task while EEG was analysed not only in the ERP domain but also in delta, theta, alpha, and beta frequency bands. Behaviourally, the OCD and control groups did not differ in the number of errors or in post-error slowing, although the OCD group responded faster overall, particularly on error trials. The time–frequency results showed that errors elicited a broad oscillatory response across the whole sample, including increased delta and theta power and suppression of alpha and beta activity. The group differences were most evident in theta and beta bands. Adolescents with OCD showed greater frontal and frontocentral theta power after errors than controls, while theta responses to correct trials did not differ between groups. This indicates that the theta abnormality was error-specific and related to performance-monitoring demands rather than reflecting a generalised elevation of EEG power. OCD patients also showed greater beta power following errors, which, in the context of normal error-related beta suppression, means that they showed reduced beta suppression after mistakes. Thus, the oscillatory abnormality in OCD involved both an exaggerated theta response and an altered beta response during error processing.

The correlations with cognitive performance in study [45] showed that theta and beta had different behavioural meanings. Greater error-related beta activity was associated with slower reaction times on error trials and slower post-error reaction times, suggesting that reduced beta suppression was linked to behavioural inhibition, delayed motor re-engagement, or excessive post-error caution. In contrast, greater theta activity was associated with faster correct-trial, post-error, and post-correct reaction times. Therefore, theta enhancement in OCD did not simply represent cognitive impairment or behavioural slowing.

Study [46] extended this line of evidence in a larger paediatric and adolescent sample by examining whether OCD-related response-monitoring abnormalities were better explained by mid-frontal theta total power or by theta inter-trial phase coherence. Youth with OCD performed slightly less accurately than healthy controls on the flanker task, especially on incongruent trials, and also showed poorer Tower of London planning performance, with more total moves and fewer correctly solved problems. EEG analysis showed that theta-band total power was greater after errors than correct responses across participants, confirming the role of theta activity in response monitoring. Importantly, youth with OCD showed elevated theta total power across response types. However, the group-by-response interaction was not significant, indicating that OCD was not characterised by a selectively exaggerated error-minus-correct theta response. Instead, the abnormality reflected a more generalised elevation of response-related theta power. By contrast, theta phase coherence increased after errors in both groups but did not differ between OCD and control participants.

The relationship between EEG and cognitive performance in study [46] was more limited than in study [45]. Although the OCD group showed both elevated theta power and measurable cognitive-performance differences, exploratory associations between EEG measures and clinical or behavioural variables, including Tower of London performance, were small overall.

Resting-state source-localised EEG provided stronger evidence that EEG abnormalities may distinguish cognitively impaired from cognitively intact OCD patients. In study [58], patients with OCD and healthy controls underwent resting eyes-closed EEG, and cortical current density was estimated using sLORETA. Cognitive performance was assessed using the Trail-Making Test, Stroop Colour-Word Test, and D2 Test. The most prominent cognitive impairment in the OCD group was observed on TMT-A and TMT-B, indicating slowing in visual scanning, psychomotor speed, attention, information processing, eye–hand coordination, set shifting, mental flexibility, and executive control. The EEG results showed increased low-frequency cortical source activity in OCD, especially in delta and theta bands. Delta increases were widespread but strongly involved frontal and frontotemporal regions, including medial frontal, orbitofrontal, anterior cingulate, cingulate, insular, temporal, and limbic-related areas. Theta increases were more restricted and were mainly localised to frontal regions.

The most important finding in study [58] was that resting EEG abnormalities were not present equally in all OCD patients. When patients were divided according to TMT-A performance, cognitively unimpaired OCD patients did not differ from healthy controls in cortical EEG source activity. By contrast, cognitively impaired OCD patients showed significant increases in delta and theta source activity. This indicates that the low-frequency EEG abnormality was associated with cognitive dysfunction rather than OCD diagnosis alone. The relationship was not presented primarily as a simple linear correlation between EEG power and test score, but as a subgroup-based association: frontal and fronto-limbic low-frequency abnormalities were present in patients with measurable cognitive impairment and absent in those with preserved cognitive performance.

Resting qEEG asymmetry findings also linked plain EEG abnormalities to domain-specific cognitive dysfunction. In study [59], unmedicated and non-depressed OCD patients and healthy controls completed delayed memory tests, including logical memory as a measure of verbal memory and visual reproduction as a measure of nonverbal or visuospatial memory. The OCD group showed impaired delayed visual reproduction but preserved delayed logical memory, indicating a selective visuospatial or nonverbal memory deficit rather than a generalised memory impairment. Resting qEEG revealed reduced absolute power in OCD, especially in delta, beta1, and beta2 bands, with reductions most evident over frontal and right-hemisphere sites. Hemispheric asymmetry analyses indicated relatively lower right-sided EEG power in OCD. This pattern was interpreted as evidence of right-hemisphere hypofunction.

The most relevant EEG–cognition relationship in study [59] concerned beta2 asymmetry. Greater left–right beta2 asymmetry, reflecting relatively reduced right-sided beta2 power, was associated with poorer delayed visual reproduction performance. Conversely, relatively greater left-sided beta2 power was associated with better delayed logical memory performance. Some of these associations were limited by the small sample and did not all reach significance at the summary-score level, but the strongest association with visual reproduction was observed at posterior temporal sites. The overall pattern therefore suggested a lateralised and domain-specific EEG–cognition relationship: reduced right-hemisphere beta2 activity was linked to poorer visuospatial memory, whereas relatively preserved or stronger left-sided activity was related to verbal memory performance.

Study [60] examined resting qEEG in relation to executive functioning, attention, short-term memory, and sequence learning. Drug-free OCD patients and matched controls underwent multi-lead resting qEEG, and the OCD group completed neuropsychological tests including Spatial and Non-Spatial Conditional Associative Learning tasks and the Self-Ordered Pointing Task. The main EEG abnormality was reduced alpha activity, especially in the slow alpha band. In the full sample, OCD patients showed lower theta2, alpha1, and alpha2 absolute power than controls, but after excluding patients with higher depressive symptoms, the most stable abnormality was reduced alpha1 power. In relative power analyses, OCD patients showed increased delta and reduced alpha activity, again with the most robust finding centred on reduced slow alpha.

The relationship between EEG and cognition in study [60] was expressed mainly through executive performance speed. In the OCD group, reduced relative alpha1 power was associated with slower performance on the drawing version of the Self-Ordered Pointing Task. Since this task requires self-organisation, monitoring, response selection, and executive control, the finding indicated that patients with stronger slow-alpha reduction required more time to complete an executive task. Higher beta relative power was also associated with slower performance on the Self-Ordered Pointing Task, including both drawing and word versions. Thus, reduced slow alpha and increased beta activity were both related to longer task completion time. Importantly, these EEG abnormalities were not significantly correlated with obsessive–compulsive symptom severity or depressive symptoms, suggesting that they were more closely related to cognitive performance speed than to clinical severity.

Study [61] added task-related oscillatory evidence from a selective-attention paradigm. Patients with OCD and healthy controls performed a task requiring attention to either colour or shape while ignoring irrelevant stimulus features. The study also assessed executive functioning using the Wisconsin Card Sorting Test, making it directly relevant to the relationship between oscillatory EEG and cognitive flexibility. Behaviourally, OCD patients performed slightly faster than controls, while accuracy was similar, suggesting that EEG abnormalities could not be explained merely by worse task performance. The main EEG finding was markedly reduced prestimulus alpha and theta power in OCD. Prestimulus alpha was lower in OCD patients than controls, and the normal occipital alpha distribution seen in controls was altered in patients. The authors interpreted reduced prestimulus alpha as impaired top-down inhibitory control, because alpha synchronisation before stimulus onset is thought to suppress task-irrelevant processing. Prestimulus theta power was also reduced in OCD, especially over frontal regions.

The correlations in study [61] provided direct evidence linking oscillatory EEG to executive dysfunction. Within the OCD group, evoked alpha power at Oz during the colour task correlated positively with perseverative responses on the Wisconsin Card Sorting Test. This means that patients with stronger occipital evoked alpha activity tended to show more perseveration, reflecting poorer cognitive flexibility and difficulty shifting set. Evoked theta power at Oz also correlated positively with perseverative responses during both colour and shape conditions. In addition, evoked theta power at Oz during the colour task correlated negatively with the number of WCST categories achieved, indicating that higher theta activity was associated with poorer executive performance.

Study [62] further connected resting EEG alpha activity with visuoconstructional and nonverbal cognitive performance. In this study, OCD patients underwent resting EEG alpha-power analysis and completed the Rey–Osterrieth Complex Figure Test. The authors focused on whether regional alpha activity predicted copy, immediate recall, and delayed recall performance. The most important finding concerned RCFT copy performance, which depends on visuospatial organisation, planning, perceptual integration, and constructional strategy. Frontal alpha power was significantly associated with copy performance, whereas alpha power in parietal, temporal, and occipital regions was not. Importantly, the direction of the relationship differed between hemispheres. Lower left frontal alpha power, interpreted as greater left frontal activation, was associated with poorer copy performance. In contrast, lower right frontal alpha power, interpreted as greater right frontal activation, was associated with better copy performance. Therefore, frontal activation had opposite cognitive meanings depending on hemisphere.

### 3.17. Risk-of-Bias Assessment

The risk-of-bias assessment based on ROBINS-I is presented in Table 3.

The included studies were methodologically heterogeneous and differed substantially in participant age, clinical characteristics, medication status, EEG methodology, cognitive paradigms, and statistical analysis. Most investigations used non-randomised case–control or cross-sectional designs comparing patients with obsessive–compulsive disorder with healthy participants. Several studies additionally included unaffected siblings, psychiatric comparison groups, OCD subtypes, or nonclinical participants selected according to the severity of obsessive–compulsive traits. Consequently, the principal risks of bias concerned confounding, participant selection, missing EEG data, multiplicity of electrophysiological outcomes, and selective reporting. The randomised or interventional elements identified in a smaller subset of studies were additionally considered using the RoB-2 domains.

In studies assessed using ROBINS-I, bias due to confounding represented one of the most persistent concerns. Although most clinical studies matched OCD and control groups for age and sex, and frequently also for education, handedness, or intelligence, matching did not fully account for clinically important differences between participants. Medication exposure varied considerably. Some studies recruited drug-naïve or medication-free patients, whereas others included participants receiving serotonin reuptake inhibitors, other psychotropic medications, or psychotherapy. In several studies, previously treated patients completed medication washout periods, but the duration of washout differed and could not exclude longer-lasting treatment effects. Medication status was therefore a plausible confounder of both EEG activity and cognitive performance, particularly for components related to attention, inhibitory control, working memory, and error monitoring.

Psychiatric comorbidity was another important source of potential confounding. Some studies excluded depressive, anxiety, tic, neurological, developmental, and substance-related disorders, thereby producing clinically homogeneous samples. Other studies permitted comorbid depression, anxiety disorders, or tic-related OCD, and several patient groups showed higher depressive, anxiety, or worry scores than healthy controls even when formal comorbid diagnoses were excluded. These symptoms may independently influence reaction time, error frequency, executive performance, P300 amplitude, ERN magnitude, frontal theta activity, and other EEG measures. A number of studies examined comorbidity or symptom dimensions statistically, but adjustment was inconsistent and was often limited by small sample sizes. Differences in illness duration, age at onset, symptom severity, treatment resistance, and OCD subtype introduced further residual confounding. Accordingly, observed EEG–cognition relationships cannot always be attributed specifically to OCD rather than to associated clinical characteristics.

Bias in the selection of participants was generally moderate and was primarily related to convenience sampling and restrictive eligibility criteria. Clinical participants were commonly recruited from specialist outpatient clinics or university hospitals, while healthy controls were recruited from hospital staff, local volunteers, advertisements, or university communities. Such recruitment methods may produce groups that differ in motivation, socioeconomic background, familiarity with research procedures, or access to treatment. Restrictive exclusion criteria improved internal validity but reduced representativeness, particularly where patients with common comorbidities were excluded. The findings may therefore apply more readily to relatively young, cognitively able, cooperative, and clinically stable patients than to the broader OCD population.

Selection concerns were particularly relevant in studies that excluded participants because of poor EEG quality, excessive artefacts, insufficient numbers of errors, or inadequate task performance. These exclusions are methodologically understandable because reliable ERN, FRN, P300, or time–frequency estimates require sufficient artefact-free trials. Nevertheless, excluding participants who made too few errors or could not complete the task may systematically remove individuals with unusually good performance, severe impairment, poor attention, greater motor activity, or difficulty following instructions. This can change both the clinical composition of the analysed sample and the estimated association between EEG activity and cognitive performance. In paediatric studies, movement-related EEG loss may have been especially consequential. Several studies transparently reported exclusions, but sensitivity analyses comparing included and excluded participants were rarely described.

Classification of OCD status was generally a relative strength. Most clinical studies diagnosed OCD using DSM-based criteria, structured or semi-structured psychiatric interviews, and validated measures such as the Yale–Brown Obsessive–Compulsive Scale or its paediatric version. Misclassification of diagnosed OCD was therefore unlikely to be a major source of bias in these studies. Greater concerns applied to dimensional and subclinical investigations in which participants were classified according to questionnaire scores, parent-reported behaviours, or extreme percentiles of obsessive–compulsive symptoms. Such designs are informative for examining continuous traits but cannot be interpreted as equivalent to studies of clinically diagnosed OCD. In some subclinical samples, current or previous psychiatric disorders were not comprehensively assessed, creating uncertainty about whether the electrophysiological findings reflected obsessive–compulsive traits, other unmeasured psychopathology, or general negative affectivity. Classification of clinical subtypes, such as washing, checking, autogenous, reactive, or tic-related OCD, also depended on relatively small groups and may have been vulnerable to instability or overlap between symptom categories.

Bias due to deviations from the intended exposure was usually less prominent because OCD diagnosis was not experimentally assigned and the EEG tasks were generally administered using standardised protocols. Nevertheless, concurrent medication, psychotherapy, variable washout periods, fatigue, practice effects, and differences in task engagement could have altered the measured exposure–outcome relationship. Studies using long paradigms containing several hundred or more than one thousand trials were particularly vulnerable to fatigue, habituation, declining attention, and differential task persistence. When individualised calibration procedures were used to generate sufficient errors, these procedures improved ERP reliability but may also have resulted in participants completing tasks with different effective difficulty levels.

Missing outcome data ranged from a relatively minor concern to a potentially important source of bias. Many studies reported only small numbers of exclusions and retained most recruited participants. However, the typically small clinical samples meant that even the removal of a few participants could materially affect statistical power and effect estimates. Complete-case analyses were common, and formal methods for addressing missing EEG, behavioural, or questionnaire data were seldom described in the summaries. The risk was higher when exclusions were directly related to task performance or EEG quality because the probability of missingness could have been associated with both clinical status and the cognitive outcome. Studies requiring a minimum number of error trials were especially susceptible to this problem.

Measurement of EEG and computerised cognitive outcomes was generally objective and represented an important methodological strength. Most studies used established oddball, Go/NoGo, stop-signal, flanker, Stroop, reversal-learning, working-memory, sensory-gating, or task-switching paradigms. EEG recordings commonly included impedance control, electro-oculographic monitoring, baseline correction, artefact rejection, and predefined latency windows. Reaction time, accuracy, error rates, stop-signal reaction time, and neuropsychological test scores were usually measured using standardised procedures. These characteristics reduced the likelihood that knowledge of diagnostic status directly influenced outcome recording.

Nevertheless, important measurement heterogeneity remained. Studies differed in the number and placement of electrodes, online and offline references, filter settings, artefact thresholds, baseline periods, component definitions, peak-versus-mean amplitude measures, source-localization procedures, and the minimum number of accepted trials. Components with the same name were sometimes derived from substantially different tasks and therefore did not necessarily represent identical cognitive operations. Estimates based on few error trials or small numbers of artefact-free epochs may have had limited reliability. Outcome-assessor or EEG-analyst blinding to diagnostic group was also rarely described. Although automated processing reduced subjective influence, visual artefact rejection, selection of peaks, choice of electrodes, and interpretation of complex waveforms could still have been affected when blinding was absent or unclear.

The possibility of selective reporting was an additional concern. The studies frequently analysed multiple ERP components, latencies, amplitudes, frequency bands, electrode locations, task conditions, behavioural indices, clinical scales, and correlation coefficients. Some investigations applied Bonferroni or other corrections for multiple comparisons, whereas others presented uncorrected exploratory associations. In at least one study, apparently meaningful correlations between frontal P300 amplitude and Trail-Making Test performance did not remain significant after correction for multiple testing. Several other findings were limited to individual electrodes, latency windows, subgroups, or symptom dimensions. These results may be biologically plausible, but their credibility is reduced when the analytical plan was not prospectively specified. Preregistration was explicitly reported in at least one investigation, but it was not described in most of the study summaries. The absence of accessible protocols or prespecified analysis plans made it difficult to determine whether all measured outcomes and analytical contrasts were reported.

For studies containing genuinely randomised intervention components and assessed with RoB-2, objective EEG and computerised behavioural measures generally reduced bias in outcome measurement. However, the adequacy of sequence generation, allocation concealment, participant blinding, investigator blinding, and prespecified analysis was not consistently evident from the summaries. When data were taken from one session of a broader crossover or stimulation project, period effects, carryover effects, and deviations from the assigned condition would also require consideration. Exclusions for inadequate EEG quality or insufficient trials remained relevant to the missing-outcome domain, while the large number of possible ERP and behavioural endpoints raised concerns about selection of the reported result. Random ordering of stimuli or task conditions should not itself be interpreted as random allocation of participants and is therefore insufficient to establish a low risk of bias in the RoB-2 randomization domain.

## 4. Discussion

Cognitive dysfunction represents a clinically meaningful burden for patients with OCD. Numerous behavioural studies have demonstrated impairments across several cognitive domains, particularly executive functioning, inhibitory control, cognitive flexibility, attention, working memory, and performance monitoring. Electrophysiological studies using EEG and ERPs provide a temporally precise window into the neural correlates of these impairments and may inform hypotheses about their underlying mechanisms.

The neural mechanisms underlying OCD have been extensively investigated using a range of EEG and ERP measures. Several previous reviews have synthesised abnormalities in specific electrophysiological components associated with OCD [63,64,65]. However, none has systematically examined how these electrophysiological findings relate to cognitive functioning or cognitive dysfunction in the disorder. To our knowledge, the present review is the first to provide such an integrated synthesis.

The literature included in this review is substantial, but also heterogeneous and methodologically dispersed. Studies differed in the ERP components examined, cognitive paradigms used, clinical characteristics of OCD samples, medication status, symptom dimensions, and the extent to which electrophysiological findings were directly related to behavioural or neuropsychological measures. Therefore, rather than treating EEG and ERP abnormalities as isolated findings, this discussion integrates the available evidence mechanistically. Specifically, this section explains the neural mechanisms suggested by electrophysiological findings that were associated with cognitive performance in individuals with OCD, with the aim of clarifying how altered temporal dynamics of brain activity may contribute to the cognitive dysfunction observed in this disorder.

### 4.1. Mechanisms of P3/P300

The reviewed findings suggest that P3/P300 abnormalities in OCD should not be interpreted as a single, uniform electrophysiological deficit. Instead, they point to a disruption of late-stage cognitive operations that are recruited differently depending on whether the task requires target detection, interference control, response inhibition, feedback-guided learning, cognitive flexibility, or processing of personally salient emotional material. This interpretation is consistent with the broader P300 literature, in which the P3/P300 is not viewed as a unitary marker of “cognition” but as a family of late positive responses reflecting the allocation of attentional resources, stimulus evaluation, context updating, decision formation, and the linking of perceptual categorisation to action selection [66,67,68,69,70,71]. Therefore, the inconsistent direction of P300 effects across OCD studies is not necessarily contradictory. Rather, it suggests that OCD-related cognitive dysfunction may affect the efficiency, timing, and task-specific deployment of late evaluative processing rather than producing a stable global reduction in P300 amplitude.

A first mechanism concerns inefficient allocation of attentional resources during target detection and context updating. In classical oddball paradigms, P300 amplitude is strongly influenced by target probability, task relevance, and the amount of processing capacity allocated to the target stimulus, whereas P300 latency is more closely related to the time required for stimulus evaluation or categorisation [67,69,72]. This distinction is important for interpreting the reviewed OCD findings. When OCD patients show reduced P300 amplitude with preserved latency, the mechanism is unlikely to be a simple slowing of stimulus classification. Instead, it suggests that the late neural response to task-relevant target stimuli is weaker, less sustained, or less efficiently coordinated across fronto-central and parietal networks. In cognitive terms, this may reflect reduced efficiency in updating the internal model of the task after detecting a relevant event, rather than a primary sensory or perceptual deficit. The associations between lower P300 amplitude and poorer performance on executive tasks such as Stroop interference or Trail-Making Test Part B further suggest that attenuated P300 activity may index reduced availability of late attentional resources when participants must maintain task goals, inhibit competing responses, or flexibly shift between cognitive sets.

This interpretation also helps explain why behavioural impairment was not always present. P300 abnormalities may represent a compensatory or subclinical inefficiency that becomes behaviourally visible only when task demands exceed available control resources. In simple oddball tasks, patients may still detect targets accurately, but reduced or shortened P300 activity may indicate that the neural process supporting stimulus evaluation and context updating is less robust. A shortened P300 duration may therefore reflect premature termination of late evaluative processing or reduced maintenance of the updated stimulus representation. Conversely, preserved amplitude in some studies does not necessarily imply normal cognition, because latency, topography, duration, and condition-specific modulation may still reveal altered processing. This is consistent with the broader view that P300 amplitude is sensitive to processing capacity and task demands but is not a pure or isolated measure of a single cognitive function [69,70].

A second mechanism involves altered fronto-parietal updating and neuromodulatory gain. Contemporary models propose that P3a and P3b arise from distributed interactions among frontal, temporal, parietal, and hippocampal-related systems, with P3a more strongly associated with orienting to novelty and P3b with target evaluation and context updating [70]. The locus coeruleus–norepinephrine model further proposes that the P3 reflects phasic neuromodulatory responses that increase neural gain for motivationally or behaviourally significant events [73,74]. From this perspective, reduced P300 amplitude in OCD may reflect insufficient phasic enhancement of task-relevant information, whereas enhanced P3b in emotionally salient tasks may reflect excessive gain assigned to internally threatening or personally meaningful stimuli. One possible interpretation of the task-dependent direction of effects is that the relevant system may be recruited less strongly during neutral target detection or executive set shifting but more strongly during emotionally salient or symptom-relevant processing. This interpretation remains provisional because the included studies did not directly measure neuromodulatory gain.

A third mechanism concerns late inhibitory control. In Go/NoGo and stop-signal paradigms, the P3 is commonly interpreted as indexing later stages of inhibition, including evaluation of the need to withhold a response, implementation of stopping, or monitoring of whether a stimulus classification has been successfully transformed into an appropriate action rule [75,76,77]. The reviewed OCD findings are broadly consistent with this framework, as P3 abnormalities were most evident in paradigms imposing greater late evaluative or inhibitory demands. A longer NoGo-P3 latency in OCD, even without reduced accuracy, may indicate that the classification of a NoGo stimulus and the updating of the action plan require more time. Reduced Stop-P3 amplitude during successful inhibition in some OCD subgroups may indicate weaker late-stage implementation or evaluation of motor suppression. However, the preservation of Stop-P3 in other studies suggests that inhibitory dysfunction in OCD is not always located at the P3 stage. In some paradigms, earlier components such as N2 may better capture conflict detection or initial stopping processes, whereas P3 abnormalities emerge only when inhibition depends on sustained evaluation, response-rule updating, or allocation of additional control resources.

A fourth mechanism involves impaired use of feedback for adaptive behavioural change. Feedback-related P300 activity is generally associated with the motivational significance, magnitude, expectancy, and behavioural relevance of outcomes, complementing earlier feedback-related components such as the FRN [78,79,80]. In reversal learning and probabilistic feedback tasks, the P300 may therefore reflect not simply the detection of positive or negative feedback, but the degree to which feedback is used to update the current task model and guide future behaviour. The reviewed finding that feedback-related P300 modulation was linked to error frequency suggests that altered P300 in OCD may partly reflect the downstream consequences of inefficient learning rather than a primary abnormality in feedback evaluation. This association is compatible with the possibility that feedback-related P300 activity indexes inefficient conversion of outcome information into an updated action policy. However, the available cross-sectional findings do not establish whether altered P300 activity contributes to behavioural updating difficulties or arises as a consequence of them. This is particularly relevant to OCD, where cognitive rigidity, repetitive checking, and difficulty disengaging from previously reinforced rules may depend on abnormal interactions between performance monitoring, reward learning, and context updating.

A fifth mechanism concerns excessive late processing of emotionally salient or personally threatening information. Emotional Stroop and threat-bias studies show that affectively significant stimuli can capture attentional resources and modulate later ERP positivities, especially when emotional meaning competes with task goals [81,82,83]. In this context, enhanced P3b in OCD should not be interpreted as better cognitive processing. Rather, it may reflect excessive allocation of late evaluative resources to emotionally or symptom-relevant material. This could interfere with efficient task performance by increasing the salience of irrelevant semantic or affective content. Therefore, larger P3b amplitudes in emotional Stroop conditions may represent over-engagement of evaluative processing, not enhanced control. This mechanism is compatible with clinical features of OCD in which intrusive threat representations, uncertainty, and internally generated concerns compete with externally defined task rules.

The reviewed topographic findings also suggest that P300 abnormalities in OCD may involve altered network configuration rather than only amplitude reduction. A reduced parietal-greater-than-frontal distribution or atypical frontal involvement may indicate a shift in the balance between posterior context-updating processes and frontal control processes. Because P3 scalp distribution is influenced by task modality, target discrimination difficulty, response demands, and the relative contribution of frontal and parietal sources, abnormal topography may reflect altered recruitment of the broader attention-control network rather than a localised deficit [70,71]. Importantly, similar P3 topographic abnormalities in other anxiety-related groups suggest that some P300 effects may be transdiagnostic. Thus, P300 may capture a broader anxiety-related disturbance in late evaluative processing, arousal regulation, or attentional updating, while only some task-specific effects may be directly relevant to OCD-related cognitive dysfunction.

The risk-of-bias assessment further qualifies the interpretation of P3/P300 abnormalities. Among the studies with an overall moderate risk of bias, study [29] identified reduced P300 amplitude in paediatric OCD but did not find a direct relationship between P300 measures and response-time variability, while study [23] found impaired stop-signal performance without establishing a direct association between Stop-P3 and inhibitory performance. In contrast, several studies reporting associations between P300 and Stroop interference, Trail-Making Test performance, feedback-guided behaviour, or executive functioning were judged to have a serious overall risk of bias [21,27,30,31,47,53,55]. These studies remain informative, but their estimates were more vulnerable to residual confounding, small samples, multiple electrophysiological comparisons, and exploratory correlations. Consequently, the risk-informed synthesis provides stronger support for task-dependent group differences in late attentional and evaluative processing than for P300 as a stable individual-level marker of cognitive impairment in OCD.

### 4.2. Mechanisms of P2/P200

The available P2/P200 findings suggest that early-to-middle stimulus evaluation is altered in OCD, but in a more context-dependent manner than later ERP components such as P300. Mechanistically, this is important because P2/P200 is not usually interpreted as a pure index of executive control or conscious context updating. Instead, it is thought to occupy an intermediate position between early sensory encoding and later decisional or working-memory updating processes. Across auditory and visual ERP paradigms, P2/P200 has been linked to stimulus classification, attentional allocation, task relevance evaluation, perceptual learning, salience detection, and the early matching of incoming sensory information against currently active task templates [84,85,86,87]. Therefore, P2/P200 abnormalities in OCD are unlikely to reflect a single cognitive deficit. They more probably indicate that the brain assigns abnormal early significance to certain classes of stimuli, especially when they are task-relevant, repeated, salient, or linked to vulnerability-related processing biases.

The increased P200 pattern observed in the auditory oddball/endophenotype context may be interpreted as evidence for heightened early evaluative engagement rather than impaired perception per se. In oddball paradigms, the nervous system must rapidly separate frequent, expected stimuli from infrequent or task-relevant events. Although P300 is classically associated with later context updating, P2/P200 occurs earlier and may reflect the first stage at which the stimulus is categorised as potentially relevant for further processing. Potts showed that frontal P2a is especially sensitive to task relevance rather than merely to physical rarity, suggesting that P2/P200 can index the early appraisal of whether a stimulus matches the current attentional set [85]. In this framework, larger P200 amplitudes in individuals with OCD-related familial vulnerability may reflect an increased tendency to allocate early processing resources to stimuli that healthy controls process more efficiently or dismiss more automatically. Such a mechanism would fit broader models of OCD-related cognition in which excessive significance is assigned to internal or external events before later executive systems determine whether the event truly requires action.

This interpretation is also consistent with auditory-learning studies showing that P2 can increase when repeated exposure or training strengthens auditory object representations. Tremblay and colleagues proposed that auditory P2 participates in stimulus familiarisation and the formation of stable perceptual representations, while Ross and Tremblay reported that stimulus experience modifies auditory N1 and P2 responses in partially dissociable ways [87,88]. Applied to OCD, an increased P200 in an auditory oddball paradigm may therefore indicate not simply “more attention,” but altered early stabilisation of stimulus representations. The brain may be treating ordinary task stimuli as more salient, more behaviourally meaningful, or more in need of evaluative confirmation. This could help explain why P200 contributed to distinguishing patients and unaffected siblings from controls even when it was not directly correlated with a specific neuropsychological score. In this sense, P200 may behave more like a vulnerability-sensitive marker of early evaluative style than a direct marker of executive impairment.

The reduced P2 pattern in the visual repetition-priming context points to a different mechanism. Repetition paradigms engage neural processes related to stimulus familiarity, facilitation, repetition suppression, and the rapid reactivation of existing perceptual or response representations. ERP studies of repetition priming show that repeated stimuli can modulate components in the P2/N2/N400 time range, with the direction of the effect depending on stimulus type, task demands, awareness, and whether repetition produces enhanced access or reduced neural effort [89,90,91]. Thus, a reduced visual P2 to repeated Go targets in OCD does not necessarily mean weaker perception. It may instead reflect abnormal efficiency or premature automatisation of repeated stimulus processing. If a repeated task-relevant stimulus is processed with less early evaluative recruitment, behaviour may become faster, but also more rigidly driven by repetition.

This mechanism is particularly relevant to repetitive cognition and checking-related behaviour. In healthy cognition, repetition can be adaptive because it reduces processing demands and facilitates rapid responses. However, when repetition-related facilitation becomes excessive or poorly regulated, repeated stimuli or action tendencies may gain abnormal behavioural momentum. A reduced P2 response occurring alongside exaggerated reaction-time facilitation may therefore indicate that repeated task-relevant stimuli are being processed through an over-facilitated early pathway, with insufficient evaluative reappraisal before response activation. This could contribute to a cognitive style in which repeated stimuli, thoughts, or action contexts are rapidly reactivated but not adequately reclassified as already processed, irrelevant, or safe. Such a mechanism would be compatible with compulsive checking, where repetition does not necessarily produce subjective closure despite repeated exposure to the same information.

The opposite direction of P2/P200 abnormality across paradigms is therefore not necessarily contradictory. Rather, it suggests that P2/P200 indexes different subcomponents of early evaluation depending on task structure. In an auditory oddball paradigm, increased P200 may reflect excessive early assignment of relevance or enhanced perceptual-template engagement. In a visual repetition-priming paradigm, reduced P2 may reflect abnormal facilitation, reduced need for evaluative processing, or premature repetition-based processing of task-relevant stimuli. This distinction is consistent with the broader ERP literature, where P2/P200 is known to vary with modality, attention, salience, learning, and repetition, and is less functionally specific than components such as N2, P3, ERN, or FRN [84,87,90,92].

From a systems perspective, P2/P200 abnormalities may reflect altered communication between sensory cortices and higher-order attentional-control systems. Auditory P2 has been associated with auditory cortical plasticity and stimulus representation, while visual P2/P2a has been linked to frontal and posterior mechanisms of task relevance evaluation [87,88,92]. In OCD, dysfunction may therefore arise not only at the level of late executive control, but also at the earlier stage where sensory events are tagged as relevant, familiar, repeated, or requiring further processing. If this early tagging process is biassed, later cognitive systems may receive distorted input: some ordinary events may be over-prioritised, whereas repeated events may become over-facilitated without adequate updating of their meaning.

Importantly, the current evidence does not support P2/P200 as a robust standalone marker of cognitive dysfunction in OCD. The available studies do not show strong direct P2–behaviour correlations, and the direction of abnormality differs across auditory oddball and visual repetition-priming tasks. Therefore, P2/P200 should not be interpreted in the same way as later components more directly associated with executive monitoring, response inhibition, or working-memory updating. Its mechanistic value lies elsewhere: it may reveal abnormalities in the early evaluative interface between perception and cognition. In this interface, incoming stimuli are not merely detected, but rapidly assigned task relevance, familiarity, salience, and potential need for action. OCD-related dysfunction may partly emerge from biases at this stage, especially when early evaluative abnormalities interact with later deficits in inhibitory control, uncertainty monitoring, and context updating.

### 4.3. Mechanisms of N1/N100

Mechanistically, the N1/N100 findings suggest that early perceptual–attentional processing is not globally impaired in OCD, but may become abnormal when the stimulus carries high motivational relevance, personal threat value, or repetitive significance. This interpretation is consistent with the broader ERP literature, in which N1/N100 is not usually treated as a pure marker of executive dysfunction, but rather as an early index of sensory analysis, attentional selection, stimulus discrimination, and the allocation of perceptual resources. Auditory N100 has long been linked to the cortical registration and analysis of sound, with contributions from auditory cortical generators and modulation by arousal, attention, and stimulus predictability [93]. Similarly, visual N1 is commonly interpreted as reflecting selective attention and discrimination processes, especially when task demands require distinguishing relevant from irrelevant features or locations [94,95,96]. From this perspective, the inconsistent N1/N100 findings in OCD are theoretically meaningful: they suggest that the earliest stages of stimulus encoding are not uniformly dysfunctional, but are selectively altered when early perceptual analysis is recruited by stimuli that map onto core OCD mechanisms.

This helps explain why N1/N100 did not clearly account for cognitive flexibility deficits in the task-switching/backward-inhibition study. If the central impairment concerns the reactivation of previously abandoned task sets, perseverative response tendencies, or inhibitory control over competing rules, the critical dysfunction may occur downstream from basic stimulus discrimination. In such a context, N1 would be expected to show group differences only if the abnormality already affected the earliest cue- or target-selection stage. The absence of a critical N1 effect therefore suggests that OCD-related inflexibility in this task was not primarily caused by deficient early sensory discrimination, but by later or partly distinct control processes. This is consistent with models in which N1 indexes early selection of perceptual information, whereas later components such as P3, N2/N200, N450, or response-related negativities are more closely tied to context updating, conflict monitoring, response inhibition, and performance evaluation.

The auditory oddball and prepulse-inhibition findings point to a related mechanism. N100 latency differences in patients and unaffected siblings may indicate altered early auditory registration or sensory-attentional timing, but the lack of strong ERP–cognition correlations suggests that this abnormality is not sufficient to explain broad neuropsychological impairment. In sensory-gating paradigms, N100 suppression can reflect the reduced processing of repeated or closely paired auditory stimuli, helping to protect higher-order cognition from redundant input [97]. However, when N1 gating is preserved across groups and does not predict discrimination performance, it is unlikely to represent a disorder-specific mechanism of cognitive dysfunction. Thus, in OCD, auditory N1/N100 abnormalities may reflect a vulnerability-related alteration in early sensory-attentional timing rather than a direct electrophysiological signature of impaired executive function.

The more mechanistically informative findings come from paradigms involving emotional, personally threatening, or repeated stimuli. In the broader affective ERP literature, emotionally significant stimuli can modulate early visual processing because motivationally relevant information receives prioritised perceptual encoding [98,99,100]. In OCD, this principle may be especially important because threat is often idiosyncratic: a stimulus becomes cognitively disruptive not simply because it is negative, but because it is personally linked to contamination, harm, responsibility, checking, symmetry, taboo thoughts, or other obsessional concerns. Therefore, prolonged N1 latency to personally threatening words can be interpreted as evidence that threat-related stimuli require more extended early discrimination. The stimulus may first capture attention rapidly, as reflected by earlier sensory components such as P1, and then continue to demand perceptual analysis at the N1/N100 stage. This creates a temporal cascade in which early attentional capture is followed by slower discrimination, increasing the probability that later interference-control mechanisms will be overloaded.

The repetition-priming findings are also mechanistically important. In healthy cognition, repeated stimuli usually produce more efficient processing, reflected behaviourally in priming and neurophysiologically in reduced neural responses or shorter processing times. Repetition-suppression models propose that repeated input can reduce neural activity through mechanisms such as sharpening of stimulus representations, facilitation of processing, or reduced prediction error [101,102]. The abnormal N1 latency pattern in OCD suggests that repeated stimuli may not become efficiently processed in the usual way. Instead of repetition producing faster early discrimination, repeated information may remain salient, attention-demanding, or incompletely resolved. This mechanism fits well with the phenomenology of OCD, where intrusive thoughts, doubts, or sensory impressions often fail to lose relevance despite repetition. At the electrophysiological level, abnormal N1 modulation may therefore reflect inefficient early habituation or atypical persistence of perceptual representations, which could contribute to perseverative cognition and difficulty disengaging from recurring stimuli or mental content.

Therefore, the mechanistic role of N1/N100 in OCD is best understood as an early amplification or prolongation of perceptual–attentional processing under conditions of motivational relevance. This does not replace later-stage models of OCD involving conflict monitoring, error processing, or fronto-striatal control dysfunction, but complements them. N1/N100 abnormalities may represent the earliest point at which personally meaningful stimuli become overrepresented in the cognitive system. Once this early bias occurs, later systems responsible for inhibition, updating, and performance monitoring may be forced to operate on already-prioritised threat or repetition signals. In this way, N1/N100 may contribute indirectly to cognitive dysfunction by shaping the input that later executive systems must regulate.

### 4.4. Mechanisms of N2/N200

The reviewed findings suggest that N2/N200 abnormalities in OCD should be interpreted not as a uniform increase or decrease in early control activity, but as a task-dependent disturbance in the recruitment, timing, and functional organisation of early cognitive-control mechanisms. In the broader ERP literature, the N2 is usually described as a negative deflection peaking approximately 200–350 ms after stimulus onset, with partially separable subcomponents related to mismatch detection, novelty, attentional selection, conflict monitoring, and motor-control demands [103]. This multiplicity is crucial for interpreting the OCD findings, because the same scalp component may index different operations depending on whether the task requires withholding a prepotent response, cancelling an already initiated response, detecting stimulus conflict, resolving competing response tendencies, or adapting control under emotionally or socially salient conditions.

The most consistent mechanistic implication of the available OCD studies is that N2/N200 reflects an abnormal early control stage rather than a purely late evaluative deficit. In both inhibitory-control studies, later P3 indices were relatively preserved, whereas N2 abnormalities were prominent. This dissociation suggests that OCD-related cognitive dysfunction may arise before the stage of full stimulus evaluation or conscious updating, at the point where the system detects the need for control and begins to configure an appropriate inhibitory response. In cognitive-control models, frontocentral N2 activity has often been linked to medial frontal/anterior cingulate mechanisms involved in detecting conflict between competing representations and signalling the need for increased top-down control [104]. Therefore, reduced N2 amplitudes in the Go/NoGo paradigm may indicate weakened or inefficient early conflict-control recruitment, even when overt performance remains intact. The absence of behavioural impairment in that task does not necessarily imply normal control; instead, it may indicate that patients can preserve accuracy through compensatory strategies, slower downstream processing, or greater reliance on later or non-frontal mechanisms.

The altered topography observed in the Go/NoGo study strengthens this interpretation. Healthy controls showed the expected frontal/frontocentral N2 distribution, whereas OCD patients showed a more posterior maximum. Mechanistically, this posterior shift may reflect reduced engagement of canonical frontomedial control generators and a relative redistribution toward sensorimotor or posterior attentional systems. Such a pattern would be consistent with the idea that patients are not simply producing a smaller version of the normal inhibitory-control response, but are organising early control differently. In this sense, the reduced N2 may indicate diminished efficiency of frontal conflict detection or action-restraint mechanisms, while preserved behaviour may reflect compensation by other neural processes that are not captured by the N2 alone.

The apparently opposite finding from the stop-signal study can be reconciled by distinguishing action restraint from action cancellation. Go/NoGo tasks primarily require restraint: the participant must withhold a response before it is executed. Stop-signal tasks require cancellation: a response has already been initiated and must be interrupted after the stop signal appears. These two forms of inhibition are related but not identical, and they engage partly different cognitive and neural processes [105,106]. In this framework, reduced Go/NoGo N2 in OCD may reflect under-recruitment of early restraint mechanisms, whereas enhanced Stop-N2 may reflect excessive conflict or compensatory recruitment during reactive cancellation. Because the stop-signal task creates a race between an ongoing go process and a later stop process, longer stop-signal reaction times in OCD indicate that the stopping process is less efficient. Larger Stop-N2 amplitudes, especially when N2 is larger for failed than successful inhibition, may therefore index increased conflict between the go and stop processes rather than better inhibition. In other words, the enhanced Stop-N2 may represent a neural marker of effortful but inefficient control.

This interpretation is compatible with broader stop-signal ERP evidence showing that stop signals elicit an N2/P3 complex and that these components can differ between successful and unsuccessful stopping [75]. The preserved Stop-P3 in OCD is also important. If P3 is more closely related to later inhibitory implementation, response evaluation, or the updating of the stop event, then a selective N2 abnormality suggests that the main dysfunction lies in the early detection and mobilisation of control rather than in the later registration of the stop signal. Thus, OCD may involve a mismatch between early conflict signalling and effective behavioural cancellation: the brain detects high conflict, but this detection does not translate into faster stopping.

The divergence between reduced Go/NoGo N2 and enhanced Stop-N2 also argues against treating N2/N200 as a simple biomarker of “inhibition strength.” The broader literature has long debated whether NoGo-N2 reflects inhibition per se or conflict monitoring. Donkers and van Boxtel (2004) [107] argued that N2 in Go/NoGo tasks is better understood as conflict monitoring than as a direct measure of response inhibition, and later work has continued to emphasise that N2 and P3 reflect partly distinct stages of inhibitory processing [20,108]. This distinction is useful for OCD. Reduced N2 may not mean that patients lack inhibitory ability in general; rather, it may mean that early conflict detection or control allocation is insufficient in some contexts. Conversely, enhanced Stop-N2 may not mean superior inhibition; it may indicate that the system is generating a stronger conflict signal because stopping is difficult, delayed, or inefficient.

The task-switching findings further show that N2/N200 is not a universal explanation for cognitive-performance differences in OCD. In the backward-inhibition paradigm, behavioural differences were not mirrored by N2 changes, suggesting that the relevant mechanism occurred earlier in the processing stream, possibly at the level of perceptual gating or task-set selection. This is mechanistically important because it implies that repetitive or recently activated task sets may be handled differently in OCD before the need for N2-indexed conflict monitoring emerges. If irrelevant task-set information is suppressed or filtered at an early perceptual–attentional stage, then later N2 conflict activity may not be strongly engaged. Therefore, some OCD-related cognitive differences may arise from early gating mechanisms rather than from impaired conflict monitoring itself.

The social-hierarchy study adds another layer by showing that N2 abnormalities may be amplified by evaluative context. Reduced frontal N2 under socially inferior or evaluative conditions suggests that cognitive-control dysfunction in OCD may become more pronounced when control must be exerted under perceived threat, inferiority, or social pressure. This fits broader evidence that negative affect and cognitive control converge in dorsal cingulate/anterior midcingulate systems, which integrate aversive salience, performance demands, and action regulation [109]. It is also consistent with ERP work showing that threat and anxiety can modulate N2 activity during conflict monitoring [110]. In OCD, social-evaluative pressure may therefore load the same control network that is already needed for task performance, reducing the efficiency with which early conflict signals are translated into adaptive control. The association between symptom severity and N2 amplitude in the socially threatening condition supports this view: more severe symptoms may be linked to stronger disruption of early control when the task context increases evaluative salience.

The weaker findings from prepulse-inhibition and repetition-priming paradigms also help delimit the role of N2/N200. In the prepulse-inhibition study, behavioural impairment and altered gating were not clearly reflected in N2. This suggests that N2 is not necessarily sensitive to all forms of sensory gating or attentional interference in OCD. Earlier components such as P50 and N1, and later target-related components such as P3, may be more informative when the paradigm primarily probes sensory registration, early auditory gating, or target discrimination. Similarly, the repetition-priming study showed altered N2 topography, but the effect was not specific to OCD and was also observed in panic disorder. This indicates that N2 abnormalities may partly reflect transdiagnostic alterations in frontal monitoring or anxiety-related control allocation rather than OCD-specific cognitive dysfunction.

From a risk-of-bias perspective, the evidence for N2-related inhibitory dysfunction receives important support from study [23], which was classified as having a moderate overall risk. This study found longer stop-signal reaction times and larger frontal Stop-N2 amplitudes in medication-free patients, although it did not demonstrate a direct Stop-N2–behaviour correlation. More complex findings involving OCD subtypes and multiple emotional and inhibitory components, including those reported in study [53], arose from studies at serious overall risk. Thus, altered N2 activity can be regarded as a plausible group-level correlate of increased inhibitory-control demand, but the current evidence does not establish N2 amplitude as an individual predictor of inhibitory impairment.

### 4.5. Mechanisms of ERN

The present synthesis indicates that the error-related negativity provides one of the clearest electrophysiological windows into altered cognitive control in OCD, but not in the simple sense of indexing impaired task performance. Across the reviewed studies, ERN abnormalities were most consistently expressed as exaggerated early neural responses to errors, especially in paediatric OCD and in flanker-type response-conflict paradigms. Mechanistically, this pattern suggests that OCD is characterised by an abnormal increase in the gain of the internal performance-monitoring system. The ERN is classically interpreted as a rapid response-locked signal generated when an executed action deviates from the intended response or when the monitoring system detects conflict between competing response tendencies [111,112,113]. Therefore, the repeatedly enlarged ERN in OCD is best understood as an overactive neural alarm signal for internally detected action errors rather than as a direct electrophysiological correlate of poor behavioural performance.

This interpretation is supported by the frequent dissociation between ERN amplitude and overt task behaviour. In many reviewed studies, OCD patients showed enlarged ERN amplitudes despite normal reaction times, comparable accuracy, normal post-error slowing, or preserved self-correction. Such findings suggest that the cognitive dysfunction captured by the ERN occurs at the level of monitoring and evaluative control, before it necessarily becomes visible in behaviour. In other words, OCD may involve excessive early detection of possible action failure, while downstream behavioural adjustment remains partly intact. This distinction is important because the ERN does not simply measure whether a person performs poorly; rather, it reflects how strongly the medial frontal performance-monitoring system registers the need for control. Neurocognitive models propose that medial frontal structures, particularly the dorsal anterior cingulate cortex and adjacent posterior medial frontal cortex, evaluate conflict, errors, and unfavourable outcomes, then signal other control systems to adjust behaviour [114,115,116]. In OCD, this signalling system appears to be hypersensitive, producing a large internal error signal even when the behavioural consequences of the error are minimal.

The reviewed findings also suggest that enhanced ERN should not be interpreted as a uniformly maladaptive deficit. In several paediatric studies, larger ERN amplitudes were associated with better accuracy or fewer errors. This pattern is compatible with a compensatory monitoring account: stronger early error detection may help some patients maintain accurate performance, particularly in structured tasks with clear correct and incorrect responses. From this perspective, ERN enhancement may represent a costly form of cognitive compensation. The patient performs adequately, but only through excessive recruitment of monitoring mechanisms. Such over-monitoring could preserve accuracy in simple laboratory tasks while contributing clinically to doubt, checking, and repeated evaluation of action outcomes in everyday life. Mechanistically, the same system that helps detect errors may become pathologically over-calibrated, treating ordinary response slips or uncertainty as disproportionately salient signals requiring further control.

The task-dependence of the ERN findings is particularly informative. ERN enhancement was most reliable in tasks involving clear, immediate, internally detected motor errors, such as flanker paradigms. In contrast, probabilistic learning, reversal learning, recognition-memory paradigms, affective error-processing tasks, and anti-saccade paradigms produced more variable findings, including normal, reduced, or context-dependent ERN responses. This suggests that OCD is not characterised by a global increase in all error-related neural activity. Rather, the disorder may involve an imbalance between internal action-error monitoring and flexible outcome-based learning. In reinforcement-learning models, the ERN has been linked to negative prediction-error signals conveyed through dopamine-dependent systems to the anterior cingulate cortex, where they can guide future behavioural adaptation [117,118,119]. The present OCD evidence fits a model in which clear self-generated response errors are over-signalled, whereas ambiguous errors that depend on probabilistic feedback, changing contingencies, or uncertainty are less efficiently integrated into adaptive learning. This mechanism may help explain why OCD patients can show excessive sensitivity to obvious mistakes while still struggling with flexible updating, uncertainty resolution, and disengagement from maladaptive response strategies.

Time–frequency findings further refine this mechanism. The ERN is not only a time-domain ERP deflection; it is closely related to mid-frontal theta activity, which is thought to coordinate cognitive control across distributed neural systems. Frontal theta has been proposed as a mechanism through which the brain detects the need for control and communicates this demand to task-relevant networks [120]. In the reviewed OCD studies, increased error-related theta power and its association with ERN amplitude suggest that enlarged ERN may partly reflect excessive mid-frontal theta recruitment. However, theta enhancement did not consistently explain broader planning deficits or post-error behavioural adjustment. This implies that the abnormality may lie not only in generating an error signal, but also in translating that signal into flexible, context-appropriate control. OCD may therefore involve hyperactive monitoring without proportionally efficient adaptive regulation.

The developmental pattern also has mechanistic significance. The most robust ERN enlargement appeared in paediatric OCD and in familial-risk designs, suggesting that exaggerated error monitoring may emerge early and may index vulnerability rather than merely illness chronicity. In typical development, the ERN strengthens across childhood and adolescence as performance-monitoring systems mature [121,122]. OCD may involve an atypical developmental calibration of this system, producing an overly strong internal error signal during a period when cognitive control, affective evaluation, and habit regulation are still maturing. This could make children and adolescents especially prone to interpreting errors, uncertainty, or incomplete actions as highly salient. Over time, such heightened monitoring may interact with compulsive behaviours: checking, repetition, and reassurance seeking may temporarily reduce the aversive signal generated by perceived error or incompleteness, thereby reinforcing the compulsive cycle.

The affective meaning of errors is another important mechanism. Experimental work outside OCD shows that ERN amplitude is modulated by the motivational significance of errors and is often enhanced in anxiety, especially anxious apprehension or worry [123,124,125]. This broader literature helps explain why ERN enhancement in OCD is not disorder-specific. The enlarged ERN may reflect a transdiagnostic mechanism of heightened threat value assigned to errors. However, in OCD, this mechanism may be expressed through disorder-relevant cognitive themes: responsibility, harm avoidance, intolerance of uncertainty, and fear of incomplete or incorrect action. Thus, the ERN may represent the neural expression of an error-as-threat processing style. The error is not merely registered as incorrect; it is treated as motivationally urgent and potentially dangerous.

This mechanism also explains why ERN amplitude is not consistently related to symptom severity or behavioural impairment. A heightened ERN may reflect a relatively stable monitoring style or vulnerability marker rather than a state marker of current clinical intensity. At the same time, its functional consequences likely depend on task context, developmental stage, comorbidity, and the balance between monitoring and adaptive control. When the task is simple and errors are explicit, increased ERN may support accuracy. When the environment is uncertain, feedback is probabilistic, or flexible updating is required, the same overactive monitoring system may become inefficient, rigid, or poorly integrated with reinforcement-learning mechanisms. Therefore, the ERN should be interpreted not as a direct biomarker of cognitive impairment, but as a marker of altered control allocation: too much neural weight is assigned to the detection of error, while the subsequent use of that signal for adaptive behavioural change may be inconsistent.

The ERN literature was comparatively strengthened by the presence of several studies with a moderate overall risk of bias [32,34,42,45,50]. These studies converged on altered or enhanced error-related activity, but they did not show a uniform relationship with cognitive performance. ERN amplitude was unrelated to error frequency or post-error adjustment in studies [32,42]; its association with post-error slowing in study [34] was not specific to OCD; study [45] showed different behavioural associations for ERN/theta and beta activity; and the large paediatric study [50] provided the clearest evidence that both early error detection and later error evaluation independently predicted accuracy. Studies at serious risk frequently reported a similar ERN enlargement, but their interpretation was more vulnerable to clinical confounding, participant exclusions based on error counts, and selective analysis of multiple ERP measures.

Accordingly, the risk-of-bias-stratified evidence supports altered performance monitoring as one of the more reproducible electrophysiological findings in OCD. However, confidence is lower in the stronger claim that ERN magnitude directly indexes the severity of cognitive dysfunction, is specific to OCD, or consistently predicts post-error behavioural adaptation.

### 4.6. Mechanisms of FRN

The present findings suggest that feedback-related negativity abnormalities in OCD should not be interpreted as a simple electrophysiological marker of generalised hypersensitivity to negative outcomes. Rather, the pattern across reversal learning, probabilistic reinforcement learning, and Iowa Gambling Task paradigms indicates a context-dependent disturbance in the transformation of external feedback into adaptive behavioural updating. This distinction is important because the FRN has long been linked to medial-frontal performance monitoring, particularly activity in or near the anterior cingulate cortex and adjacent posterior medial frontal regions, but its functional meaning has shifted from a purely “negative feedback detector” toward a broader index of reinforcement-learning signals, reward prediction error, action–outcome evaluation, and reward positivity modulation [117,118,126,127,128,129]. Within this framework, the FRN findings in OCD point to a dysregulated feedback-learning system in which the balance between negative outcome detection, reward stabilisation, and behavioural flexibility is altered.

A first mechanism concerns impaired use of negative feedback under uncertainty. In the reversal-learning study, OCD patients showed more reversal and exploration errors, but the reduced FRN was specific to exploration negative feedback rather than reversal negative feedback. This suggests that the abnormality was not simply a failure to register that a previously rewarded response had become wrong. Instead, the deficit emerged when participants had to use feedback during an uncertain search phase, when the correct alternative was not yet known, and the participant needed to integrate external feedback into an evolving action-value representation. Classical reinforcement-learning models propose that unexpected unfavourable outcomes produce negative prediction-error signals that are conveyed through dopaminergic and medial-frontal systems and used to update future behaviour [117,118]. Therefore, a reduced FRN during exploration may indicate that OCD patients generate a weaker or less behaviourally useful neural signal when feedback should guide hypothesis testing. Mechanistically, this could produce exactly the behavioural profile observed: repeated selection of disconfirmed alternatives, inefficient switching, and failure to converge on the newly correct response despite preserved reaction speed.

This mechanism also helps reconcile FRN findings with the broader ERP literature on internal versus external error monitoring. OCD is often associated with enhanced internally generated error signals, especially ERN-like activity after the individual’s own mistakes, yet the present FRN findings show that externally delivered feedback may be processed inefficiently when it must be used to support learning. In other words, OCD may involve an imbalance between excessive internal performance monitoring and weak external feedback integration. The individual may detect that “something is wrong,” or experience heightened uncertainty, but the feedback signal does not efficiently update action values or reduce uncertainty. This interpretation is compatible with performance-monitoring models in which medial-frontal signals must be translated into adaptive control adjustments through distributed prefrontal, striatal, and motor systems [116,120]. If this translation is inefficient, stronger monitoring does not necessarily improve cognition; it may instead amplify doubt, checking, and maladaptive repetition.

A second mechanism concerns abnormal reward-feedback processing. In the probabilistic learning study, OCD patients did not show exaggerated loss-related FRN responses. Instead, their FRN/RewP complex was more positive or attenuated, particularly because win feedback elicited abnormally positive responses during active learning, while loss responses were not clearly abnormal. Behaviourally, these patients showed poorer learning of stimulus–reward contingencies and more shifting after wins, suggesting that positive feedback failed to stabilise future choices. This is mechanistically important because contemporary accounts argue that the FRN difference wave may reflect not only increased negativity to unfavourable outcomes but also a reward positivity elicited by favourable outcomes [129]. From this perspective, abnormal responses to wins may reflect disturbed coding of positive prediction errors, impaired reward expectancy formation, or unstable representation of action–reward contingencies. In healthy reinforcement learning, positive feedback should increase confidence in the selected response and reduce unnecessary switching. However, in OCD, win feedback may be processed as insufficiently reliable or insufficiently stabilising, causing the person to continue sampling alternatives despite receiving favourable outcomes.

This reward-stabilisation deficit may be especially relevant to cognitive inflexibility in OCD. Probabilistic learning requires the nervous system to tolerate occasional misleading feedback while extracting the dominant reinforcement structure across trials. ERP studies in healthy participants show that FRN/RewP amplitudes are modulated by reward expectancy and prediction error, and that learning-related changes in reward expectancy are reflected in the feedback-locked medial-frontal signal [128,130]. If OCD patients show abnormal reward-feedback coding, they may fail to build stable expectations even when contingencies are relatively easy. This would explain why deficits may appear not only after punishments or losses, but also after rewards: the core dysfunction is not only excessive punishment sensitivity, but impaired calibration of confidence after feedback.

A third mechanism is excessive loss–win differentiation during risky decision-making. In both IGT-based studies, OCD or high obsessive–compulsive traits were associated with poorer decision-making and larger loss-minus-win FRN difference waves. Importantly, larger FRN differentiation was associated with worse IGT performance. This shows that enhanced neural discrimination between unfavourable and favourable outcomes is not necessarily adaptive. In a risky decision-making context, excessive loss-related medial-frontal signalling may increase the salience of negative outcomes without supporting strategic integration across trials. The IGT requires gradual learning from cumulative feedback, integration of reward and punishment frequency, and suppression of immediately tempting but long-term disadvantageous choices. A larger dFRN may therefore reflect over-weighting of immediate unfavourable feedback, heightened affective salience of losses, or inefficient updating of long-term value representations. Rather than guiding flexible adaptation, the signal may become noisy, over-reactive, or disconnected from the broader valuation system needed for advantageous choice.

The source-localisation findings in the subclinical obsessive–compulsive group further suggest that this mechanism may involve abnormal interaction between anterior prefrontal/orbitofrontal regions and dorsal anterior cingulate cortex. The orbitofrontal cortex is central for representing outcome value and updating stimulus–reward contingencies, whereas the dorsal anterior cingulate and adjacent medial-frontal regions are involved in performance monitoring, prediction error, and the allocation of control. If anterior prefrontal/orbitofrontal activity during loss feedback is increased but its functional relationship with dorsal anterior cingulate activity is disrupted, the person may detect losses strongly but fail to use them to optimise behaviour. This would produce the paradox observed in the IGT studies: stronger neural loss monitoring but poorer decision-making. Thus, the problem is not insufficient sensitivity to bad outcomes; rather, it may be excessive local outcome salience combined with impaired network-level integration.

This interpretation also clarifies the relationship between FRN and cognitive dysfunction. Across the reviewed studies, FRN measures were associated with errors, learning performance, shifting behaviour, and decision-making outcomes. These associations make a purely incidental interpretation less likely, but they do not establish that altered FRN activity caused the behavioural difficulties. However, the direction of the association depended on task demands. Reduced FRN during exploratory negative feedback may indicate insufficient prediction-error signalling for behavioural updating. Abnormal win-related RewP activity may indicate impaired reward learning and reduced confidence in positive feedback. Enlarged loss-minus-win FRN during IGT performance may indicate excessive unfavourable-outcome monitoring that interferes with long-term adaptive choice. FRN abnormalities in OCD may therefore be interpreted as candidate correlates of altered feedback utilisation rather than solely as indicators of heightened sensitivity to negative feedback.

In mechanistic terms, OCD-related cognitive dysfunction may involve disrupted coordination between dopaminergic reinforcement-learning signals, medial-frontal performance-monitoring systems, and orbitofrontal/prefrontal value-updating networks. Healthy feedback learning requires these systems to generate prediction-error signals, update action values, stabilise rewarded behaviour, and flexibly shift away from disadvantageous options. The present FRN evidence is consistent with possible disruption at several stages of this sequence, although the available designs cannot establish which stage is primary or determine the direction of the relationship. Feedback may be detected but not weighted appropriately; rewards may be registered but not used to stabilise action; losses may be strongly differentiated but not integrated into advantageous long-term decision-making. This electrophysiological pattern offers a provisional framework for understanding cognitive features such as uncertainty, indecisiveness, perseveration, impaired reversal learning, and maladaptive decision-making. However, direct relationships with these clinical phenomena were not tested consistently, and the proposed link should therefore be regarded as theoretical.

The feedback-related evidence should be interpreted more cautiously than the ERN evidence when methodological quality is considered. The principal studies of clinically diagnosed OCD examining FRN, reward positivity, reversal learning, or Iowa Gambling Task performance were classified as having a serious overall risk of bias [30,49,56]. Study [57], which had a moderate overall risk and demonstrated a relationship between larger FRN responses and poorer decision-making, involved participants with subclinical obsessive–compulsive traits rather than a clinical OCD sample. Therefore, the available findings support the plausibility of altered feedback evaluation and reinforcement learning, but the clinical specificity and generalisability of the FRN mechanism remain preliminary.

### 4.7. Mechanisms of CNV

Mechanistically, the available evidence suggests that CNV should be interpreted with considerable caution as a marker of cognitive dysfunction in OCD. The single available study measured CNV only as a secondary physiological variable within an auditory prepulse-inhibition/discrimination paradigm, and CNV did not emerge as the main component distinguishing OCD patients from healthy controls. This is important because CNV is not a unitary index of “cognitive dysfunction” but a slow, multi-componential anticipatory potential generated between a warning stimulus and an imperative stimulus. Since the original description of CNV as an electrophysiological sign of sensorimotor association and expectancy, later ERP work has shown that CNV reflects the integration of temporal expectation, orienting, anticipatory attention, stimulus–response preparation, motor readiness, and decision-related processes rather than a single cognitive operation [131,132,133,134]. The absence of a clear CNV abnormality may indicate that the particular measure or paradigm was not sensitive to an OCD-related difference; alternatively, anticipatory processing may have been relatively preserved in this sample.

A central methodological issue is that CNV was assessed in the 500 ms prepulse–target interval. Classical CNV paradigms often use longer S1–S2 intervals, allowing separation of an earlier orienting component and a later terminal component. The early CNV has been linked more strongly to orienting and expectancy after the warning stimulus, whereas the late CNV is more closely related to motor preparation and readiness for the imperative stimulus [135,136,137]. In the present OCD evidence, the 500 ms condition allowed some anticipatory activity to develop, but it may have compressed several processes into a short preparatory window. Thus, any subtle abnormality in the temporal deployment of attention, response readiness, or decision threshold could have been diluted within a broad CNV estimate at S2 onset. This may explain why CNV was less informative than later target-related components.

From a mechanistic perspective, the task required participants to use the prepulse as a temporally predictive cue while preparing to discriminate the second auditory stimulus and execute a speeded response only to targets. In healthy cognition, this kind of warned discrimination should allow the nervous system to reduce uncertainty before S2, lower perceptual and response thresholds, and prepare the appropriate stimulus–response mapping. CNV source-localisation and EEG–fMRI studies have linked these anticipatory processes to a distributed fronto-cingulo-motor network, including supplementary motor area, anterior cingulate cortex, premotor/motor regions, thalamic contributions, and prefrontal control regions [138,139]. If this system were dysfunctional in OCD, one would expect inefficient temporal orienting and motor preparation: slower responses, increased omissions, and a more conservative response criterion under conditions of sensory interference. The behavioural findings in the study are broadly compatible with this interpretation, because OCD patients showed slower responses, more omission errors, and a conservative response style. However, because CNV itself was not clearly abnormal or specifically associated with these behavioural measures, the data do not justify treating CNV as the direct electrophysiological mechanism of these deficits.

The more plausible interpretation is that the task revealed a broader disturbance in the transition from early sensory filtering to later target evaluation and response selection. Prepulse paradigms are designed to test how a weak leading stimulus modulates processing of a subsequent stimulus. Work on PPI and P50/N100/P200 gating suggests that early sensory and sensorimotor gating can protect higher-order cognition by reducing interference from irrelevant or redundant stimulation, thereby supporting attention, response bias, and working-memory operations [97,140,141,142]. However, in the OCD study, early physiological gating measures were not the strongest predictors of discrimination performance. Instead, behavioural RT slowing induced by the short prepulse interval was more closely related to poorer perceptual sensitivity and a conservative response criterion. This pattern suggests that cognitive inefficiency may arise less from a simple failure of early sensory gating and more from difficulty translating temporally crowded sensory input into efficient perceptual decisions and action selection.

This interpretation is reinforced by the stronger role of P3-related processing in the study. P3 amplitude is widely interpreted as an index of attentional resource allocation, context updating, and the processing capacity devoted to task-relevant stimulus evaluation, while P3 latency is often linked to stimulus classification speed rather than motor execution alone [69,70]. In an auditory discrimination task, target–non-target P3 differentiation therefore provides a more direct marker of whether the participant successfully categorised the stimulus and updated the task context. The finding that P3 differentiation was more closely related to omission errors and perceptual sensitivity than CNV suggests that the principal dysfunction may occur at the stage of target evaluation rather than during anticipatory preparation itself. In this framework, OCD-related slowing and conservative responding may reflect inefficient accumulation or evaluation of evidence after S2, especially when the preceding prepulse creates sensory interference.

Overall, the CNV evidence does not currently support CNV as a distinct electrophysiological biomarker of cognitive dysfunction in OCD. Instead, it indicates that anticipatory preparation remains an under-explored mechanism. The available study shows that OCD patients may behave less efficiently in a temporally demanding auditory discrimination task, but the electrophysiological signature of this inefficiency was more evident in sensory-gating and target-evaluation processes than in CNV. A cautious mechanistic model is therefore that OCD-related cognitive dysfunction in this paradigm involves impaired coordination between early sensory filtering, anticipatory set formation, and later stimulus classification. CNV may still be relevant to this model, because it indexes the preparatory bridge between warning cues and goal-directed action; however, the existing evidence is insufficient to determine whether this bridge is genuinely impaired in OCD. Future ERP studies should use CNV-focused paradigms with longer and parametrically varied fore-periods, separate early and late CNV components, examine frontocentral topography and source-level generators, and directly correlate CNV indices with omission errors, reaction-time variability, perceptual sensitivity, response criterion, and executive-control measures. Only such designs can determine whether CNV abnormalities reflect impaired temporal expectancy, reduced proactive control, abnormal motor preparation, or compensatory over-preparation in OCD.

### 4.8. Mechanisms of LRP-r

The LRP-r findings suggest that cognitive dysfunction in OCD may extend into the final stages by which decisions are translated into overt motor responses. The LRP is generally interpreted as an electrophysiological marker of lateralised central motor activation, reflecting the preferential preparation of the response hand before or around response execution [143,144,145,146]. In this framework, the enlarged LRP-r observed in OCD can be interpreted as evidence of abnormal motor-channel activation once a response has already been selected. Rather than indicating a purely perceptual or decisional deficit, this pattern points to altered late-stage response implementation, in which the motor system may require stronger or more sustained lateralised activation to execute, stabilise, or inhibit the selected action.

Mechanistically, this finding fits with models in which response preparation is not a discrete endpoint of cognition, but a continuous process through which partial perceptual and decisional information is progressively transmitted to the motor system [143,144,145]. Under normal conditions, LRP activity reflects the gradual accumulation of evidence toward one response channel and the suppression of competing response tendencies. In OCD, the larger LRP-r may therefore reflect inefficient gating between decision-related processes and motor output. The selected response may reach the motor system with excessive activation, prolonged checking, or reduced automaticity, suggesting that even after a decision has been made, its implementation remains over-controlled. This interpretation is consistent with the broader view that cognitive dysfunction in OCD is not limited to early conflict detection or error monitoring, but also involves the motor implementation stage of action control.

The response-locked nature of the abnormality is particularly important. Stimulus-locked LRP measures are typically more informative about the timing of response selection and premotor preparation, whereas response-locked LRP activity reflects processes closer to response execution, including the final buildup of motor activation before overt movement [145,146,147,148]. Thus, the enlarged LRP-r in OCD is unlikely to reflect only slower stimulus evaluation or uncertainty about the perceptual decision. Instead, it suggests that the abnormality occurs at the interface between selected action and motor execution. This may represent a neurophysiological mechanism through which excessive control, doubt, or action-monitoring demands interfere with fluent responding.

Evidence from response-inhibition research further supports this interpretation. Stop-signal and Go/NoGo ERP studies using LRP measures show that prepared responses can still be inhibited after central motor activation has begun, and that successful inhibition depends on interactions between response activation and top-down control mechanisms [149,150,151,152]. From this perspective, increased LRP-r amplitude in OCD may reflect an abnormal balance between motor activation and inhibitory control. The motor system may become excessively engaged during response execution, while inhibitory or evaluative control processes continue to operate strongly around the time of action. Such a mechanism could produce an electrophysiological signature of “over-controlled action”: responses are generated, but they are accompanied by heightened motor monitoring and increased demand for response regulation.

The social-hierarchy manipulation provides an additional mechanistic clue. The fact that LRP-r amplitudes were largest when OCD patients performed in a socially inferior position suggests that motor-control abnormalities are context sensitive rather than fixed. Social-evaluative threat is known to alter performance monitoring and cognitive control, particularly under conditions of low status, negative evaluation, or increased self-monitoring [153,154,155]. In OCD, such contexts may amplify the coupling between evaluative systems and motor-control systems. When the individual is placed in a socially threatening or subordinate position, increased concern about performance, mistakes, or external evaluation may heighten the need to regulate action output. The resulting state may intensify lateralised motor activation, producing larger LRP-r amplitudes. Conversely, the partial normalisation of LRP-r activity in the superiority condition suggests that reducing perceived evaluative threat may reduce the abnormal recruitment of motor-control mechanisms.

One possible neural interpretation is that fronto-cingulo-striato-motor circuits may be recruited more strongly during the transition from decision to response. Because these circuits were not directly measured, this account should be treated as a hypothesis rather than an established explanation of the LRP-r effect. The LRP is most closely related to activation over motor and premotor cortices, but response execution is shaped by upstream control systems, including medial frontal, prefrontal, and basal ganglia networks involved in action selection, inhibition, and performance monitoring [146,152]. In OCD, exaggerated monitoring signals may increase the influence of these control systems on motor output, especially in socially evaluative contexts. This could lead to stronger lateralised motor activation despite the absence of a simple motor-speed deficit. The abnormality may therefore be more consistent with altered cognitive–motor integration than with a simple motor-speed deficit.

Importantly, the LRP-r evidence should be interpreted cautiously. The included study did not directly correlate LRP-r amplitude with classical neuropsychological measures of executive function, inhibition, or processing speed. Therefore, the data do not prove that LRP-r abnormalities explain cognitive impairment in OCD. Instead, they provide indirect electrophysiological evidence that cognitive dysfunction may involve the late motor-control stage of action regulation. The most defensible interpretation is that OCD is associated with context-dependent abnormalities in the transformation of decisions into actions, particularly when social evaluation increases the need for control. Future studies should combine LRP-r with behavioural indices of response inhibition, reaction-time variability, post-error slowing, and executive performance to determine whether enlarged LRP-r represents compensatory motor recruitment, inefficient response execution, excessive action monitoring, or impaired suppression of competing motor tendencies.

### 4.9. Mechanisms of N450

The finding of reduced N450 activity in the autogenous OCD subgroup is compatible with less efficient recruitment of later conflict-monitoring or conflict-resolution processes. However, because this interpretation is based on a single subgroup study, it does not establish that altered N450 activity is the source of interference-control difficulties in autogenous OCD. In Stroop paradigms, the N450 is typically observed as a frontocentral or centroparietal negativity emerging approximately 350–500 ms after stimulus onset and is most often interpreted as an electrophysiological marker of conflict processing during the competition between a task-relevant stimulus dimension and an automatically activated but task-irrelevant dimension. Classic ERP studies of the Stroop effect showed that incongruent colour–word stimuli elicit a pronounced negativity in this time range, temporally linking the N450 to the stage at which the cognitive system detects or resolves interference between word meaning and colour naming [156,157]. This interpretation is consistent with broader cognitive-control models in which conflict signals, often associated with medial frontal/anterior cingulate activity, indicate the need to strengthen task-relevant processing and suppress competing response tendencies [112,158,159,160].

Against this background, the reduced, or less negative, N450 in autogenous OCD can be interpreted as evidence for weakened conflict-related neural differentiation during interference-control demands. In a typical Stroop task, efficient performance requires the participant to maintain the colour-naming goal, inhibit automatic semantic processing of the word, and resolve conflict when word meaning competes with the required response. If N450 amplitude reflects the strength or efficiency of this conflict-processing operation, then a reduced N450 suggests that autogenous OCD patients may generate a weaker neural signal when irrelevant semantic information must be controlled. Importantly, this abnormality was observed across negative, positive, and neutral words, which argues against a mechanism limited to negative emotional interference. Instead, the pattern suggests a more general disturbance in suppressing task-irrelevant stimulus meaning or in mapping detected conflict onto adaptive control.

This interpretation is strengthened by later work refining the functional meaning of the N450. Although early accounts often linked the component broadly to response conflict, numerical Stroop studies combining ERP with electromyography suggest that the N450 may be more closely related to stimulus-level or abstract representational conflict than to motor response conflict per se [161]. This distinction is mechanistically important for OCD. The emotional Stroop task used in the reviewed study did not simply require inhibition of an incorrect motor response; it required suppression of the meaning of emotionally valenced words while selecting the ink-colour response. A reduced N450 in autogenous OCD may therefore reflect impaired regulation of semantic or representational conflict before or partly independent of overt motor-response competition. In other words, the dysfunction may occur at the level at which intrusive, salient, or automatically activated meanings are evaluated as irrelevant to the current goal.

This mechanism is especially relevant to the distinction between autogenous and reactive obsessions. Autogenous obsessions are typically described as internally generated, intrusive, and ego-dystonic thoughts or images, whereas reactive obsessions are more often triggered by external cues. The reduced N450 in the autogenous group may indicate that these patients have difficulty down-regulating internally or automatically activated semantic representations once they enter the processing stream. During an emotional Stroop task, word meaning is irrelevant but difficult to ignore. If autogenous OCD is characterised by excessive spontaneous activation of intrusive representations combined with weak conflict-resolution signalling, then irrelevant word meanings may continue to compete with the task goal even when they are not specifically negative. This would explain why the N450 abnormality was generalised across word types rather than selectively tied to negative words.

The coexistence of reduced N450 with increased P2 and P3b amplitudes suggests a multi-stage disturbance rather than an isolated N450 deficit. Increased P2 may indicate enhanced early-to-middle stimulus classification, perceptual salience detection, or attentional capture by word stimuli. Increased P3b may reflect greater later allocation of attentional resources or context updating, consistent with the view that P3b amplitude increases when more cognitive resources are required for stimulus evaluation and task-relevant categorisation [70]. Mechanistically, this pattern may indicate that autogenous OCD patients allocate more resources to processing the stimulus but fail to translate this enhanced processing into efficient conflict resolution. Thus, the ERP profile may reflect a paradoxical combination of over-engagement at evaluative stages and under-efficiency at the specific stage where irrelevant information must be inhibited or resolved.

The behavioural slowing observed in the autogenous group fits this interpretation. Slower reaction times, especially during negative words but also more broadly across the task, may represent a compensatory strategy in the context of inefficient conflict processing. If the N450 signal is reduced, patients may not rapidly or efficiently resolve competition between word meaning and colour naming. As a result, they may rely on slower, more deliberate processing to maintain accuracy. This would produce generalised slowing without necessarily producing a large formal Stroop interference score. In this sense, the absence of a significant group difference in interference scores does not rule out cognitive dysfunction; rather, it may indicate that neural inefficiency is behaviourally expressed as prolonged processing time rather than as overt errors or condition-specific interference.

The N450 abnormality may therefore help explain why some cognitive deficits in OCD appear subtle or inconsistent at the behavioural level. ERP measures can reveal dysfunction in the timing and recruitment of control processes even when overt task performance is partially preserved. In the reviewed study, the autogenous group showed both slower behaviour and reduced N450 amplitude, but the absence of direct correlations between N450 and reaction time, accuracy, or interference scores prevents a strong claim that N450 activity directly determines cognitive performance. The most cautious interpretation is that reduced N450 reflects a neural mechanism consistent with poorer interference-control efficiency, while the behavioural consequences may depend on compensatory slowing, task difficulty, symptom subtype, and the extent to which the paradigm produces genuine conflict.

The broader literature also suggests that the N450 should not be interpreted in isolation. Stroop interference unfolds across multiple temporally distinct stages, including early perceptual attention, semantic activation, conflict detection, conflict resolution, and later response evaluation. Reviews of conflict-related ERPs emphasise that the N450 and later conflict slow potential may index related but separable aspects of conflict processing, with the N450 more closely tied to the emergence of conflict and the later positivity or slow potential reflecting sustained control, conflict resolution, or adaptation [162]. Therefore, reduced N450 in autogenous OCD may represent an early failure within a larger control cascade, potentially followed by compensatory late-stage processing reflected in increased P3b. This cascade model explains why the same patients may show increased amplitudes in some components and reduced amplitudes in others: OCD-related cognitive dysfunction may not be a simple global increase or decrease in cortical responsiveness, but a temporal misallocation of control resources.

### 4.10. Mechanisms of P50

The available P50 findings suggest that OCD may involve an abnormality at a very early stage of auditory information filtering, but they do not support a simple model in which impaired P50 gating directly produces measurable cognitive dysfunction. Mechanistically, the most important observation is that the abnormal paired-click response in study [28] was driven by an enlarged response to the second, repeated stimulus rather than by a reduced response to the first stimulus. This pattern indicates that the initial registration of auditory input was relatively preserved, whereas the inhibitory suppression of redundant sensory information was inefficient. In other words, the abnormality appears to reflect impaired gating rather than impaired sensory encoding. This distinction is important because P50 suppression is usually interpreted as a pre-attentive inhibitory mechanism that reduces the neural impact of repetitive or irrelevant input before it reaches later attentional and executive systems [97,163,164,165].

At the neural level, P50 generation and suppression are unlikely to depend on a single cortical source. Intracranial and source-localisation studies indicate that the temporal lobe, especially auditory cortical regions, is a major generator of the P50 response, whereas frontal regions contribute substantially to the suppression of the second response in paired-click paradigms [166]. Other electrophysiological and neuroimaging work has implicated a broader gating network involving superior temporal cortex, hippocampus, thalamus, and dorsolateral prefrontal cortex [167,168]. From this perspective, reduced P50 suppression in OCD may reflect a failure of fronto-temporal and limbic–thalamic circuits to rapidly classify repeated input as redundant. Such a mechanism would be compatible with an inefficient early ‘filter’ through which unnecessary sensory information may remain neurally salient, potentially increasing the processing demands placed on later attentional and control systems.

A second mechanistic level concerns inhibitory neurotransmission. Translational P50 research has repeatedly linked sensory gating to cholinergic modulation, particularly α7 nicotinic acetylcholine receptor mechanisms, and to local inhibitory interneuron function within hippocampal and related cortical circuits [164,169,170,171]. Although these models were developed primarily outside OCD, they provide a plausible biological framework for interpreting the OCD findings. If cholinergic–GABAergic modulation of early sensory responses is inefficient, the nervous system may fail to dampen repeated input within the first 50–100 ms of processing. In OCD, this could contribute to a state in which external stimuli, bodily sensations, or environmental details are insufficiently filtered at an early stage, thereby increasing the amount of information that must be evaluated by later cognitive-control mechanisms. However, the reviewed OCD data do not demonstrate that this mechanism directly explains executive dysfunction; rather, they suggest that early gating impairment may be one upstream contributor to a broader control burden.

This distinction is reinforced by the lack of significant correlations between P50 suppression and neuropsychological performance. Patients showed poorer processing speed, attentional flexibility, and executive attention, but P50 gating did not predict these deficits. Therefore, P50 dysfunction should not be interpreted as the direct electrophysiological substrate of reduced cognitive performance in OCD. A more cautious interpretation is that impaired P50 suppression represents a parallel early inhibitory abnormality that may increase vulnerability to attentional overload but requires additional dysfunction in later processing stages before it becomes behaviourally expressed. This interpretation is consistent with broader work showing that relationships between P50 gating and cognition are inconsistent and task-dependent. For example, studies in healthy participants have reported that P50 gating can relate to response inhibition or Stroop interference, but these associations differ across cognitive tasks and may depend on perceptual load, response strategy, and task modality rather than on a single general inhibitory mechanism [97,172].

Study [47] further supports this layered interpretation. In that study, P50 was examined within an active auditory discrimination task involving prepulse inhibition rather than in a passive paired-click paradigm. The reduced P50-PPI effect in OCD suggested that early sensory gating may again be attenuated, but this effect was weaker and less consistently robust than the abnormalities observed in schizophrenia. More importantly, P50-PPI was not the electrophysiological measure most closely linked to task performance. Instead, later target-processing measures, particularly target/non-target P3 differentiation, were more strongly associated with omission errors, perceptual sensitivity, and response criterion. This pattern suggests that early gating abnormalities may alter the quality or efficiency of incoming sensory processing, but behavioural performance depends more directly on later stimulus evaluation, target discrimination, and decision-related updating. Thus, P50 may represent a candidate correlate of altered early sensory processing, whereas later components such as P3 more directly reflect the cognitive operations that determine overt task performance.

The difference between passive P50 suppression and active P50-PPI also has mechanistic implications. Standard paired-click P50 suppression primarily reflects the inhibition of a repeated sensory response, whereas prepulse paradigms involve a broader interaction between early sensory registration, sensorimotor gating, attentional preparation, and response selection. Therefore, the weaker P50 findings in study [47] may reflect the fact that active discrimination tasks recruit multiple later processes that can compensate for, override, or obscure early sensory-gating abnormalities. In OCD, this may mean that early sensory filtering is inefficient, but its behavioural consequences vary depending on whether the task requires passive filtering, active discrimination, response inhibition, or strategic adjustment.

### 4.11. Mechanisms of P600

The available evidence on P600 in OCD is limited to a single working-memory study, and therefore mechanistic conclusions must remain preliminary. Nevertheless, the observed pattern is theoretically informative because it situates OCD-related cognitive dysfunction at a relatively late stage of information processing. In the included study, P600 was not elicited by the recall response itself, but during the interval after an auditory warning cue and before the digit sequence. Thus, the abnormality is best interpreted as a disturbance of anticipatory, rule-guided, late-stage preparatory processing rather than as a direct electrophysiological correlate of memory storage or retrieval. This distinction is important because the broader ERP literature shows that late positive components in the P300/P600/LPC family are sensitive not only to memory load, but also to context updating, task relevance, conscious evaluation, integration difficulty, and the allocation of attentional resources to task-relevant information [68,70,173,174].

A first mechanistic interpretation is that increased P600 amplitude in OCD reflects excessive late-stage allocation of cognitive resources during task preparation. In classical P300 theory, larger late positivities are associated with the updating of an internal task model when incoming information is relevant for future behaviour [68]. Polich’s integrative account similarly links P3b amplitude to attentional resource allocation and memory-updating operations, while latency is more closely related to the time required for stimulus evaluation [70]. Although the component in the included OCD study was labelled P600 rather than P3b, the timing, positivity, and cue-related working-memory context make this literature directly relevant. The warning cue informed participants whether digits would need to be recalled in forward or reverse order. Therefore, an increased P600 may indicate that OCD patients engaged an abnormally effortful preparatory updating process when translating the cue into a task set. Rather than efficiently configuring the upcoming working-memory operation, patients may have over-recruited late evaluative mechanisms to establish, check, or maintain the correct response rule.

This interpretation is strengthened by the fact that the amplitude increase was most evident over frontal, central, and right temporo-parietal sites. Working-memory preparation depends on coordinated frontoparietal and temporo-parietal activity: frontal systems support task-set maintenance, attentional control, and rule implementation, whereas parietal and temporo-parietal regions contribute to attentional orienting, stimulus–response mapping, and the updating of task-relevant representations. Late positive ERP activity is often maximal over centroparietal regions, but its functional interpretation depends on task context. In working-memory paradigms, similar late positive or slow-wave activity has been linked to storage, retention, and manipulation demands [175,176]. Thus, the right temporo-parietal P600 enhancement in OCD may reflect excessive recruitment of neural systems that prepare the individual to encode and manipulate the upcoming digit sequence. Mechanistically, this could represent compensatory activation: patients may require stronger late preparatory engagement to achieve a level of performance that still remains inferior to that of controls.

However, a purely compensatory interpretation is incomplete, because the P600 abnormality did not correlate significantly with memory performance. This absence of a direct electrophysiology–behaviour relationship suggests that increased P600 amplitude may not be the proximal mechanism causing poorer digit-span performance. Instead, it may reflect a parallel clinical-cognitive process that accompanies working-memory task performance in OCD. One possibility is excessive internal monitoring. The P600 has traditionally been studied in language, where it is elicited by syntactic violations, garden-path sentences, or integration difficulty [177,178,179,180]. Although these linguistic findings do not map directly onto a digit-span task, they show that P600 is not simply a “memory amplitude” but a late index of reanalysis, integration, and controlled revision of an internal representation. In OCD, an exaggerated P600 during the warning interval may therefore reflect excessive rechecking of the task rule or uncertainty about whether the correct preparatory state has been established. In this sense, the P600 abnormality may be more closely related to obsessional doubt and repetitive verification than to working-memory capacity itself.

The latency finding also supports a late-stage processing account. OCD patients showed prolonged P600 latency only at the right parietal P4 electrode, rather than a generalised delay across the scalp. In ERP theory, latency changes in late positive components are commonly interpreted as reflecting the duration of stimulus evaluation or categorisation processes rather than motor execution. Therefore, focal right parietal latency prolongation may indicate delayed completion of late evaluative operations within a right-lateralised attentional updating network. This mechanism could be clinically meaningful: even if patients can eventually identify the cue and prepare for the task, the process may be slower, more effortful, or less automatic. Such delayed updating would be consistent with a cognitive style characterised by prolonged uncertainty, inefficient closure of task preparation, and difficulty transitioning from evaluation to action.

The broader non-OCD clinical ERP literature provides useful parallels. In abstinent heroin users, abnormal P600 latency during a working-memory task was reported despite preserved memory performance, suggesting that P600 abnormalities can reveal altered late-stage information processing even when behaviour appears intact [181] (Papageorgiou et al., 2001). In multiple sclerosis, auditory P600 measures have been examined as correlates of working-memory dysfunction, and later work showed that treatment-related improvement in memory performance was accompanied by altered right frontal and right frontoparietal P600 activity [182,183]. These findings support two points relevant to OCD. First, P600 can be meaningfully elicited outside language paradigms, especially in tasks requiring rule-governed working-memory operations. Second, P600 abnormalities do not always map linearly onto behavioural impairment; they may index inefficient, compensatory, or clinically state-dependent processing.

A further mechanistic implication concerns cortico-striato-cingulo-parietal coordination. Some P600 accounts emphasise that late positive activity may depend not only on posterior cortical generators but also on cingulate and basal-ganglia systems involved in sequencing, rule-governed processing, and controlled updating. Frisch et al. (2003), for example, argued that basal-ganglia integrity contributes to modulation of the P600, distinguishing it from a simple P300 response [184]. This is relevant because the digit-span paradigm required the participant to transform an auditory cue into an ordered cognitive operation: maintain digits in the same sequence or manipulate them into reverse order. Such rule-governed sequencing is precisely the kind of process that may require interaction between frontal executive systems, cingulate monitoring mechanisms, basal-ganglia gating, and parietal updating networks. Increased P600 amplitude in OCD may therefore reflect inefficient gating of working-memory preparation: the system may detect the cue, activate the rule, and engage control networks, but it may fail to settle rapidly into a stable preparatory state.

The right-lateralised temporo-parietal emphasis is also notable. Right temporo-parietal and parietal regions are often implicated in attentional reorienting, salience detection, and the updating of internal models when task-relevant information changes. In the included study, the right temporo-parietal C4–T6/2 abnormality was the strongest group-discriminating feature, suggesting that P600 may be especially sensitive to altered right-hemisphere updating in OCD. Mechanistically, this may indicate that OCD patients over-assign salience to preparatory cues, even when the task is simple and repetitive. The warning tone may not merely trigger efficient task preparation; instead, it may produce excessive evaluative processing, reflecting an abnormal need to establish certainty before the memory sequence begins. This would explain why P600 was related more closely to obsessional complaints and obsessive traits than to digit-span accuracy.

### 4.12. Mechanisms of Pc

The limited Pc evidence suggests that later response-monitoring dysfunction in OCD may not be confined to exaggerated error detection but may also involve an impaired neural signal of correct action completion. In the broader ERP literature, response-locked performance monitoring is usually described as a sequence in which early medial-frontal activity detects conflict, error likelihood, or worse-than-expected outcomes, whereas later positive components support more conscious, evaluative, and metacognitive appraisal of the action that has just been executed [113,116,117,185,186,187]. Within this framework, the Pc can be interpreted as the correct-response counterpart of the Pe/Pc evaluative complex: it does not simply index the absence of an error but contributes to the internal registration that the performed response was appropriate. Therefore, the finding that OCD patients lacked a normal Pe/Pc differentiation is mechanistically important, because it suggests a disturbance in the neural coding of response correctness itself.

In healthy participants, Pe is typically larger after erroneous than correct responses and has been linked to later-stage error appraisal, awareness of mistakes, motivational significance, and confidence in response accuracy [187,188,189,190,191,192,193]. However, correct responses also elicit response-locked activity, including correct-response negativities and positive components, which appear to carry information about response certainty, subjective correctness, and post-decisional evaluation rather than merely motor execution [188,194,195,196,197,198]. This is relevant for OCD because compulsive checking may arise not only from exaggerated alarm signals after errors, but also from a weak or unreliable neural “all-clear” signal after correct actions. In other words, the problem may be less that every action is encoded as objectively wrong, and more that correct actions fail to generate sufficient post-response certainty.

The reviewed Pc finding fits this interpretation. In healthy controls, the Pe/Pc system differentiated incorrect from correct responses, indicating that later response-locked positivity carried discriminative information about whether the action outcome was erroneous or correct. In OCD patients, this differentiation was essentially absent over the medial region, despite preserved reaction time on correct trials. Mechanistically, this pattern suggests a failure of post-response evidence accumulation or metacognitive evaluation: after an action has been selected and executed, the monitoring system may fail to transform the available sensorimotor and task-context information into a stable internal representation of correctness. If replicated and shown to relate directly to confidence, such reduced differentiation could contribute to subjective uncertainty despite objectively correct performance. This interpretation is consistent with models proposing that later Pe-like activity reflects accumulated evidence for an error or confidence-related evaluation of the just-executed decision [192,196,197,198].

This mechanism also helps reconcile Pc findings with the broader ERP profile of OCD. Earlier components such as ERN are often interpreted in terms of rapid conflict detection, reinforcement-learning signals, or medial-frontal alarm mechanisms, whereas later positive components appear more closely related to conscious appraisal, confidence, and adaptive updating [113,116,117,187]. If early monitoring is hyperactive while later correctness confirmation is weak, the patient may experience a mismatch between “something may be wrong” and “the action is now complete and correct.” This imbalance may be relevant to theoretical models of repetitive checking, in which strong early alarm signals are accompanied by weak or unstable later confirmation of correct performance. However, checking behaviour and subjective certainty were not directly tested in the available Pc study.

The indirect evidence from the second study is consistent with this broader interpretation, although it concerned P3 rather than Pc. Early sensory-gating indices were not strongly related to discrimination performance, whereas later target-related P3 differentiation was associated with omission errors, perceptual sensitivity, and response criterion. This supports the idea that later positive ERP activity is more closely tied to higher-order evaluation and task-relevant decision processes than early automatic sensory filtering. In cognitive terms, Pc dysfunction may therefore belong to the same family of late evaluative abnormalities as altered Pe and P3: abnormalities that affect how the brain evaluates the meaning, certainty, and behavioural significance of actions or stimuli after initial processing has occurred.

### 4.13. Mechanisms of Pe

The Pe findings suggest that later-stage error processing in OCD cannot be reduced to a simple model of generalised hyperactive performance monitoring. Instead, they point to a more differentiated disturbance in the transformation of an early error signal into conscious, affective, and metacognitive evaluation of one’s own action. Classical ERP work distinguishes the ERN/Ne, which emerges rapidly after an erroneous response and is usually linked to early medial-frontal performance monitoring, from the later Pe, a centro-parietal positivity occurring several hundred milliseconds after the response and associated with conscious error appraisal, attentional orienting to the mistake, motivational significance, and confidence in response correctness [111,185,186,187,191]. Within this framework, the present findings suggest that OCD-related cognitive dysfunction may arise not only from excessive early error detection, but also from abnormalities in how the brain evaluates the meaning of an error after it has been detected.

One possible interpretation of the Pe findings concerns altered error awareness. Studies using anti-saccade and error-signalling paradigms have shown that Pe amplitude is typically larger for errors that participants consciously detect than for errors that remain unnoticed, whereas the relationship between ERN and awareness is less consistent [189,199,200]. This distinction is important for interpreting OCD. An enlarged Pe in some OCD or obsessive–compulsive-trait samples may be compatible with greater conscious or attentional processing of errors, although Pe amplitude alone cannot determine the subjective intensity of error experience. In such cases, the error may become a highly salient event that captures attention, triggers affective appraisal, and promotes cautious or perfectionistic control. This interpretation is consistent with findings in which enhanced Pe co-occurred with fewer errors or greater accuracy, but the cross-sectional associations do not determine whether enhanced Pe supported performance or resulted from a more cautious response strategy. Rather than indexing cognitive impairment in the narrow behavioural sense, a larger Pe may reflect an over-engaged evaluative system that supports accurate performance at the cost of excessive self-monitoring.

The socially modulated Pe findings can be interpreted in the same way. Broader performance-monitoring research indicates that the Pe is sensitive to the motivational significance of errors and resembles a P3-like response to salient, task-relevant events [187,191,201]. If an error occurs in a socially threatening or evaluative context, especially in a position of perceived inferiority, the mistake may carry greater self-relevant meaning. In OCD, where concerns about responsibility, failure, criticism, or harm are often clinically prominent, this may amplify later error appraisal. Mechanistically, the social context may increase the gain of the error-evaluation system: the same incorrect response produces a stronger conscious-affective response because the error is interpreted as more consequential. This would explain why Pe abnormalities may appear under socially threatening conditions but normalise when the participant occupies a more secure or superior position.

However, the reduced ΔPe findings suggest a second, partly opposite mechanism: impaired differentiation between erroneous and correct actions. Pe should be interpreted not only as an absolute positivity after errors, but also relative to the positivity following correct responses, often referred to as Pc. Studies of correct-response monitoring show that correct trials also elicit response-locked activity, and that Pc may reflect confidence, internal confirmation, or the subjective significance of correct performance [194,196,202,203]. Therefore, a reduced Pe–Pc difference does not necessarily mean that the brain fails to generate any late positivity after errors. Rather, it may mean that errors and correct responses are insufficiently separated at the level of later evaluative processing. This is mechanistically important for OCD because cognitive dysfunction may involve not only an exaggerated sense that something is wrong, but also a weakened internal signal that something has been done correctly.

This impaired error/correct discrimination provides a plausible electrophysiological mechanism for checking and doubt. In everyday action monitoring, adaptive behaviour requires two complementary signals: an alarm signal when an error occurs and a confirmation signal when the action has been completed correctly. If Pe/Pc differentiation is weak, the individual may retain an ambiguous internal representation of action outcome. The person may know that an action was performed, but the neural feedback confirming its correctness may be insufficiently distinct from the signal generated by error or uncertainty. In this model, repetitive checking is not simply a behavioural habit or a memory failure. It may reflect a metacognitive monitoring deficit in which internal feedback does not provide enough certainty that the intended action has been successfully completed.

The evidence-accumulation account of Pe further clarifies this mechanism. Steinhauser and Yeung proposed that error awareness emerges when internal evidence for an error accumulates until it crosses a decision threshold; in this model, Pe amplitude reflects the amount of accumulated evidence for an error rather than the mere presence or absence of an error-awareness response [192,204]. Applied to OCD, enlarged Pe may reflect excessive accumulation of evidence that an error has occurred, even when performance is objectively accurate or only mildly uncertain. Conversely, reduced ΔPe may indicate that the accumulated evidence does not sharply separate error from correctness. Both abnormalities can produce dysfunction, but through different routes: the first by making errors overly salient, the second by weakening confidence in action correctness.

This dual interpretation also explains why Pe findings are more heterogeneous than ERN findings. ERN abnormalities are often interpreted as reflecting early, relatively automatic performance-monitoring processes, whereas Pe is more dependent on awareness, attention, task instructions, motivational context, confidence, and developmental stage [116,187,200]. Therefore, Pe may be more state-sensitive and context-dependent than ERN. In paediatric or subclinical samples, enhanced Pe may accompany cautious, accurate responding and may reflect heightened evaluative vigilance. In clinical samples, especially when Pe is examined relative to Pc, the more relevant abnormality may be reduced outcome discriminability. Thus, the same disorder-related phenotype can involve either over-evaluation of errors or insufficient differentiation between error and correctness, depending on task context and analytic approach.

The developmental findings are particularly important. Developmental ERP studies show that Pe changes across childhood and adolescence, with later error positivity becoming more differentiated as performance monitoring matures [205,206]. Therefore, paediatric OCD findings should be interpreted against the background of ongoing maturation of error awareness and metacognitive control. A larger Pe in children with OC traits may reflect an early-emerging perfectionistic monitoring style that supports accuracy. By contrast, a reduced ΔPe in clinically affected youth may indicate that the maturation of later action-evaluation processes is altered, producing weaker discrimination between correct and erroneous responses during conflict tasks. This may explain why raw Pe amplitude and ΔPe can lead to different conclusions: raw Pe captures the strength of error evaluation, whereas ΔPe captures the precision with which the system separates error from non-error outcomes.

At the neural systems level, Pe abnormalities probably reflect distributed interactions between medial frontal monitoring systems, centro-parietal P3-like updating mechanisms, salience-network regions, and autonomic arousal systems. Error-awareness research has emphasised the role of the anterior insula and related salience-network activity in conscious error perception, autonomic orienting, and the recruitment of adaptive control after mistakes [200,207]. In OCD, this network-level mechanism may become dysregulated in two ways. First, errors may trigger excessive salience and arousal, producing enlarged Pe under threat, social evaluation, or perfectionistic performance demands. Second, the system may fail to generate a clear metacognitive distinction between error and correctness, producing reduced Pe/Pc separation and persistent uncertainty. These mechanisms are not mutually exclusive; they may coexist in different patients, developmental stages, or symptom contexts.

### 4.14. Plain EEG, Oscillatory Dysregulation, and Cognitive Dysfunction in OCD

The findings from plain EEG, quantitative EEG, source-localised EEG, and time–frequency analyses suggest that cognitive dysfunction in OCD is not explained by one isolated electrophysiological abnormality, but rather by disturbed coordination among several oscillatory systems that normally regulate cognitive control, attentional selection, performance monitoring, visuospatial organisation, memory, and behavioural flexibility. This interpretation is consistent with the broader view that neural oscillations are not passive by-products of cortical activity, but mechanisms through which distributed neuronal populations regulate input selection, temporal coordination, interregional communication, and adaptive behaviour [208,209,210,211]. Unlike ERP components, which capture discrete event-locked neural responses, plain EEG and time–frequency measures provide information about the ongoing and task-related oscillatory states within which cognition is implemented. Thus, the reviewed findings suggest that cognitive dysfunction in OCD may arise not only from abnormal responses to individual stimuli or errors, but also from abnormal background states that shape how the brain prepares for, monitors, inhibits, and adapts behaviour over time.

A central mechanism emerging from the reviewed studies concerns theta-band activity, particularly frontal and frontocentral theta during response monitoring and cognitive control. In non-clinical EEG research, mid-frontal theta has repeatedly been linked to conflict detection, error monitoring, action regulation, uncertainty, and the recruitment of control processes [120,212,213,214,215,216]. Cavanagh and Frank (2014) proposed that frontal theta may constitute a mechanism through which the need for cognitive control is represented and communicated across distributed brain networks [120]. Similarly, Cohen and Donner (2013) showed that conflict-related mid-frontal theta reflects behaviourally relevant oscillatory activity rather than merely an ERP-like transient response [216]. This framework helps explain why increased theta activity in OCD may have a dual meaning. On the one hand, enhanced theta may indicate excessive performance monitoring, heightened sensitivity to errors, or over-recruitment of control mechanisms. On the other hand, theta enhancement does not necessarily indicate cognitive failure, because frontal theta can increase when control is successfully and rapidly engaged [120,213,216].

This distinction is important for interpreting the paediatric time–frequency findings. When theta enhancement was error-specific, it likely reflected exaggerated performance monitoring after mistakes, consistent with the general role of theta in action monitoring and post-error control [212,213,217]. However, when theta elevation was present across both correct and incorrect responses, the mechanism may have been broader: a tonic or generalised state of over-monitoring, increased cognitive effort, or inefficient recruitment of medial-frontal control systems. In this sense, theta dysregulation may reflect excessive readiness to control behaviour rather than a direct marker of poor performance. This interpretation also explains why stronger theta activity may sometimes be associated with faster responding rather than slower responding. Theta may index rapid engagement of monitoring and control systems, but in OCD this engagement may be exaggerated, inefficiently sustained, or insufficiently differentiated between situations that truly require control and those that do not.

The reviewed beta-band findings suggest a complementary mechanism involving excessive inhibition, delayed behavioural re-engagement, and persistence of the current cognitive–motor state. In the broader EEG literature, beta oscillations have been associated with maintenance of the status quo, motor inhibition, top-down control, and reduced flexibility of action systems [218,219,220,221]. Engel and Fries (2010) proposed that beta activity is enhanced when the current sensorimotor or cognitive state is maintained, whereas pathological enhancement of beta may lead to abnormal persistence of the status quo and impaired flexible control [218]. This model is highly relevant to the reviewed findings in which greater post-error beta activity, or reduced beta suppression, was associated with slower error-trial and post-error reaction times. Mechanistically, reduced beta suppression after an error may indicate that the motor-control system remains in an inhibited or stabilised state for too long. Instead of rapidly detecting the error, updating control, and re-engaging behaviour, the system may remain excessively “held,” producing slowed post-error adjustment.

This beta mechanism is especially relevant to the cognitive phenotype of OCD because compulsive behaviour can be conceptualised as a failure to terminate monitoring and move flexibly into the next action. In healthy inhibitory-control studies, right frontal and motor beta activity has been linked to successful stopping and suppression of initiated responses [219,220,221]. However, in OCD, excessive or insufficiently suppressed beta after mistakes may reflect maladaptive persistence of inhibitory control. Such a mechanism could contribute to slowed executive performance, prolonged checking, delayed action completion, or excessive caution after perceived errors. Therefore, beta abnormalities may not simply indicate motor dysfunction; they may represent an oscillatory mechanism through which over-control becomes behaviourally costly.

The resting-state source-localised findings suggest another mechanism involving excessive low-frequency delta and theta activity in frontal, frontotemporal, cingulate, insular, and limbic-related regions. In awake cognition, delta activity is not merely a sleep-related rhythm; it has been linked to attention, motivational salience, interference suppression, and the regulation of large-scale brain states [222,223]. Harmony (2013) proposed that sustained delta oscillations may help inhibit networks that could interfere with task performance [223], whereas Knyazev (2012) emphasised the role of delta activity in motivational and salience-related processes [222]. However, excessive resting low-frequency activity, especially in frontal systems, may also indicate reduced cortical efficiency, altered arousal, impaired readiness, or abnormal inhibition of task-relevant networks. In the reviewed source-localised EEG findings, cognitively impaired OCD patients showed increased delta and theta current density, whereas cognitively unimpaired patients did not differ clearly from controls. This pattern suggests that low-frequency abnormalities were more closely related to cognitive dysfunction than to OCD diagnosis alone.

Mechanistically, excessive frontal and fronto-limbic delta/theta activity at rest may reflect a brain state in which executive systems are not optimally prepared for rapid attentional shifting, visual scanning, psychomotor speed, and flexible set switching. This could explain the association with impaired Trail-Making Test performance, which requires coordinated visual search, motor speed, sequencing, attentional shifting, and executive control. From a network perspective, excessive resting low-frequency activity may reduce the efficiency with which frontal control systems transition from an internally monitored state to an externally oriented task state. This interpretation is compatible with broader models in which frequency-specific oscillatory activity acts as a “spectral fingerprint” of large-scale neuronal coordination [211]. In OCD, the relevant abnormality may therefore be not simply “more delta” or “more theta,” but an altered resting oscillatory configuration that makes executive networks slower, less flexible, and more vulnerable to interference.

Alpha-band abnormalities provide another major mechanistic pathway. Traditional interpretations treated alpha as an idling rhythm, but contemporary EEG research strongly supports the view that alpha oscillations regulate functional inhibition, sensory gating, attentional selection, and access to stored information [224,225,226]. According to the “gating by inhibition” model, alpha synchronisation helps suppress task-irrelevant regions or representations, thereby shaping the functional architecture of the working brain [224]. Similarly, Klimesch (2012) argued that alpha supports both inhibition and timing, allowing controlled access to task-relevant information [226]. Foxe and Snyder (2011) also emphasised alpha as a mechanism of sensory suppression during selective attention [225]. These models help explain why alpha abnormalities in OCD may contribute to cognitive dysfunction through more than one route.

First, reduced resting slow-alpha power may reflect excessive cortical activation or insufficient inhibitory stabilisation of executive networks. In the reviewed qEEG findings, reduced alpha was associated with slower performance on executive tasks. This does not necessarily mean that patients were under-engaged. Rather, reduced alpha may indicate that cortical systems are excessively activated, insufficiently filtered, or unable to maintain efficient task-related inhibition. This interpretation fits the idea of executive hypercontrol: patients may perform cognitive tasks slowly because they over-recruit attention, monitoring, response selection, and self-regulatory control. In broader memory and cognition research, alpha and beta desynchronisation can support information processing, but excessive or poorly regulated desynchronisation may also increase neural noise or cognitive effort [226,227]. Thus, reduced alpha in OCD may reflect a costly mode of cognitive engagement in which performance is maintained through excessive controlled processing rather than efficient automatic organisation.

Second, reduced or poorly organised prestimulus alpha may indicate impaired anticipatory inhibition before selective-attention demands. Studies of visuospatial attention have shown that alpha activity is modulated before stimulus onset and can bias perception toward task-relevant locations while suppressing irrelevant information [228,229,230,231,232]. Worden et al. (2000) showed that anticipatory alpha changes index spatial attentional bias [228], while Kelly et al. (2006) argued that increased alpha reflects active suppression of distracting information rather than passive idling [231]. Thut et al. (2006) further showed that occipital alpha activity predicts visual target detection [230]. Therefore, reduced prestimulus alpha in OCD may indicate a failure to suppress irrelevant sensory or semantic information before it competes for processing. Under this mechanism, patients may enter selective-attention tasks with insufficient top-down gating, making them more vulnerable to distraction, perseveration, and inefficient set shifting.

This alpha-gating mechanism also helps explain the relationship between task-related alpha/theta activity and Wisconsin Card Sorting Test performance. Cognitive flexibility requires not only detecting the relevant rule but also suppressing previously relevant rules and disengaging from perseverative response tendencies. If occipital or posterior alpha/theta responses are abnormal during selective attention, irrelevant stimulus features may continue to influence performance, resulting in perseverative responses and fewer achieved categories. This interpretation is consistent with the broader finding that alpha oscillations support distractor suppression and attentional selection across sensory systems [224,225,226,233]. Therefore, in OCD, abnormal alpha dynamics may contribute to cognitive inflexibility by weakening the oscillatory mechanism that normally protects task goals from interference.

The reviewed qEEG asymmetry findings add a further mechanism involving abnormal hemispheric balance, particularly in visuospatial and nonverbal memory. In EEG studies of spatial attention, alpha lateralisation is a robust marker of attentional allocation and suppression of irrelevant visual space [228,229,230,232]. More broadly, visuospatial construction and nonverbal memory depend strongly on right-hemisphere and frontoparietal systems involved in spatial integration, global organisation, and attentional orienting. The reviewed findings linking reduced right-sided beta2 activity or altered frontal alpha asymmetry with poorer visuospatial memory and Rey–Osterrieth Complex Figure performance suggest that some cognitive deficits in OCD may reflect a lateralised imbalance rather than a generalised memory impairment. Specifically, insufficient right-hemisphere activation may impair holistic spatial organisation, whereas excessive left-frontal activation may promote overly analytic, sequential, verbally mediated, or detail-focused strategies.

This mechanism is particularly useful for explaining why verbal memory may be relatively preserved while visuospatial or nonverbal memory is impaired. If patients rely excessively on left-frontal control strategies, they may approach complex visual material in a fragmented and over-controlled way, focusing on local details rather than global configuration. By contrast, greater right-frontal activation may support more efficient spatial planning, gestalt organisation, and constructional strategy. Thus, EEG asymmetry findings suggest that cognitive dysfunction in OCD may involve not only excessive monitoring, but also an imbalance in the type of control being applied. The problem may be too much analytic, rule-based, left-frontal control and too little flexible, integrative, right-hemisphere processing during visuospatial tasks.

Taken together, the plain EEG findings support a mechanistic model in which cognitive dysfunction in OCD reflects abnormal oscillatory regulation across frontal, fronto-limbic, posterior attentional, and lateralised hemispheric networks. Frontal theta abnormalities suggest heightened monitoring and altered recruitment of cognitive control systems [120,212,213]. Beta abnormalities may be related to stronger or more sustained inhibitory processing, delayed release from post-error control states, or persistence of the current cognitive–motor set [218,219,221]. Resting delta/theta source abnormalities may identify patients with reduced frontal network efficiency and slower executive processing. Alpha abnormalities suggest impaired inhibitory gating, excessive cortical activation, and deficient suppression of irrelevant information. Hemispheric asymmetries were associated with visuospatial and nonverbal performance and may be compatible with relatively reduced right-hemisphere engagement or greater reliance on left-frontal analytic strategies. The available studies do not establish that these asymmetries caused the cognitive deficits.

This model also clarifies why the reviewed EEG–cognition relationships were heterogeneous. Oscillatory abnormalities may be compensatory in some contexts and maladaptive in others. Increased theta may support rapid control recruitment, but if generalised or excessive, it may reflect inefficient over-monitoring. Reduced alpha may indicate increased engagement, but if poorly timed or spatially disorganised, it may indicate a failure of inhibitory gating. Increased beta may support stopping, but if sustained after errors, it may slow adaptive re-engagement. Increased low-frequency activity may help suppress interference, but if excessive at rest, it may reflect inefficient frontal network readiness. Therefore, plain EEG abnormalities in OCD should not be interpreted as simple biomarkers of global cognitive impairment. They may be considered candidate frequency- and network-specific correlates of altered cognitive regulation, pending replication in larger and methodologically harmonised studies.

The strongest mechanistic implication is that cognitive dysfunction in OCD may arise from an imbalance between monitoring, inhibition, and flexibility. The observed pattern is compatible with stronger control-related signalling, more sustained inhibitory activity, and less efficient sensory or executive gating. However, these functional interpretations are inferred from the known roles of the frequency bands and were not directly established by the included studies. This combination could be associated with a cognitive profile in which simple task accuracy is sometimes preserved, or even accompanied by fast responding, but more complex executive functions are slowed or inflexible. In practical terms, this may manifest as slower executive speed, impaired set shifting, increased perseveration, poorer visuospatial organisation, and selective nonverbal memory deficits. Plain EEG therefore provides complementary evidence suggesting that cognitive dysfunction in OCD may be related not only to event-related responses but also to broader oscillatory states associated with control, attention, inhibition, and memory.

The resting-state and quantitative EEG findings require particularly cautious interpretation because all five studies in this part of the synthesis [58,59,60,61,62] were judged to have a serious overall risk of bias. The principal concerns were small convenience samples, incomplete adjustment for medication and comorbidity, participant selection, and the examination of multiple frequency bands, scalp regions, neuropsychological outcomes, and correlations. The convergence of these studies on altered alpha, theta, beta, and delta activity is mechanistically informative, especially where EEG measures were directly associated with executive or visuospatial performance. Nevertheless, these findings should presently be regarded as hypothesis-generating rather than as robust evidence for reproducible resting-state biomarkers of cognitive dysfunction in OCD.

### 4.15. Methodological and Clinical Heterogeneity as an Explanation for Inconsistent Findings

The variability of the reviewed findings should not be interpreted solely as an absence of reproducible electrophysiological abnormalities in OCD. In many cases, apparently inconsistent results may reflect differences in the clinical populations, cognitive operations, and EEG measures used across studies. This distinction is important because two studies may assign the same ERP label to responses elicited under substantially different cognitive demands or may examine clinically different forms of OCD using analytically non-equivalent EEG procedures.

Medication exposure is one plausible source of variation. Some studies recruited drug-naïve or medication-free participants, whereas others included patients receiving stable serotonin reuptake inhibitors, additional psychotropic medication, or previous treatment followed by washout. Medication may influence cortical arousal, processing speed, attentional allocation, inhibitory control, error monitoring, and spectral power. It may also reduce symptoms without fully normalising cognitive control, producing electrophysiological patterns that differ from those observed in untreated patients. Conversely, medicated samples may include individuals with greater illness severity, chronicity, or treatment resistance. A difference between medicated and unmedicated studies may therefore reflect pharmacological effects, clinical selection, or both. Because medication was rarely randomised and was not consistently modelled statistically, its precise contribution cannot be separated from illness-related effects.

Symptom heterogeneity may also explain why the same component was not altered consistently. The reviewed studies included autogenous and reactive OCD, washing and checking presentations, tic-related and non-tic-related OCD, current and lifetime diagnoses, treatment-resistant patients, unaffected relatives, and individuals with elevated subclinical symptoms. These groups are unlikely to engage monitoring, threat processing, inhibition, and feedback learning in identical ways. For example, personally threatening stimuli may preferentially amplify early attentional capture in symptom-relevant groups, while checking-related presentations may show stronger repetition facilitation or incomplete certainty signalling. Tic-related OCD may additionally differ from non-tic-related OCD in the contribution of motor-control and performance-monitoring systems. Analyses that collapse across symptom dimensions may therefore attenuate effects that are present only in clinically specific subgroups.

Developmental differences are particularly relevant to the ERN literature. Paediatric and adolescent samples often showed enlarged ERN and, in several studies, associations with better accuracy or fewer errors. Adult findings were more heterogeneous and often lacked direct relationships with behavioural performance. Several explanations are possible. Error-monitoring systems continue to mature across childhood and adolescence, and compensatory monitoring may be more detectable earlier in the disorder. Adult samples may have longer illness duration, greater treatment exposure, more comorbidity, or different strategies for controlling errors. Studies of lifetime paediatric OCD and unaffected relatives may additionally capture trait-like liability, whereas adult clinical studies may reflect a mixture of vulnerability, chronic adaptation, and current symptom state. Age should therefore be considered a potential moderator rather than treated only as a demographic characteristic.

Cognitive paradigms further complicate direct comparison. Go/NoGo, stop-signal, flanker, antisaccade, oddball, emotional Stroop, reversal-learning, and gambling tasks impose different demands even when they are described under a common domain such as “inhibition” or “monitoring.” Reduced N2 during response withholding and enlarged Stop-N2 during cancellation of an initiated action are not necessarily contradictory because they may reflect different mixtures of conflict detection, inhibition, attention, and task difficulty. Likewise, enhanced ERN in a flanker task may coexist with reduced learning-related ERN or FRN during probabilistic learning if internal response monitoring is heightened but external feedback utilisation is inefficient. P3 direction may also vary according to whether the component indexes neutral target detection, inhibitory evaluation, emotional salience, or feedback updating. Paradigm-specific interpretation is therefore preferable to assuming that each ERP component has a single fixed cognitive meaning.

Acquisition and processing differences may have contributed additional variability. The included studies used different electrode montages, scalp references, filter settings, sampling parameters, artefact-correction procedures, baseline intervals, latency windows, and amplitude definitions. Some quantified peak amplitude, others mean amplitude or difference waves, and some used source localisation or time–frequency measures rather than conventional scalp ERPs. These approaches differ in sensitivity to latency variability, broad component morphology, overlapping activity, and noise. In OCD, where delayed or prolonged processing may be relevant, fixed latency windows may underestimate an abnormal response if its timing differs between groups. Electrode selection and multiple testing across scalp sites may also produce apparently inconsistent topographies, particularly in small samples.

The number and quality of accepted trials represent another important concern. Reliable ERN estimates depend on sufficient error trials, while FRN and feedback-related components depend on adequate numbers of relevant outcome events. Participants with very few errors, poor task engagement, excessive movement, or low-quality EEG were often excluded. Although necessary for signal reliability, these procedures may select cognitively atypical subsets of the original samples. Differences in minimum-trial thresholds and exclusion rules may consequently change both ERP reliability and the clinical characteristics of the analysed participants.

These sources of heterogeneity suggest that the reviewed literature should not be reduced to a simple count of positive and negative studies. Greater weight should be given to findings that replicate across conceptually similar paradigms, remain present after accounting for medication and clinical covariates, use reliable component estimates, and demonstrate direct electrophysiological–behavioural relationships. Conversely, findings based on small subgroups, exploratory electrode-level analyses, few accepted trials, or poorly matched clinical samples should be treated as preliminary. Future studies should report medication dose and duration, symptom dimensions, illness stage, age of onset, and comorbidity systematically; use harmonised acquisition and preprocessing protocols; and replicate effects within the same cognitive paradigm before attempting broad cross-task generalisation.

## 5. Conclusions

This systematic and mechanistic review shows that cognitive dysfunction in obsessive–compulsive disorder is associated with abnormalities across several electrophysiological stages of information processing. The reviewed EEG, ERP, qEEG, and time–frequency studies do not point to one single marker of cognitive impairment in OCD. Instead, they suggest a broader pattern of dysregulation involving sensory gating, attentional allocation, inhibitory control, conflict monitoring, error processing, feedback learning, response preparation, working-memory preparation, and oscillatory control of cognitive networks.

The most consistent evidence concerns performance monitoring and cognitive control. ERN findings indicate that OCD is frequently associated with enhanced early error detection or excessive internal monitoring, although this is not always accompanied by poorer behavioural performance. Pe and Pc findings suggest that later error evaluation and the subjective appraisal of correct versus incorrect actions may also be altered, potentially contributing to persistent doubt and difficulty achieving a sense of task completion. N2/N200 and P3/P300 findings further indicate abnormalities in inhibitory control, stimulus evaluation, attentional resource allocation, and context updating, but these effects are strongly task-dependent and vary across paradigms.

Earlier components such as P50, N1/N100, P2/P200, and N450 suggest that OCD may also involve abnormalities in sensory filtering, early attentional selection, stimulus evaluation, and interference control, particularly when stimuli are repetitive, emotionally salient, or personally threatening. FRN findings point to altered feedback processing and reinforcement learning, whereas limited P600 evidence suggests that working-memory dysfunction may involve inefficient anticipatory preparation. Plain EEG and time–frequency studies add that OCD-related cognitive dysfunction may be linked to abnormal oscillatory activity, especially in theta, alpha, beta, and delta bands, reflecting altered monitoring, inhibition, arousal, and network efficiency.

Overall, the findings support a model in which cognitive dysfunction in OCD results from an imbalance between excessive monitoring, inefficient inhibition, and reduced flexibility. Patients may detect errors, conflict, threat, or uncertainty too strongly, while also struggling to disengage from previously activated thoughts, responses, or task sets. This may explain why behavioural performance is sometimes preserved despite abnormal electrophysiological activity: patients may compensate through increased effort, over-monitoring, or inefficient control strategies. However, such compensation may become costly in demanding situations, contributing to slowed responding, perseveration, impaired cognitive flexibility, abnormal feedback learning, and persistent doubt.

The current evidence should be interpreted cautiously because studies differed in sample size, medication status, comorbidity, age group, symptom profile, EEG methodology, task design, and statistical approach. Many studies examined EEG or ERP activity during cognitive tasks without directly testing correlations with independent cognitive-performance measures. Therefore, these electrophysiological markers should currently be viewed as mechanistic indicators rather than validated clinical biomarkers.

Future studies should use larger and better-characterised samples, standardised EEG/ERP paradigms, direct brain–behaviour analyses, and longitudinal designs. Combining ERP, oscillatory, source-localisation, neuropsychological, symptom-dimensional, and computational approaches may help clarify how abnormal monitoring, inhibition, uncertainty processing, and cognitive flexibility interact in OCD.

In conclusion, EEG-based evidence suggests that cognitive dysfunction in OCD is not simply a general reduction in cognitive ability. Rather, it reflects a dysregulated control system characterised by excessive monitoring, abnormal attentional gating, inefficient inhibition, altered feedback processing, and reduced flexibility. Electrophysiological methods are therefore valuable for understanding how OCD-related cognitive abnormalities unfold over time and may help inform more precise cognitive, behavioural, and neuromodulatory interventions.

## Figures and Tables

**Table 1:** Characteristics and principal findings of the included ERP studies, grouped by primary ERP component.

Interpretation for the Review	ERP–Cognitive-Function Relationship	Cognitive Domain/Task	ERP Component(s)	Participants	Study
A. Early sensory and attentional components: P50, P1, N1/N100, and P2/P200					
Early sensory-gating abnormalities appear separable from executive/processing-speed deficits.	OCD showed impaired P50 suppression and poorer SDMT/TMT performance, but P50 T/C ratio did not correlate with any cognitive test score.	Paired-click sensory gating; memory, digit span, SDMT, TMT	P50	26 OCD patients; 26 healthy controls	Study [28]
Early inhibitory gating indexed by P1 may support reactivation of repetitive/recently used task sets in OCD, suggesting a more nuanced profile than simple “cognitive inflexibility.”	OCD showed smaller backward-inhibition RT cost and larger target-P1 modulation, but no direct ERP–RT correlation was reported. P1 correlated with OCD symptom dimensions.	Backward-inhibition task-switching; cognitive flexibility	P1, N1, N2, P3	20 adolescents with OCD; 22 healthy controls	Study [24]
Supports a possible excessive-facilitation mechanism in OCD, especially checking/non-washing symptoms, rather than only failed inhibition.	Repetition produced RT facilitation across groups. OCD non-washers/checkers showed exaggerated RT facilitation. OCD showed abnormal N1 repetition effects and reduced P2, especially in non-washers/checkers; no formal ERP–RT correlation was reported.	Go/NoGo repetition-priming paradigm	P1; N1; P2; N2; P3	20 OCD patients; 20 panic disorder patients; 20 healthy controls; OCD split into washers and non-washers/checkers	Study [54]
Emotional Stroop interference may arise from both early attentional capture by threat and later cognitive elaboration.	Participants with emotional Stroop interference showed larger P1 and P3 responses to threat words. OCD showed early P1 amplification and longer N1 latency to personally threatening words.	Emotional Stroop and word-classification tasks using personally threatening words	P1; N1; P3	20 OCD patients; 20 panic disorder patients; 20 healthy controls	Study [55]
B. Conflict and response-inhibition components: N2/N200, N450, and inhibitory-control P3					
ERP abnormalities may reveal latent inhibitory-control dysfunction not expressed at the behavioural level.	OCD showed reduced frontocentral N2 and prolonged NoGo-P3 latency, but N2/P3 amplitudes did not correlate with omission or commission errors.	Visual Go/NoGo; response inhibition	N2, P3	15 OCD patients; 15 healthy controls	Study [22]
Stop-N2 abnormality accompanies impaired response inhibition at the group level, but cannot be treated as an individual-level predictor of inhibitory performance.	OCD showed longer SSRT and larger frontal Stop-N2, but no direct correlation between Stop-N2 and SSRT was reported.	Stop-signal task; motor response inhibition	Stop-N2, Stop-P3	42 medication-free OCD patients; 42 healthy controls	Study [23]
Autogenous OCD may involve stronger interference-control dysfunction, whereas both subtypes show later inhibitory-control inefficiency. Direct ERP–performance correlations were largely negative.	Autogenous OCD showed Stroop slowing and P2/P3b/N450 abnormalities. Both OCD subtypes showed longer SSRT and reduced Stop-P3, but Stop-P3 latency did not correlate with SSRT.	Emotional Stroop; stop-signal task	P1; N1; P2; P3b; N450; Stop-N2; Stop-P3; Go-N2	Behavioural sample: 97 medication-free OCD patients and 62 controls; ERP subsample: 25 autogenous OCD, 25 reactive OCD, 31 controls	Study [53]
C. P3/P300: attention, stimulus evaluation, and context updating					
P300 amplitude may index the efficiency of selective attention/inhibitory control in OCD even when overt neuropsychological impairment is not detected.	OCD showed shorter P300 duration. Within OCD, longer Stroop interference duration correlated with lower P300 amplitudes in occipital, parietal, and anterior temporal regions.	Auditory oddball; Stroop, TMT, Design Fluency, verbal fluency	P300	31 OCD patients; 30 healthy controls	Study [21]
Frontal P300 reduction may be related to controlled attention, set-shifting, and executive flexibility in OCD, but the evidence should be presented cautiously.	In OCD, smaller frontal P300 amplitudes were associated with slower TMT-B performance, although these were exploratory and did not survive strict Bonferroni correction.	Auditory oddball; broad neuropsychological battery	P300, N100	19 OCD patients; 22 schizophrenia patients; 21 healthy controls	Study [27]
Paediatric P300 attenuation may indicate altered attentional processing without direct coupling to response-time variability.	OCD showed reduced P300 amplitude at Fz, C3, and C4, but P300 measures did not correlate with ex-Gaussian RT parameters.	Auditory oddball; target detection and RT variability	P300	15 drug-naïve paediatric/adolescent OCD patients; 15 healthy controls	Study [29]
Reduced P300, increased P200, and executive/visuospatial impairment may represent candidate endophenotypic markers of OCD vulnerability.	Smaller P300 amplitude correlated with longer TMT-B completion time in both OCD patients and siblings.	Auditory oddball; broad neuropsychological assessment	N100, P200, N200, P300	33 OCD patients; 18 unaffected siblings; 21 healthy controls	Study [31]
Later target-processing ERPs, especially P3, appear more cognitively meaningful than early sensory-gating indices.	Early P50-PPI, EMG-PPI, and N1-PPI did not predict discrimination performance. Later target-related P3 differentiation was related to omissions, sensitivity, and response criterion.	Auditory prepulse inhibition during target discrimination	P50; N1; P2; N2; P3; CNV	11 OCD patients; 9 schizophrenia patients; 21 healthy controls	Study [47]
D. P600: working-memory preparation					
P600 may reflect altered preparatory/late-stage processing in OCD, but not directly the severity of working-memory impairment.	OCD showed poorer digit recall, larger P600 amplitudes, and prolonged P600 latency at P4, but P600 abnormalities did not correlate with memory performance.	Digit-span working-memory task	P600	20 drug-free OCD patients; 20 healthy controls	Study [26]
E. ERN and CRN: early response and error monitoring					
Error-monitoring ERPs and affective evaluation of errors may be dissociable in OCD.	ERN did not predict negative affective priming after errors, either trial-by-trial or across participants. CRN also did not explain valence-specific processing.	Go/NoGo plus affective word categorization; affective evaluation of errors	ERN, CRN	28 OCD patients; 28 healthy controls	Study [25]
Enlarged ERN may function as a trait-like/error-monitoring biomarker rather than a marker of overt task impairment.	OCD patients and siblings showed enlarged ERN, but ERN did not correlate with number of errors, RTs, or post-error slowing.	Flanker task; error monitoring	ERN, CRN	40 paediatric OCD patients; 19 unaffected siblings; 40 healthy controls	Study [33]
ERN may relate to behavioural adjustment around errors, but this relationship is not specific to OCD because anxiety groups also showed enlargement.	Larger ERN was associated with incorrect-trial RT and post-error slowing, but not with number of errors or correct-trial RT.	Flanker task; response conflict/error monitoring	ERN, CRN	26 youths with OCD; 13 youths with non-OCD anxiety; 27 healthy controls	Study [34]
OCD-related performance monitoring is context-dependent rather than uniformly hyperactive.	No classical ERP–performance correlation was reported. ERN varied with learning context and error-likelihood: OCD showed exaggerated monitoring in lower-conflict errorless recognition but reduced differentiation in higher-conflict errorful recognition.	Errorless/errorful learning followed by recognition memory	ERN	16 OCD patients; 16 healthy controls	Study [36]
Enlarged ERN in non-tic-related paediatric OCD may partly reflect compensatory monitoring related to error frequency.	In OCD, especially non-tic-related OCD, more errors were associated with smaller/less negative ERN. ERN did not correlate with RTs or post-error slowing.	Flanker task; error monitoring	ERN, CRN	44 youths with OCD, including 9 tic-related and 35 non-tic-related; 44 healthy controls	Study [37]
ERN may index accuracy-related monitoring efficiency in paediatric OCD rather than general slowing or post-error behavioural adjustment.	In OCD, larger ERN was associated with better accuracy. ERN did not correlate with RT or post-error slowing; CRN/dERN related more to response speed.	Flanker task; accuracy and response speed	ERN, CRN, dERN	80 youths with OCD; 80 healthy controls	Study [38]
ERN and CRN capture different performance dimensions: ERN relates to accuracy/error monitoring, whereas CRN relates more to response speed.	Across participants, better accuracy was associated with larger ERN. CRN correlated with RTs, not accuracy.	Flanker task; error monitoring	ERN, CRN	53 adolescents with OCD; 36 with MDD; 89 healthy controls	Study [39]
ERN may reflect general motivational/error-monitoring significance rather than OCD-specific cognitive dysfunction.	Across all participants, more commission errors were associated with smaller ERN, but this relationship was not OCD-specific.	Flanker task; ERP–MRI study of error monitoring	ERN, dERN	20 paediatric OCD patients; 20 healthy controls	Study [40]
Enlarged ERN may be a familial/endophenotypic marker of OCD vulnerability rather than a direct marker of task performance.	OCD patients and relatives showed enlarged ERN, but ERN did not correlate with error rate, reaction time, or post-error slowing in any group.	Flanker task; error monitoring and post-error adjustment	ERN; CRN	30 OCD patients; 30 unaffected first-degree relatives; 30 healthy controls	Study [42]
Social inferiority may amplify error-monitoring, cognitive-control, and motor-control abnormalities in OCD; ERP effects are clinically meaningful but not direct ERP–performance correlations.	The study mainly correlated ERP measures with clinical severity rather than cognitive performance. Y-BOCS correlated with ERN social modulation and N2 amplitude in the socially inferior condition.	Social hierarchy visual discrimination task; error monitoring, cognitive control, motor control	ERN; Pe; N2; LRP-r	20 OCD outpatients; 20 healthy controls	Study [43]
OCD-related ERN enhancement may reflect altered midfrontal theta power/response monitoring more than a specific planning-performance deficit.	EEG–behaviour correlations with broader clinical/cognitive measures were small overall. Task accuracy was associated with larger ITPC and ERP amplitudes, but not clearly with theta total power.	Flanker task; also Tower of London planning	ERN; CRN; ΔERN; theta total power; theta ITPC	99 youth with OCD; 99 healthy controls	Study [46]
Enhanced ERN may be transdiagnostic and not necessarily tied to overt behavioural performance.	Health-anxiety and OCD groups showed enlarged ERN, but ERN/CRN did not correlate significantly with error counts or RTs within groups.	Modified flanker task	ERN; CRN	24 high-health-anxiety participants; 24 OCD patients; 24 healthy controls	Study [51]
F. ERN with Pe and/or Pc: early and late response evaluation					
OCD may involve relatively intact early ERP error detection but abnormal post-error neural adjustment/default-network regulation.	ERN amplitude did not correlate with error rate; meaningful performance-related findings were stronger for fMRI dACC activation than ERP.	Antisaccade/error-processing task; post-error adjustment	ERN, Pe	21 OCD patients; 20 healthy controls	Study [32]
Error-monitoring enhancement in OC traits may partly reflect a cautious, vigilant, or perfectionistic response style.	Higher OC-like behaviours were associated with fewer errors, larger ERN, and larger Pe. Number of errors also correlated with ERN and Pe; after controlling for errors, ERP effects explained less unique variance.	Flanker task; error monitoring	ERN/Ne; Pe	37 nonclinical 10-year-old children	Study [44]
Paediatric OCD shows hyperactive error monitoring, but theta/ERN and beta may reflect different mechanisms: rapid monitoring versus behavioural inhibition/slowing.	OCD showed larger ERN and greater error-related theta/beta abnormalities. Error-related beta was associated with slower error/post-error RTs, whereas theta and ERN were associated with faster task performance.	Flanker task; response conflict and post-error adjustment	ERN; Pe/Pc; error-related theta and beta activity	27 early adolescents with OCD; 27 healthy controls	Study [45]
Strong evidence that both early error detection and later error evaluation relate to cognitive performance in paediatric OCD.	Higher accuracy correlated with larger/more negative ERN and with larger Pe/ΔPe. Regression showed ERN and ΔPe independently predicted task accuracy.	Flanker task; accuracy and error monitoring	ERN; CRN; Pe; Pc; ΔERN; ΔPe	105 youth with lifetime OCD; 105 healthy controls	Study [50]
ERP markers may index clinical phenotype and treatment resistance more than task-level cognitive performance.	OCD patients made more errors, but the study did not report clear direct ERP–performance correlations. ERN correlated with Y-BOCS severity, and CRN correlated with treatment resistance.	Visual flanker task	ERN; CRN; Pe; Pc	26 primary OCD patients; 26 matched healthy controls	Study [52]
G. FRN and RewP: feedback evaluation and reinforcement learning					
OCD may involve impaired external feedback monitoring during learning; FRN appears relatively diagnosis-related, whereas feedback-P300 may be more performance-related.	OCD showed more reversal/exploration errors and reduced FRN to exploration negative feedback. FRN effects remained after controlling for errors, whereas P300 effects were related to behavioural errors.	Four-choice reversal learning; feedback-guided behavioural adaptation	FRN, feedback-related P300	25 OCD patients; 25 healthy controls	Study [30]
OCD may involve impaired reward learning or reduced trust in positive feedback rather than simply exaggerated punishment monitoring.	OCD showed poorer feedback learning and more shifting after wins. FRN/RewP abnormalities were strongest for win feedback during active learning. Model-based analyses showed that FRN tracked prediction errors and ERN increased when subjective predictive accuracy was lower.	Active and observational probabilistic feedback learning	FRN/RewP; ERN/CRN	27 OCD patients; 29 social anxiety disorder patients; 27 healthy controls	Study [49]
Feedback-monitoring abnormalities are directly linked to impaired decision-making under ambiguity.	IGT net scores correlated negatively with average FRN difference-wave amplitude, especially after disadvantageous choices. OCD showed poorer IGT learning.	Modified Iowa Gambling Task; decision-making under ambiguity	FRN; dFRN	24 OCD patients; 19 OCPD participants; 26 healthy controls	Study [56]
Subclinical OC traits may involve hyperactive negative-feedback monitoring that is behaviorally maladaptive during feedback-guided decision-making.	SOC participants showed poorer decision-making and larger FRN. In the SOC group, larger FRN responses were associated with worse IGT net scores.	Modified Iowa Gambling Task	FRN; source-localised aPFC/dACC activity	14 subclinical OC students; 14 low-OC controls	Study [57]
H. Mixed response- and feedback-monitoring components: ERN and FRN					
OC symptomatology may produce task-dependent error-monitoring abnormalities: hyperactive monitoring in simple response-conflict tasks but reduced feedback/error signals during adaptive learning.	Larger ERN during probabilistic learning was associated with better NoGo avoidance learning, but high OC symptoms were linked to smaller ERN/dERN/dFRN in learning and larger dERN in flanker.	Probabilistic reinforcement learning; flanker task	ERN/dERN, dFRN	Nonclinical high/low obsessive–compulsive symptom groups; final samples varied across probabilistic-learning and flanker analyses	Study [35]
Supports the reinforcement-learning view that performance-monitoring signals depend on how well stimulus–response mappings are learned; OCD-related hypermonitoring was not clearly confirmed.	No broad neuropsychological correlations were reported. Better learning was associated with a shift in the error signal from feedback to response: larger response ERN and smaller feedback ERN. OCD did not show a robust ERN enhancement.	Probabilistic learning; learning from response errors and feedback	Response ERN; feedback ERN	16 OCD patients; 16 healthy controls	Study [41]
OC symptomatology shows task-dependent error-monitoring abnormalities: hyperactive monitoring in simple conflict tasks but reduced learning-related monitoring during reinforcement learning.	Larger probabilistic-learning ERN predicted better NoGo avoidance learning. OC symptoms were associated with smaller reinforcement-learning ERN/dFRN but larger flanker ERN. OC symptoms also predicted poorer reversal learning.	Probabilistic learning; flanker task; reversal learning	ERN; dERN; dFRN; source-localised rACC/dACC activity	High- vs. low-OC undergraduate groups; resting EEG *n* = 107, probabilistic-learning ERP *n* = 70, flanker ERP subset *n* = 34	Study [48]

Note: Studies were grouped according to the primary ERP component or component family most closely aligned with the study objective and principal electrophysiological–cognitive finding. When a study assessed multiple components, all measured components were retained in the “ERP component(s)” column, but the study was presented only once. Studies directly comparing response- and feedback-monitoring components without a single dominant ERP outcome were classified as mixed-component studies. Abbreviations: aPFC, anterior prefrontal cortex; CNV, contingent negative variation; CRN, correct-response negativity; dACC, dorsal anterior cingulate cortex; dERN/ΔERN, error-minus-correct difference in error-related negativity; dFRN, difference-wave feedback-related negativity; ERN, error-related negativity; FRN, feedback-related negativity; ITPC, inter-trial phase coherence; LRP-r, response-locked lateralized readiness potential; Pc, correct-response positivity; Pe, error positivity; PPI, prepulse inhibition; rACC, rostral anterior cingulate cortex; RewP, reward positivity; RT, reaction time; SDMT, Symbol–Digit Modalities Test; SSRT, stop-signal reaction time; TMT, Trail-Making Test.

**Table 2:** EEGs studies.

Interpretation for the Review	EEG-Cognitive Function Relationship	Cognitive Domain/Task	EEG Components	Participants	Study
This is one of the strongest direct EEG–cognition studies for the review. It suggests that frontal low-frequency abnormalities, especially delta/theta source increases, may mark a cognitively impaired OCD subgroup, particularly with slowed visual–attentional and psychomotor processing.	Increased delta and theta sources were found mainly in OCD patients with cognitive impairment, whereas cognitively intact OCD patients did not differ from controls. The EEG abnormality was therefore linked more strongly to impaired cognitive performance than to OCD diagnosis alone.	Trail-Making Test A and B, Stroop Colour–Word Test, and D2 Test. The most relevant impairment was on TMT-A, reflecting processing speed, visual scanning, attention efficiency, and eye–hand coordination.	Resting-state EEG source activity analysed with sLORETA across delta, theta, alpha-1, alpha-2, beta-1, beta-2, and beta-3 bands. Main abnormalities involved increased frontal delta and theta current-source density.	20 patients with OCD and 15 healthy controls. OCD diagnosis based on ICD-10 criteria, confirmed by MINI. Within the OCD group, patients were divided into cognitively impaired and cognitively intact subgroups, mainly using TMT-A performance.	Study [58]
The study supports a hemispheric model of cognitive dysfunction in OCD. Right-sided EEG hypoactivity, especially in beta 2 activity, may be related to selective visual–spatial memory impairment, while verbal memory remains relatively preserved.	OCD patients showed poorer visual–spatial delayed recall but preserved verbal memory. Greater left-over-right beta 2 asymmetry, interpreted as reduced right-sided beta activity, was associated with poorer visual reproduction performance. The strongest relationship was at T5:T6 beta 2 asymmetry and visual reproduction.	Wechsler Memory Scale delayed recall measures: delayed visual reproduction for nonverbal/visual–spatial memory and delayed logical memory for verbal memory.	Resting-state eyes-closed EEG power and hemispheric asymmetry. Frequency bands: delta, theta, alpha, beta 1, and beta 2. Main focus: absolute power reductions and beta 2 hemispheric asymmetry, interpreted as right-hemisphere hypoactivity.	13 unmedicated, nondepressed OCD patients and 10 healthy controls. Patients were tested drug-free before entry into a clomipramine trial. Groups were similar in age and education.	Study [59]
This study is important because it frames OCD cognitive dysfunction as executive hypercontrol rather than simple impairment. Reduced alpha and increased beta associations with slowness suggest excessive controlled processing, inefficient monitoring, and reduced automaticity during executive performance.	Lower alpha1 relative power was associated with slower completion of the drawing Self-Ordered Pointing Task. Higher beta relative power was also associated with slower performance. OCD patients were not necessarily inaccurate, but they were slower on executive-control tasks.	Executive control and monitoring tasks, especially the Self-Ordered Pointing Task for drawings and words. Other tasks assessed conditional associative learning, attention, short-term memory, and recurring sequence learning.	Resting-state quantitative EEG spectral power. Bands included delta, theta1, theta2, alpha1, alpha2, beta1, beta2, and beta3. Core abnormality: reduced slow alpha/alpha1 power, especially over anterior regions; beta activity was also relevant.	32 drug-free patients with DSM-IV OCD and 32 matched healthy controls. All participants were right-handed. OCD patients had moderate-to-severe symptoms; analyses were repeated after excluding more depressed patients.	Study [60]
The study shows that cognitive dysfunction in OCD may appear at the level of preparatory neural regulation even when overt task performance is preserved. Reduced alpha/theta activity suggests impaired top-down preparation, inhibitory control, and flexible executive regulation.	OCD patients did not show poorer behavioural accuracy and were faster than controls, but they showed reduced prestimulus alpha and theta power. EEG measures correlated with WCST perseverative responses and category scores, linking abnormal oscillatory activity to set-shifting and perseveration.	Colour and shape visual discrimination tasks requiring selective attention and inhibition of irrelevant stimulus features. Executive function was also assessed with the Wisconsin Card Sorting Test.	Prestimulus oscillatory activity during visual discrimination. Main components: posterior/occipital alpha power and frontal theta power, especially Oz alpha and Fz theta.	Final EEG sample: 13 OCD patients and 15 healthy controls after exclusions. Most OCD patients were medicated with SSRIs, and some also received low-dose benzodiazepines.	Study [61]
This study suggests that visuospatial problems in OCD may reflect impaired executive organisation rather than pure visual-memory dysfunction. It also supports a lateralized frontal model: left frontal hyperactivation may be maladaptive, while right frontal activation may be compensatory.	Frontal alpha power predicted RCFT copy performance but not immediate or delayed recall. Greater left frontal activation was associated with poorer copy performance, whereas greater right frontal activation was associated with better copy performance.	Rey–Osterrieth Complex Figure Test: copy, immediate recall, and delayed recall. The copy score was interpreted as visuoconstructional and organisational ability; recall scores reflected visual memory.	Resting-state absolute alpha power from frontal, temporal, parietal, and occipital regions. Lower alpha was interpreted as greater cortical activation. Main focus: left and right frontal alpha activity.	23 patients with OCD, mostly medication-free. Diagnosis was based on DSM-IV criteria. Most patients were right-handed and free from major psychiatric or neurological comorbidities.	Study [62]

**Table 3:** Risk-of-bias assessment based on ROBINS-I.

Bias in Selection of the Reported Result	Bias in Measurement of Outcomes	Bias Due to Missing Data	Bias Due to Deviations from Intended Exposure	Bias in Classification of Exposure/Group Status	Bias in Selection of Participants	Bias Due to Confounding	Study
Moderate to serious	Moderate	Low to moderate	Low/not very applicable	Low to moderate	Moderate	Moderate	[21]
Serious	Moderate	Moderate	Low to moderate	Low	Moderate	Serious	[22]
Moderate	Low	Low to moderate	Low	Low	Moderate	Moderate	[23]
Serious	Moderate	Moderate	Low to moderate	Low	Moderate	Moderate to serious	[24]
Low	Low	Low	Low to moderate	Low	Moderate	Serious	[25]
Serious	Moderate	Low	Low	Low	Moderate	Serious	[26]
Serious	Moderate	Moderate	Low/not very applicable	Low to moderate	Moderate	Serious	[27]
Moderate	Moderate	Low	Low/not very applicable	Low	Moderate	Serious	[28]
Moderate	Moderate	Low to moderate	Low	Low	Moderate	Moderate	[29]
Moderate	Moderate	Low	Low/not very applicable	Low	Moderate	Serious	[30]
Serious	Moderate	Moderate	Low/not very applicable	Low to moderate	Serious	Serious	[31]
Moderate	Low to moderate	Moderate	Low/not very applicable	Low	Moderate	Moderate	[32]
Moderate	Low	Low to moderate	Moderate	Low	Moderate	Serious	[33]
Moderate	Low to moderate	Low	Low to moderate	Low	Moderate	Moderate	[34]
Moderate	Low	Serious	Low	Moderate	Moderate	Serious	[35]
Moderate	Moderate	Moderate	Moderate	Low to moderate	Moderate	Serious	[36]
Serious	Low to moderate	Moderate	Low to moderate	Low	Moderate	Serious	[37]
Moderate	Low to moderate	Low to moderate	Low/not very applicable	Low	Moderate	Serious	[38]
Moderate	Low	Moderate	Low	Low	Moderate	Serious	[39]
Serious	Moderate	Low to moderate	Low/not very applicable	Low	Moderate	Serious	[40]
Moderate	Low to moderate	Low	Low to moderate	Low	Moderate	Serious	[41]
Moderate	Low	Low to moderate	Low/not very applicable	Low	Moderate	Moderate	[42]
Moderate	Moderate	Low	Moderate	Low to moderate	Moderate	Serious	[43]
Moderate	Moderate	Moderate	Low/not very applicable	Moderate to Serious	Moderate	Serious	[44]
Moderate	Moderate	Moderate	Low	Low	Moderate	Moderate	[45]
Moderate	Low	Moderate	Low	Low	Moderate	Serious	[46]
Moderate	Moderate	Serious	Moderate	Moderate	Moderate to Serious	Serious	[47]
Serious	Moderate	Serious	Low to moderate	Moderate	Serious	Serious	[48]
Moderate	Low to moderate	Moderate	Low	Low to moderate	Moderate	Serious	[49]
Moderate	Low	Low to moderate	Low to moderate	Low	Moderate	Moderate	[50]
Moderate	Low to moderate	Moderate	Low/not very applicable	Low to moderate	Moderate to Serious	Serious	[51]
Serious	Moderate	Low to moderate	Low to moderate	Moderate	Moderate	Serious	[52]
Moderate	Low to moderate	Moderate	Low	Moderate	Moderate	Serious	[53]
Moderate to Serious	Low to moderate	Moderate	Low	Low to moderate	Moderate	Serious	[54]
Serious	Moderate	Moderate	Low	Low to moderate	Moderate	Serious	[55]
Moderate	Low to moderate	Low	Low to moderate	Moderate	Serious	Serious	[56]
Moderate	Low to moderate	Moderate	Low	Low to moderate	Moderate	Moderate	[57]
Moderate to Serious	Moderate	Low to moderate	Low/not very applicable	Low to moderate	Moderate	Serious	[58]
Serious	Serious	Moderate	Low/not very applicable	Low to moderate	Serious	Serious	[59]
Moderate to Serious	Moderate	Moderate	Low/not very applicable	Low to moderate	Moderate to Serious	Serious	[60]
Serious	Moderate	Moderate	Low	Low to moderate	Moderate	Serious	[61]
Serious	Moderate	Low	Low/not very applicable	Moderate	Moderate	Serious	[62]

## Data Availability

No new data were created or analysed in this study. Data sharing is not applicable to this article.

## Supplementary Materials

The following supporting information can be downloaded at: https://www.mdpi.com/article/10.3390/jcm15155994/s1, File S1: PRISMA 2020 Checklist [234].
